# A new triazine bearing a pyrazolone group capable of copper, nickel, and zinc chelation†

**DOI:** 10.1039/c7ra09459k

**Published:** 2018-01-16

**Authors:** Akop Yepremyan, Arshad Mehmood, Samantha M. Brewer, Madalyn M. Barnett, Benjamin G. Janesko, Giridhar Akkaraju, Eric E. Simanek, Kayla N. Green

**Affiliations:** Department of Chemistry and Biochemistry, Texas Christian University Fort Worth Texas 76129 USA kayla.green@tcu.edu e.simanek@tcu.edu; Department of Biology, Texas Christian University Fort Worth Texas 76129 USA

## Abstract

Interest in inorganic applications of triazines is growing. In this report, metal complexes of copper(ii), nickel(ii), and zinc(ii) and a novel class of chelates composed of a triazine ring substituted with a hydrazine group and pyrazolone are evaluated using spectrophotometric methods, single crystal X-ray diffractometry, and electrochemistry. Complexes with copper(ii) include a single chelate and chloride ion(s)/water to satisfy a trigonal bipyramidal coordination sphere. The nickel(ii) and zinc(ii) complexes are composed of two chelating groups that adopt an octahedral geometry around the metal ion. Irreversible redox activity was observed with the copper(ii) complex but no redox activity was observed with the ligand alone or zinc(ii) and nickel(ii) complexes. Use of the coumarin carboxylic acid assay shows that the ligand motif is capable of preventing redox cycling of copper in biological conditions and could thus serve as an antioxidant preventative agent. Cellular toxicity studies show that the new triazine molecule could have therapeutic applications in the μM concentration range based on the measured EC_50_ = 1.183 ± 0.002 mM. Altogether this work shows that by merging triazine chemistry into inorganic compounds, there is potential to explore a range applications thanks to the new architecture.

## Abstract 2

A new triazine bearing a pyrazolone moiety and metalation with first row transition metals, copper(ii), nickel(ii), and zinc(ii) is explored.

## Graphical abstract

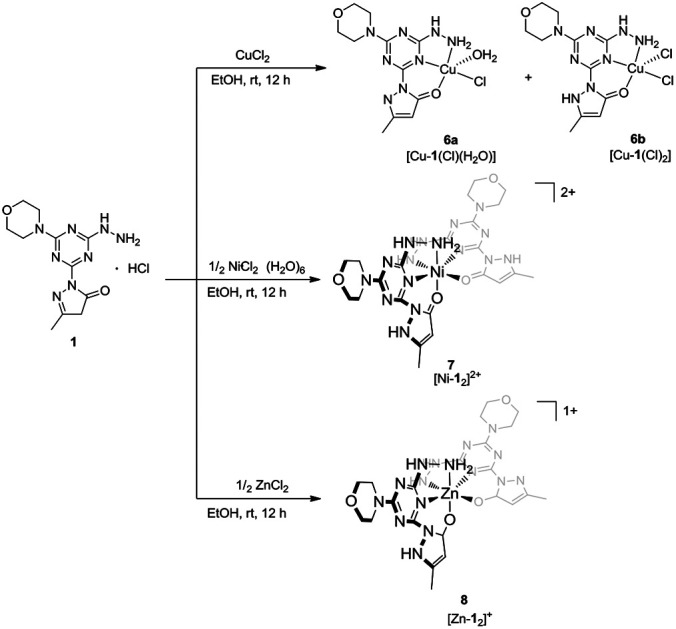

## Introduction

[*s*]-Triazine groups appear in reactive dyes,^1^ combinatorial libraries of drug candidates,^2^ linkers for PEGylation, building blocks for molecular recognition motifs,^3^ and as chelators,^4^ amongst others.^5^ Our interest in triazines stems from their utility in the construction of dendrimers.^6^ For use in drug delivery,^7^ we have explored various strategies for the attachment of bioactive groups to triazine dendrimers including esters,^8^ disulfides,^9^ maleimides,^10^ and most recently, hydrazones.^11^

**Chart 1:**
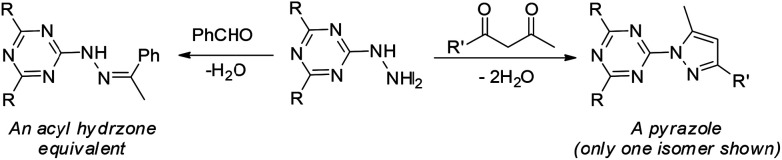
The central triazinylhydrazine can be condensed with an aldehyde (left) to form a hydrazone or with a 1,3-diketone (right) to create a pyrazole. R = NHCH_2_CH_2_OCH_2_CH_2_OH. R′ is undefined.

**Chart 2:**
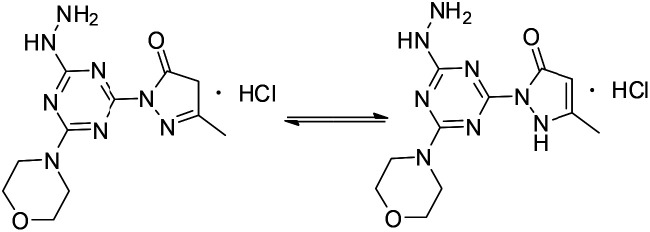
Pyrazolone 1 (imine, left; enamine, right).

The derivatization of a triazine with a hydrazine group offers a site for the condensation of a carbonyl-containing aldehyde or ketone through a labile linkage sensitive to acid-catalyzed hydrolysis (Chart 1).^11^ Stability can be tuned modestly with the choice of the carbonyl donor. The use of 1,3-dicarbonyl-containing compounds, however, offers an opportunity to dramatically increase the stability of the linkage due to the formation of a pyrazole ring. While model studies with curcumin bore this hypothesis out,^12^ we chose to pursue acetoacetic acid amides believing that the acetoacetyl group could be installed readily onto a range of bioactive cargoes. As a model system, *N*-methylacetoacetamide was investigated with the desire that upon aromatization, the *N*-methyl amino group would be preserved. Instead of the desired aminomethylpyrazole, a pyrazolone, molecule 1, was obtained in high yields (Chart 2).

Inspired by the work of Melman and others,^4,13–15^ wherein triazine rings were *N*-substituted with hydroxylamine and employed for chelation to form an Fe(iii) 5,5,5 ring complex; here, we explore copper(ii), nickel(ii) and zinc(ii) complexes of 1, in which the triazine has been functionalized to include a hydrazine and a pyrazolone moiety, leading to a unique 6,5,5 oxygen to metal binding ring system. The preference for this specific binding may lead to a more effective chelating agent compared to triazine derivatives that have been explored in the past. Such a novel metal binding motif has immediate medicinal applications. Unregulated transition metal ions such as copper have been implicated in a range of diseases. For example, modulation of brain–metal homeostasis has been associated with cognitive decline and neurodegeneration.^16–19^ Reactive oxygen species can be rapidly generated by Fenton type reduction/oxidation reactions if redox–active transition metal ions are allowed to react with the copious amounts of oxygen in brain tissue.^20^ This imbalance is exacerbated when the enzymes, often incorporating metal-ions themselves, responsible for maintaining homeostasis of oxidative stress are modified.^21,22^ Therefore, driven by the long-standing interest in developing small molecules capable of controlling un-regulated metal-ions and mitigating imbalanced redox activity *in vivo*, we set-out to establish the metal binding coordination of molecule 1. Cell studies to establish the toxicity of molecule 1 were also carried out to evaluate the potential for such future working involving biological/therapeutic applications. This is the first report of a pyrazolone and triazine combined and its chelates to the best of our knowledge and serves as a cornerstone for future studies of new, metal-binding triazine based molecules.

## Results and discussion

### Synthesis of molecule 1

**Scheme 1:**
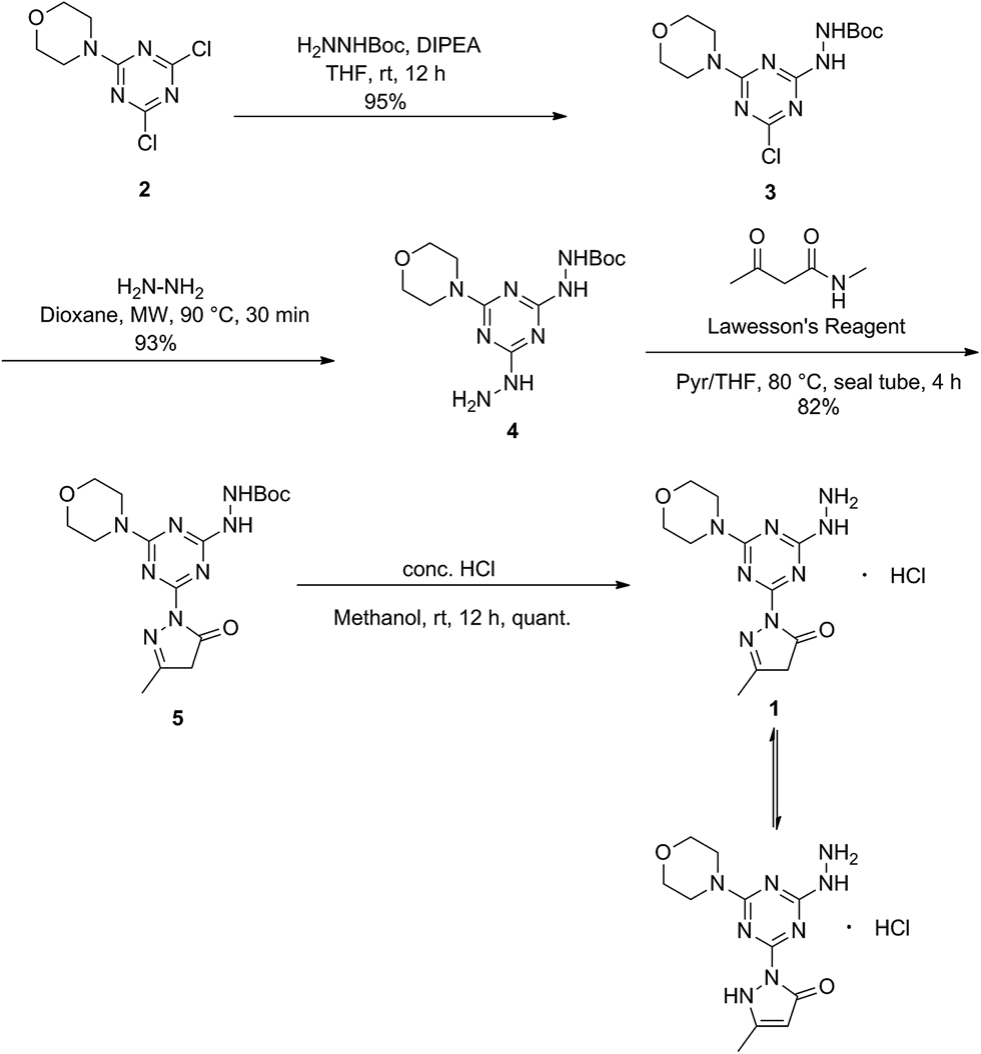
Synthesis of 1.

Molecule 1 was produced in four steps through the stepwise substitution of the trichlorotriazine ring (Scheme 1). First, cyanuric chloride is treated with morpholine at 0 °C to obtain intermediate dichlorotriazine 2.^23^ Subsequent nucleophilic substitution with BOC-hydrazine at room temperature yields 3,^11^ followed by the addition of hydrazine using microwave irradiation to afford intermediate 4.^7^ Reaction with acetoacetamide in the presence of Lawessons's reagent gives 5, which upon deprotection produces 1.^24^ We attribute the formation of the pyrazolone and loss of methylamine as evidence that the reaction is thermodynamically controlled. The product and intermediates were characterized by ^1^H and ^13^C NMR as well as mass spectrometry (ESI). Conveniently, 1 is isolated as a white powder. Characterization of molecule 1 in D_2_O indicates that the imine species is predominant when isolated. However, an equilibrium between the imine and enamine forms is generated in aqueous solution after 18 hours. A ratio of 2 : 3 is observed by comparison of the methyl protons of the pyrazolone ring. The α-CH_2_ of the imine tautomer is found on *δ* 3.80–3.50 ppm region and the conjugate isomer enamine shows a resonance at *δ* 5.4 ppm for compounds 5 and 1 in CDCl_3_ and DMSO-d_6_ respectively. From HSQC analysis the hydroxypyrazole tautomer was not present. No trace of aminomethyl pyrazole species was observed in ^1^H, ^13^C, HSQC NMR in D_2_O and DMSO-d_6_ of molecule 1 (Fig. S13–S19†). IR spectra contained (C=O) vibrations (*ν*_C=O_ = 1638 cm^−1^) within the expected range to further confirm the presence of the enamine and imine species.

### Synthesis of metal complexes

**Scheme 2:**
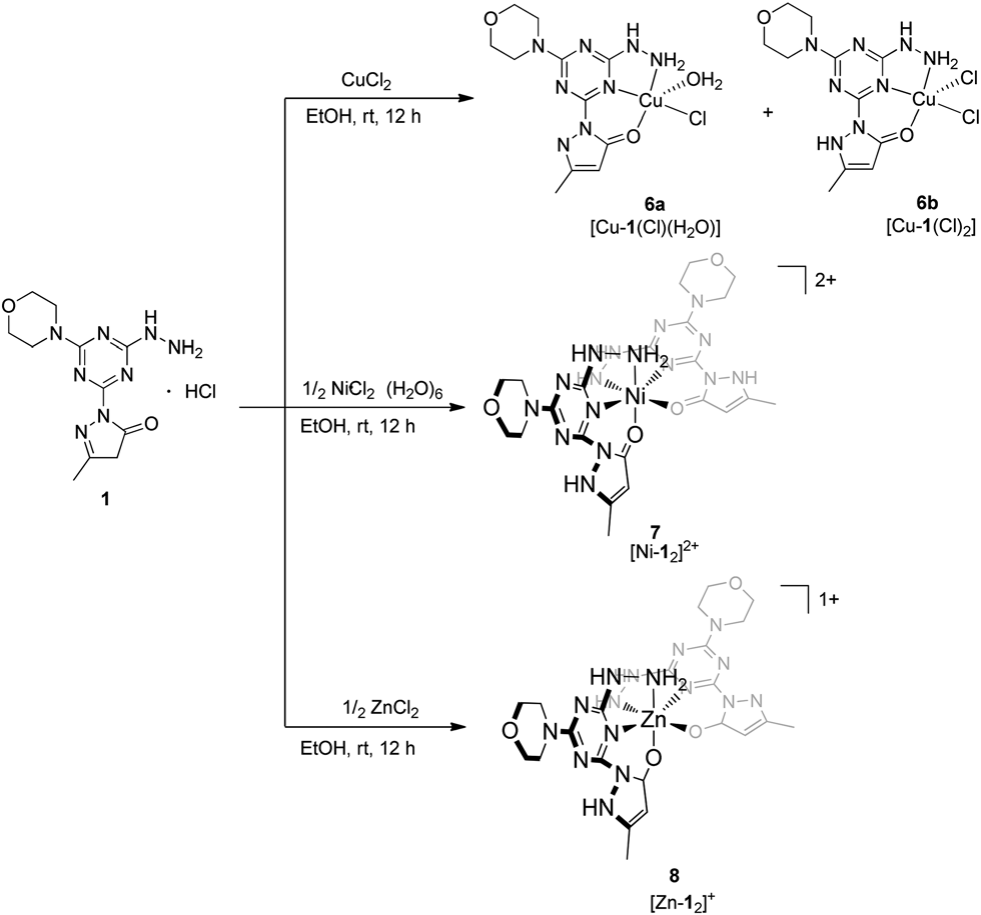
Synthesis of transition metal complexes 6, 7, 8 isolated by the addition of copper(ii), nickel(ii), and zinc(ii) chloride salts to molecule 1.

The arrangement of N- and O-atoms in molecule 1 is reminiscent of metal binding molecules derived from triazine cores reported by Melman.^4,13–15^ Therefore, metalations of 1 with the biologically relevant metals copper(ii), nickel(ii), and zinc(ii) were evaluated to define the coordination sphere accessible with this new ligand construct. A copper(ii) complex of 1 was achieved by the addition of one equivalent of anhydrous copper chloride (CuCl_2_) in absolute EtOH to a solution of 1 and stirred overnight.^18^ The resulting green powder was isolated, crystalized, and characterized as complex 6 (Scheme 2), a mixture of [Cu-1(Cl)(H_2_O)] and [Cu-1(Cl)_2_], 6a and 6b respectively. The syntheses of purple complex 7 [Ni-1_2_]^2+^ and white complex 8 [Zn-1_2_]^+^ were achieved under similar conditions using 1/2 equivalent of nickel chloride hexahydrate (NiCl_2_·6H_2_O) and zinc chloride (ZnCl_2_), respectively.

### Solid state structures of copper(ii), nickel(ii), and zinc(ii) complexes (6–8)

**Fig. 1:**
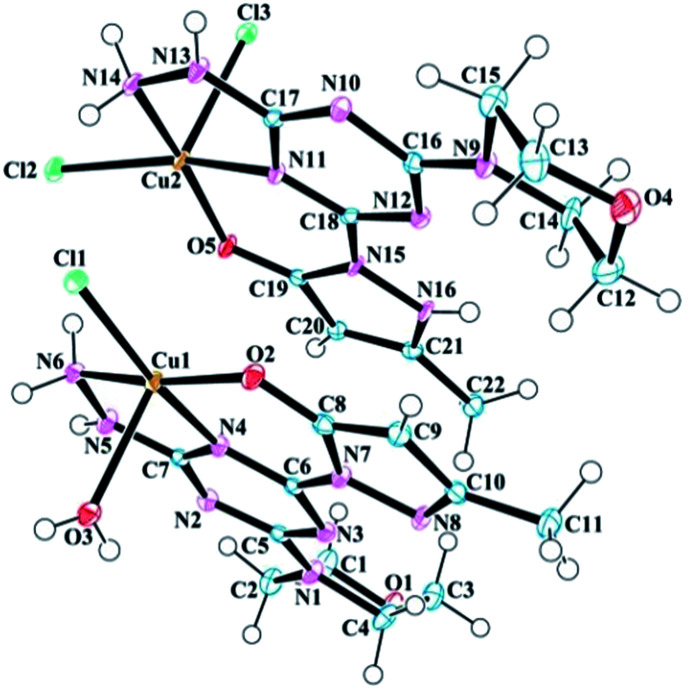
Molecular structure and numbering scheme for 6. The 6a species is positioned below 6b in this orientation. Thermal ellipsoids are drawn at 50% probability. The solvent molecules have been omitted for clarity.

**Fig. 2:**
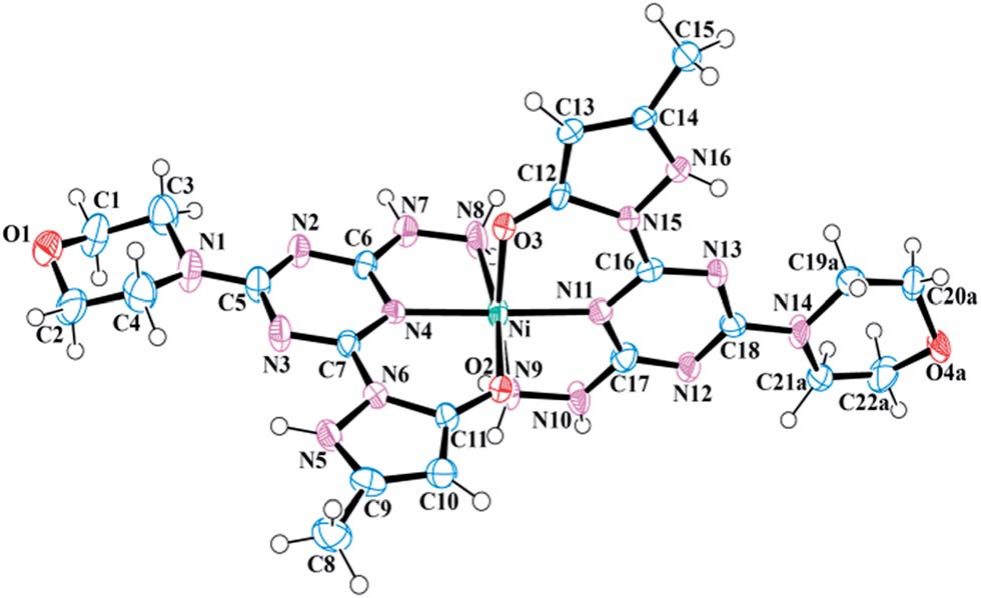
Molecular structure and numbering scheme for 7. Thermal ellipsoids are drawn at 50% probability. The counter ions and solvent molecules have been omitted for clarity.

**Fig. 3:**
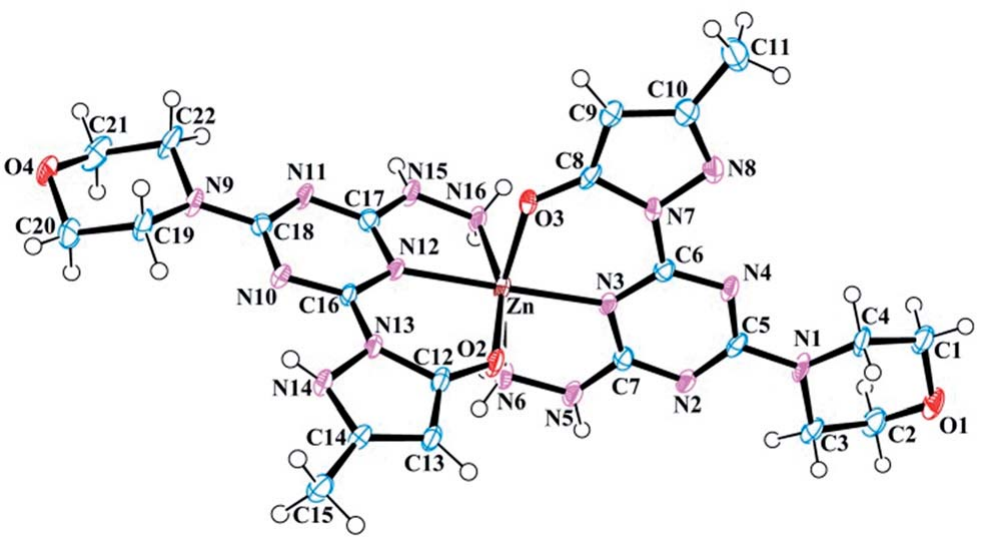
Molecular structure and numbering scheme for 8. Thermal ellipsoids are drawn at 50% probability. The counter ions and solvent molecules have been omitted for clarity.

Samples of complexes 6–8 suitable for analysis by single crystal X-ray diffraction were obtained by slow evaporation of aqueous solution made from previously isolated powders. The data resulted in the solid states structures shown in (Fig. 1–3). Throughout the series, molecule 1 was observed as either a neutral or anionic ligand to the transition metal cations explored. The neutral ligand was observed as the keto form, as shown in Chart 2 (right). The anionic form of molecule 1 is achieved by removal of the –NH proton from the pyrazole moiety. In both cases, the C=O bond lengths (∼1.3 Å) in complexes 6–8 are slightly longer than a traditional C=O bond and the observation is attributed to the interaction of the O-atom with the metal center as well as resonance between the imine and enamine forms shown in Chart 2.

The asymmetric unit of the copper complex 6, contains two crystallographically distinct copper(ii) complexes (6a, [Cu-1(Cl)(H_2_O)] and 6b, [Cu-1(Cl)_2_]) with an overall neutral charge in addition to four lattice H_2_O molecules (Fig. 1). The coordination sphere of 6a (Cu1 site in Fig. 1) contains one mono-anionic chelating tridentate molecule 1, one equatorial chloride, and one apical aqua ligand to complete the coordinative-motif. The 6b component (Cu2 site in Fig. 1) consists of one neutral tridentate molecule 1 and two chloride ligands (one equatorial and one apical). This composition is consistent with elemental analysis results as well. Both penta-coordinated copper sites exhibit square pyramidal geometries (Fig. S19†) based on calculation of the angular geometric parameter (*τ*) for Cu1 (0.06) and Cu2 (0.08).^25^ The two copper complexes interact with one another in the unit cell through N–H⋯Cl hydrogen bonds between the hydrazine hydrogen atoms and chloride ligands (Table 1). The pyrazole, triazine, and hydrazine groups in both coordination spheres are coplanar with the 5,6 bicyclic ring system. Psuedo-planarity of the molecule is maintained as the morpholine ring adopts a chair conformation that is substituted equatorially by the trazine. The Cu2–Cl3 (2.5706 Å) bond is longer than Cu2–Cl2 (2.2778 Å) and Cu1–Cl1 (2.2767 Å), which can be associated to the involvement of Cl2 and Cl1 in the aforementioned hydrogen bonding with the hydrazine hydrogen atoms. Selected bond lengths and bond angles involving metal centers are given in Table 1.

**Table 1:** Selected bond lengths (Å) and angles (°) of 6

**Bond lengths**			
Cu1–Cl1	2.2767 (7)	Cu2–Cl2	2.2778 (7)
Cu1–O2	1.910 (2)	Cu2–Cl3	2.5706 (8)
Cu1–O3	2.306 (2)	Cu2–O5	1.9418 (19)
Cu1–N4	1.953 (2)	Cu2–N11	1.962 (2)
Cu1–N6	2.018 (2)	Cu2–N14	2.013 (2)

**Bond angles**			
Cl1–Cu1–O3	100.94 (7)	C7–N4–Cu1	115.40 (18)
O2–Cu1–Cl1	90.43 (6)	C6–N4–Cu1	129.78 (19)
O2–Cu1–O3	100.56 (9)	Cl2–Cu2–Cl3	103.37 (3)
O2–Cu1–N4	91.94 (9)	O5–Cu2–Cl2	92.52 (6)
O2–Cu1–N6	166.42 (10)	O5–Cu2–Cl3	99.27 (6)
N4–Cu1–Cl1	170.32 (7)	O5–Cu2–N11	90.89 (9)
N4–Cu1–O3	87.85 (9)	O5–Cu2–N14	166.34 (9)
N4–Cu1–N6	81.45 (10)	N11–Cu2–Cl2	161.34 (7)
N6–Cu1–Cl1	94.23 (7)	N11–Cu2–Cl3	94.16 (7)
N6–Cu1–O3	91.09 (9)	N11–Cu2–N14	80.60 (10)
Cu1–O3–H3A	115 (3)	N14–Cu2–Cl2	92.30 (7)
Cu1–O3–H3B	136 (4)	N14–Cu2–Cl3	92.03 (8)
C8–O2–Cu1	125.16 (18)	C19–O5–Cu2	126.25 (18)
Cu1–N6–H6A	113 (2)	C17–N11–Cu2	115.50 (17)
Cu1–N6–H6B	109 (2)	C18–N11–Cu2	130.81 (19)
N5–N6–Cu1	109.22 (17)	Cu2–N14–H14A	116 (2)
N13–N14–Cu2	110.25 (17)	Cu2–N14–H14B	106 (3)

As shown in Table 2, both coordination complexes in the unit cell of 6 are rich in intermolecular hydrogen bonding. The crystal structure is stabilized by a number of hydrogen bonding and van der Waals interactions involving the main structural unit and solvent H_2_O molecules. The H3A atom of the ligand H_2_O molecule in the Cu1 assembly forms a strong hydrogen bond with N8 of the neighbouring (−*x* + 1, −*y* + 1, −*z* + 1) pyrazole moiety. This strong interaction reduces the H3B–O3–H3A angle to 103.077° from the typical value of 104.5° expected for a perfect tetrahedron. The O1 of morpholine ring forms a strong hydrogen bond with a H_2_O molecule, which works as a bridge between O1 and O2 of neighboring (*x* + 1, *y* + 1, *z*) pyrazole moiety. This bifurcated strong hydrogen bonding of O6 plays a bridging role in the molecular assembly by holding the three adjacent molecules together through a network of O–H⋯O hydrogen bonds and is a major contributor of crystal packing. Other important hydrogen bonds in 6 are listed in Table 2.

**Table 2:** Selected hydrogen bond geometry parameters (Å, °) for 6

*D*–H⋯*A*	*D*–H	H⋯*A*	*D*⋯*A*	*D*–H⋯*A*
O3–H3*A*⋯N8^a^	0.69 (4)	2.15 (4)	2.830 (3)	173 (4)
O3–H3*B*⋯Cl2^b^	0.71 (5)	2.74 (5)	3.395 (3)	153 (4)
N5–H5⋯O7^c^	0.79 (3)	2.10 (4)	2.842 (4)	156 (3)
N6–H6*A*⋯Cl2	0.85 (4)	2.53 (4)	3.351 (3)	163 (3)
N6–H6*B*⋯Cl1^b^	0.92 (4)	2.33 (4)	3.220 (3)	163 (3)
N13–H13⋯O8*A*	0.83 (3)	2.07 (4)	2.887 (14)	168 (3)
N13–H13⋯O8*B*	0.83 (3)	2.12 (3)	2.894 (6)	155 (3)
N14–H14*A*⋯Cl3^e^	0.86 (4)	2.41 (4)	3.226 (2)	158 (3)
N14–H14*B*⋯Cl1	0.88 (4)	2.58 (4)	3.240 (3)	132 (3)
O6–H6*C*⋯O2^d^	0.78 (4)	2.07 (5)	2.835 (4)	169 (4)
O6–H6*D*⋯O1	0.68 (5)	2.13 (5)	2.777 (4)	160 (5)
O7–H7*A*⋯Cl1^a^	0.76 (5)	2.95 (5)	3.467 (3)	127 (4)
O7–H7*B*⋯O6	0.72 (5)	2.08 (5)	2.783 (4)	166 (6)
O8*A*–H8*AA*···Cl2^e^	0.87 (5)	2.61 (5)	3.471 (14)	171 (4)
O8*B*–H8*AA*···Cl2^e^	0.70 (5)	2.61 (5)	3.232 (6)	148 (5)
O8*A*–H8*AB*···Cl3^e^	1.12 (5)	2.63 (6)	3.718 (14)	165 (5)
O8*B*–H8*AB*···Cl3^e^	0.57 (5)	2.63 (6)	3.179 (8)	164 (7)
N16–H16⋯Cl3^f^	0.82 (4)	2.42 (4)	3.187 (2)	157 (3)

a −*x* + 1, −*y* + 1, −*z* + 1.

b −*x*, −*y* + 1, −*z* + 1.

c *x*−1, *y*, *z*.

d −*x*, −*y* + 1, −*z* + 2.

e *x* + 1, *y* + 1, *z*.

f −*x* + 1, −*y* + 1, −*z* + 2.

The asymmetric unit of 7 is composed of a [Ni-1_2_]^2+^ complex, two Cl^−^ ions, and three H_2_O molecules (Fig. 2). The nickel(ii) metal center of 7 is bound by two neutral molecule 1 units and exhibits a distorted octahedral geometry (Fig. S22†). Like 6, the pyrazole, triazine, and hydrazine rings of each ligand are nearly coplanar to chelate rings. The angle between the metal center and coordinated oxygen atoms (O3 and O2) of 1 is 91.364°, whereas the angle between N11–Ni–N8 is 97.099°, which confirms the distortion of octahedral geometry. The bite angle formed by the two coordinated triazine nitrogen atoms of 1 and the metal center (N4–Ni–N11) is 176.929°, which is smaller than the ideal value of 180°, and can again, be attributed as the consequence of distorted geometry at metal center. Selected bond lengths and bond angles have been provided in Table 3.

**Table 3:** Selected bond lengths (Å) and angles (°) of 7

**Bond lengths**			
Ni–O2	2.053 (5)	Ni–N8	2.096 (7)
Ni–O3	2.034 (6)	Ni–N9	2.092 (7)
Ni–N4	2.018 (6)	Ni–N11	2.000 (6)

**Bond angles**			
O2–Ni–N8	169.4 (2)	N11–Ni–O2	93.4 (2)
O2–Ni–N9	90.1 (2)	N11–Ni–O3	90.0 (2)
O3–Ni–O2	91.3 (2)	N11–Ni–N4	177.0 (3)
O3–Ni–N8	88.9 (3)	N11–Ni–N8	97.1 (3)
O3–Ni–N9	170.1 (2)	N11–Ni–N9	80.1 (2)
N4–Ni–O2	89.4 (2)	C11–O2–Ni	126.4 (5)
N4–Ni–O3	91.0 (2)	C12–O3–Ni	125.8 (5)
N4–Ni–N8	80.0 (2)	N7–N8–Ni	108.7 (5)
N4–Ni–N9	98.9 (2)	N10–N9–Ni	108.2 (5)
N9–Ni–N8	91.5 (3)	C16–N11–Ni	131.2 (5)
C17–N11–Ni	116.0 (5)		

The N5 nitrogen of each pyrazole moiety in 7 is protonated, thus yielding a neutral ligand. Two chloride counterions serve as charge balance for complex 7. Like 6 the pyrazole, triazine, and hydrazine rings of each ligand are nearly coplanar: the largest deviation from the Ni–N6–N8 plane is 0.219 Å by C5. In addition, there are various hydrogen bonding interactions present in the crystal structure of 7. The pyrazole nitrogen atom (N16) of one molecule 1 has a strong hydrogen bonding interaction with O2 of the neighboring (−*x* + 1, −*y* + 1, −*z* + 1) pyrazole moiety, whereas the pyrazole nitrogen (N16) of the remaining 1 interacts through hydrogen bonding with the Cl^−^ counter ion. The N8 of the hydrazine ring is hydrogen bonded to the solvent H_2_O molecule, which is further connected to the Cl^−^ counter ion. This hydrogen bonding interaction plays a major role in crystal packing. Furthermore, N10 of hydrazine donates H10 to O1 of neighboring (*x* + 1, *y*, *z*) morpholine at a distance of 1.94 Å and forms a strong hydrogen bonding interaction. Table 4 lists the classical hydrogen bond geometries present in the crystal structure of 7.

**Table 4:** Selected hydrogen bond geometry parameters (Å, °) for 7

*D*–H⋯*A*	*D*–H	H⋯*A*	*D*⋯*A*	*D*–H⋯*A*
N8–H8*A*⋯O5*A*^b^	0.91	2.38	3.126 (16)	140
N8–H8*A*⋯O5*B*^b^	0.91	2.52	3.33 (2)	148
N9–H9*A*⋯Cl2^c^	0.91	2.33	3.224 (7)	169
N9–H9*B*⋯O6*A*	0.91	2.21	3.10 (8)	165
N9–H9*B*⋯O6*A*^a^	0.91	2.59	3.36 (9)	143
N9–H9*B*⋯O6*B*	0.91	2.27	3.11 (9)	152
N7–H7⋯Cl1*A*	0.93 (8)	2.1 (4)	3.0 (4)	160 (15)
N7–H7⋯Cl1*B*	0.93 (8)	2.1 (5)	3.0 (5)	162 (16)
N10–H10⋯O1^d^	0.89 (9)	1.94 (10)	2.793 (9)	161 (8)
N16–H16⋯O2^e^	0.79 (8)	2.16 (8)	2.844 (8)	144 (7)
N5–H5⋯Cl2	0.94 (9)	2.23 (9)	3.093 (7)	153 (7)

a −*x* + 1, −*y*, −*z* + 2.

b −*x* + 1, −*y*, −*z* + 1.

c −*x*, −*y* + 1, −*z* + 2.

d *x* + 1, *y*, *z*.

e −*x* + 1, −*y* + 1, −*z* + 1.

One [Zn-1_2_]^+^ complex is observed in the asymmetric unit of 8 along with nine H_2_O molecules and a Cl^−^ counter ion (Fig. 3). Three H_2_O molecules are disordered with multiple occupancies within the crystal lattice. Like 7, the zinc(ii) metal center of 8 exhibits a distorted octahedral geometry (Fig. S26†) and is bound to two ligands, 1. Interestingly, the zinc(ii) ion is bound to one neutral molecule 1 and one anionic molecule 1, resulting in an overall +1 charge for the complex. The octahedral geometry of 8 compared to 7 is more distorted. For example, the right angle of the N3–Zn–N16 bond angle of 8 is increased to 103.11° compared to 97.099° in 7 and 90° in a perfect octahedron. The angle formed by the two coordinated triazine nitrogen atoms and the metal center (N12–Zn–N3) is 177.511°. The bite angle formed between the coordinated oxygen, metal center, and nitrogen of hydrazine (O3–Zn–N6) is non-linear at 165.00°, which is larger than the same angle (O2–Zn–N16) in the other ligand by 2.1°. This reduction can be attributed to the involvement of the latter ligand in strong hydrogen bonding with H_2_O molecules, which is absent in the former. The bond length of the apical hydrazine nitrogen atoms (N8 and N9) with the metal center is larger than the other metal nitrogen bonds. Pertinent bond lengths and bond angles involving the metal center are listed in Table 5.

**Table 5:** Selected bond lengths (Å) and angles (°) of 8

**Bond lengths**			
Zn–O2	2.066 (4)	Zn–N6	2.191 (5)
Zn–O3	2.048 (4)	Zn–N12	2.092 (4)
Zn–N3	2.082 (4)	Zn–N16	2.172 (5)

**Bond angles**			
O2–Zn–N3	92.59 (15)	N12–Zn–N6	103.94 (17)
O2–Zn–N6	85.21 (19)	N12–Zn–N16	78.19 (16)
O2–Zn–N12	86.38 (15)	N16–Zn–N6	91.4 (2)
O2–Zn–N16	162.92 (16)	C12–O2–Zn	128.8 (3)
O3–Zn–O2	98.17 (15)	C8–O3–Zn	128.2 (3)
O3–Zn–N3	87.01 (15)	Zn–N16–H16A	118 (3)
O3–Zn–N6	165.00 (15)	Zn–N16–H16B	108 (4)
O3–Zn–N12	90.89 (15)	N15–N16–Zn	107.5 (3)
O3–Zn–N16	89.43 (18)	Zn–N6–H6A	120 (4)
N3–Zn–N6	78.21 (17)	Zn–N6–H6B	107 (4)
N3–Zn–N12	177.51 (18)	N5–N6–Zn	108.0 (3)
N3–Zn–N16	103.11 (17)	C6–N3–Zn	131.2 (3)
C17–N12–Zn	114.5 (3)	C16–N12–Zn	131.5 (3)
C7–N3–Zn	114.7 (3)		

The solid state structure of 8 contains various hydrogen bonding interactions. Within the unit cell of 8, a Cl^−^ counter ion is linked to main structural motif through a bridging H_2_O molecule. The N15 of hydrazine donates H15 to morpholine O1 of the neighbouring complex (*x* − 1, *y*, *z*) at 2.878 Å apart to form a one-dimensional propeller-like chain structure, which is responsible for crystal packing. The N14 of pyrazole donates H14 to O3 of neighbouring (−*x*, −*y* + 1, −*z* + 1) pyrazole and *vice versa* at the distance of 2.761 Å to form a closed hydrogen bonding interaction. A lattice H_2_O molecule makes a bifurcated contact with N16 of pyrazole and the Cl2 counter ion, which serves as a bridge between them. This contact is also a potential candidate, responsible for crystallization. Other important hydrogen bonding interactions in 8 are listed in Table 6.

**Table 6:** Selected hydrogen bond geometry parameters (Å, °) for 8

*D*–H⋯*A*	*D*–H	H⋯*A*	*D*⋯*A*	*D*–H⋯*A*
N5–H5⋯O7^a^	0.89 (6)	2.02 (6)	2.883 (7)	162 (6)
N6–H6A⋯Cl2^b^	0.85 (6)	2.56 (6)	3.334 (6)	152 (5)
N6–H6*B*⋯O10*B*^a^	0.90 (7)	2.04 (7)	2.933 (14)	169 (6)
N15–H15⋯O1^c^	0.81 (6)	2.11 (6)	2.878 (6)	158 (5)
N16–H16*A*⋯Cl2^b^	0.93 (5)	2.64 (5)	3.367 (6)	136 (4)
N16–H16*B*⋯O5	0.81 (6)	2.12 (6)	2.910 (7)	166 (5)
O5–H5*A*⋯Cl2	0.99 (6)	2.19 (6)	3.172 (6)	172 (5)
N14–H14⋯O3^d^	0.81 (7)	1.97 (7)	2.761 (5)	164 (7)
O5–H5*B*⋯O11^c^	0.99 (2)	2.50 (14)	3.416 (9)	154 (25)

a −*x* + 1, *y* + 1/2, −*z* + 3/2.

b −*x*, −*y* + 1, −*z* + 2.

c *x* − 1, *y*, *z*.

d −*x*, −*y* + 1, −*z* + 1.

### UV-vis spectroscopy

**Fig. 4:**
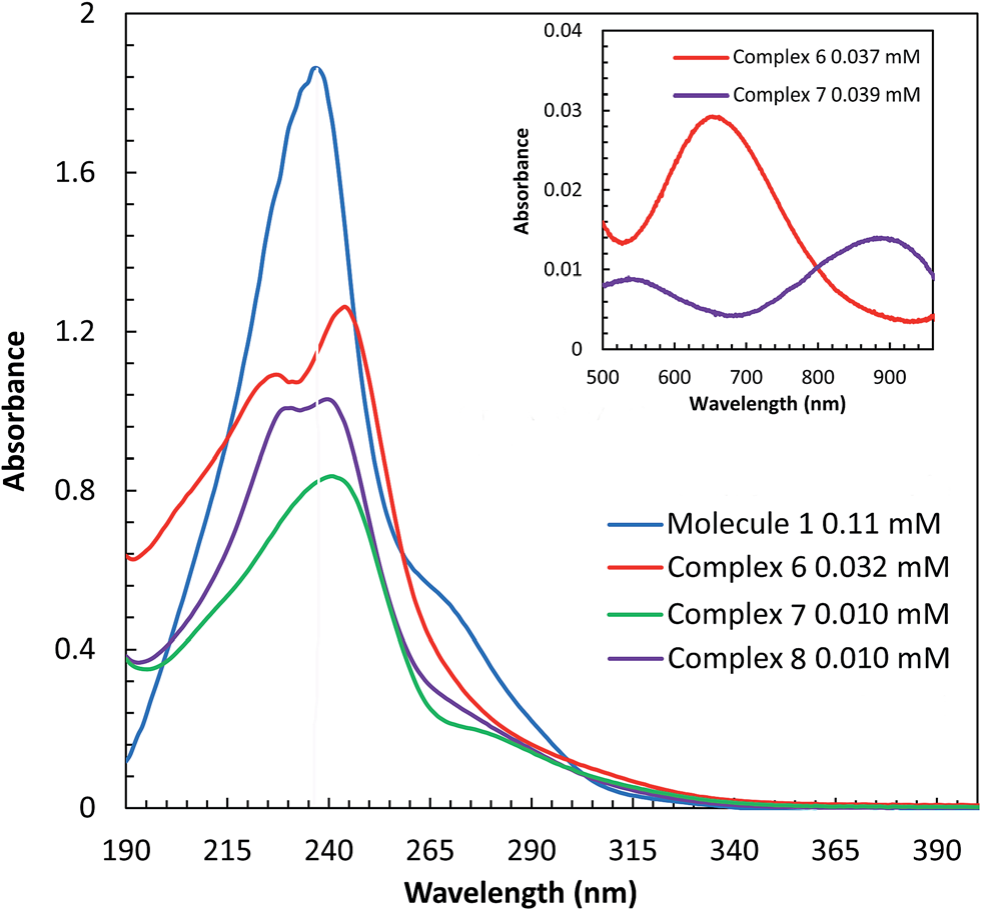
UV-vis spectra π → π* transitions of 1 and complexes 6, 7, and 8 in H_2_O. The inset displays the d → d transition of complexes 7 and 8.

The UV-visible spectra of molecule 1 and complexes 6, 7, and 8 were obtained in H_2_O at room temperature. In aqueous solution, complex 6 is green, 7 is purple, and 8 is colorless. Spectroscopic comparisons of π → π* and d → d transitions are shown in (Fig. 4) and the insert, respectively. Molecule 1 contains absorbance bands at 237 nm (17 413 cm^−1^ M^−1^) and a shoulder at 252 nm (6723 cm^−1^ M^−1^), derived from π → π* transitions. Upon addition of copper(ii), a blue shift is observed in the two π → π* absorbance events as compared to the π → π* transition of the ligand. The π → π* transitions for complex 6 are observed at 227 nm (30 285 cm^−1^ M^−1^) and 244 nm (34 686 cm^−1^ M^−1^), whereas, complex 7 absorbs light at 244 nm (50 825 cm^−1^ M^−1^) and 277(sh) nm (12 415 cm^−1^ M^−1^). A d → d transition is also observed at 655 nm (89 cm^−1^ M^−1^) for 6 and 882 nm (23 cm^−1^ M^−1^) for 7. The absorbance values of 6 are consistent with values reported for a similar complex, [Cu(tptz)Cl_2_]·2H_2_O. The bands at 204, 228, 267, and 298 nm have been assigned as π → π* transitions and a d → d band was observed at 763 nm; tptz = 2,4,6-tris(2-pyridyl)-1,3,5-triazine.^26^ The absorbance values of the nickel(ii) complex, 7, are in agreement with Abdi *et al.*,^27^ which show absorbance bands at, 204, 257, 293 nm (π → π* transitions) and at 669, 778, 923 nm (d → d transition) for the complex [Ni(tptz)(CH_3_OH)Cl_2_]. Likewise, ligand based π → π* transitions derived from complex 8 are observed at 231 (74 819 cm^−1^ M^−1^), 239 (59 468 cm^−1^ M^−1^), and 256(sh) nm (22 543 cm^−1^ M^−1^). The absorbance values of the zinc(ii) complex 8 are comparable with Xian Sun *et al.*^28^ whom reported absorbance values of the [Zn_2_(Bpz*eaT)_2_(HBTC)_2_](CH_3_OH)_3_ complex at 220 nm, corresponding to the π → π* transition, and two LMCT bands at 260 and 280 nm; Bpz*eaT = 2,4-bis(3,5-dimethyl-1*H*-pyrazol-1-yl)-6-diethylamino-1,3,5-triazine.

### Cyclic voltammetry

**Fig. 5:**
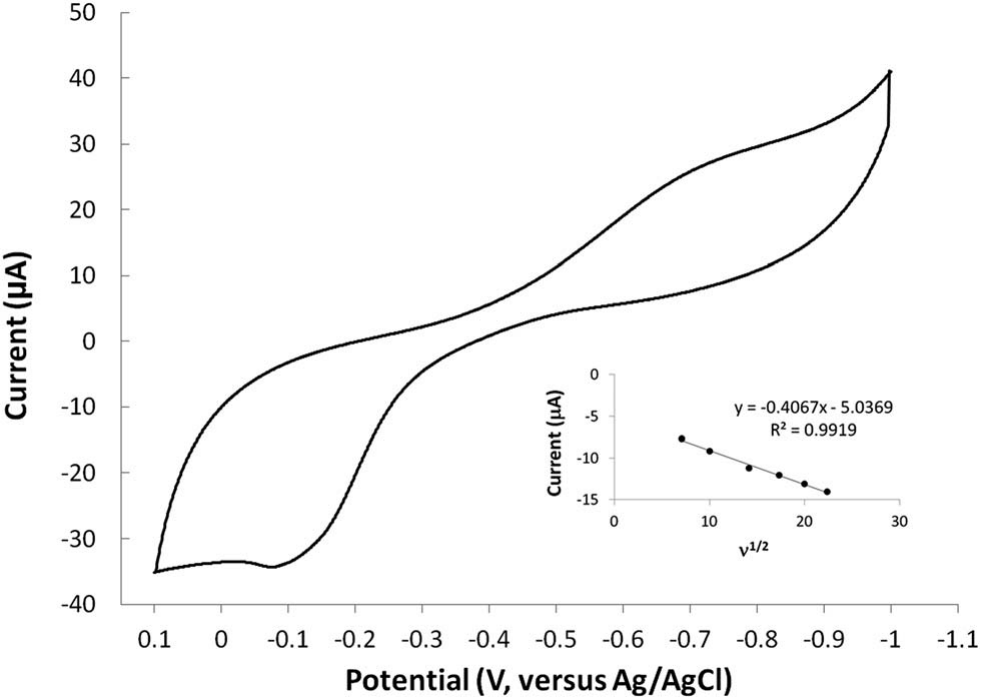
Cyclic voltammogram of complex 6 (3.0 mM) obtained in a 0.1 M KCl aqueous solution using a glassy carbon working electrode, platinum auxiliary electrode, and Ag/AgCl reference electrode at 500 mV s^−1^. The inset is a plot of *ν*^1/2^*versus I*_pa_ and indicates that the oxidation is diffusion controlled.

The redox behavior of complexes 6–8 were studied using cyclic voltammetry in aqueous solution. For comparison, cyclic voltammetry of molecule 1 was also carried out and no redox activity was observed in the window studied (1000 to −1000 mV). Therefore, any redox signals observed in the metal complexes is attributed to the metal-ion within the complex. The cyclic voltammogram of the copper(ii) complex (6) is shown in (Fig. 5). The Cu^I/II^ oxidation wave is observed at −108 mV at a scan rate of 500 mV s^−1^. The oxidation wave is not observed at scans in the anodic direction that begin at potentials more positive than −700 mV. This observation indicates that the ill-defined cathodic wave around −700 mV is coupled to the oxidation wave and is therefore assigned as a Cu^II/I^ event. The Cu^II/I^ redox couple is irreversible based on the separation of the reduction and oxidation events. The shape of the reduction wave may be, in part, attributed to the two types of copper sites observed in the solid state structures of 6. The separation of the redox waves increased with increasing scan rate, consistent with an irreversible couple. Furthermore, a plot of the *ν*^1/2^*versus I*_pa_ indicates that the oxidation is diffusion controlled (inset Fig. 5). Complexes 7 and 8 did not show redox activity in the window studied (1000 to −1000 mV). The lack of redox activity in complex 7 is somewhat surprising since octahedral nickel(ii) complexes are known to be redox active.^29^ The lack of redox response observed for complex 8 is consistent with the expected redox inert behaviour of diamagnetic zinc(ii) systems. Altogether, this data shows that molecule 1 readily accommodates the +2 oxidation state of all metal ions evaluated, and that the reduced copper(i) species can only be accessed at potentials more negative than approximately −700 mV. The cyclic voltammetry results demonstrate that molecule 1 inhibits the copper(ii) reduction process known to generate toxic reactive oxygen species *in vivo*.

### Computational models

Density functional theory (DFT) calculations help to interpret the ligand protonation states observed in molecules 1, 6, 7, and 8. Table 7 shows optimized geometries, relative energies, and C–O bond lengths of enamine, imine, and alcohol conformations. All three isomers are neutral (charge zero). Calculations use the Gaussian 09 electronic structure package, DFT with the B3LYP exchange-correlation functional and the 6-311++G(d,p) basis set, and a continuum model for water solvent. Coordinates of optimized geometries are included as ESI.†

**Table 7:** Relative energies and optimized structures derived from DFT computation for molecule 1

Entry	1	2	3
Structure evaluated	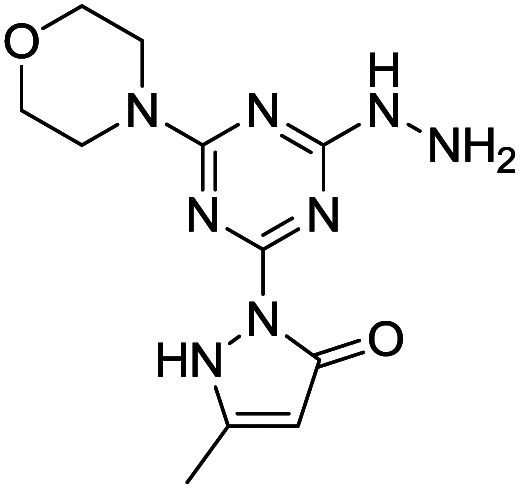	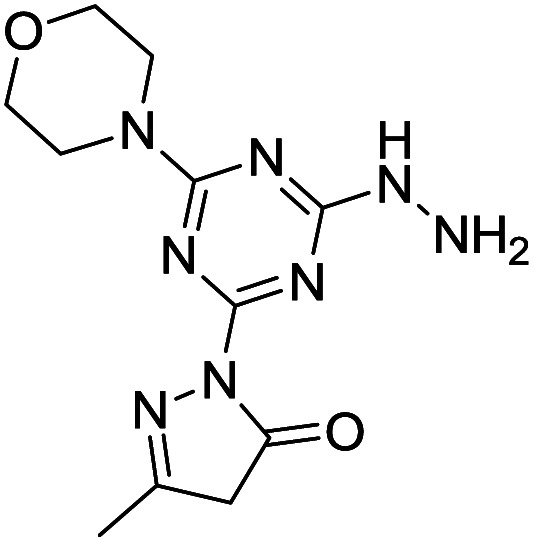	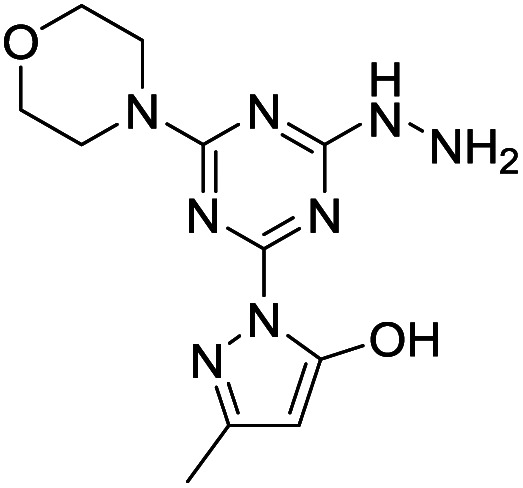
Optimized structure	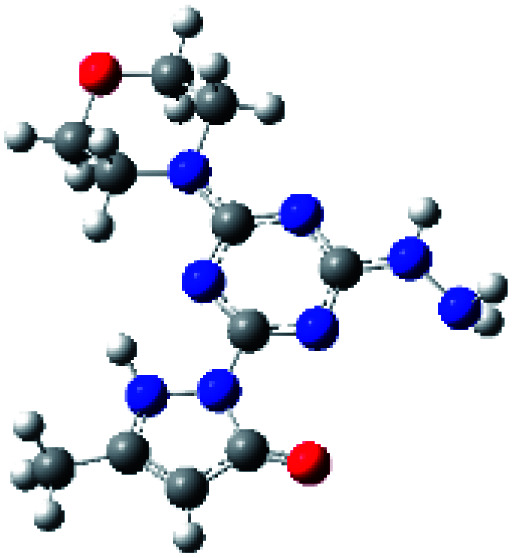	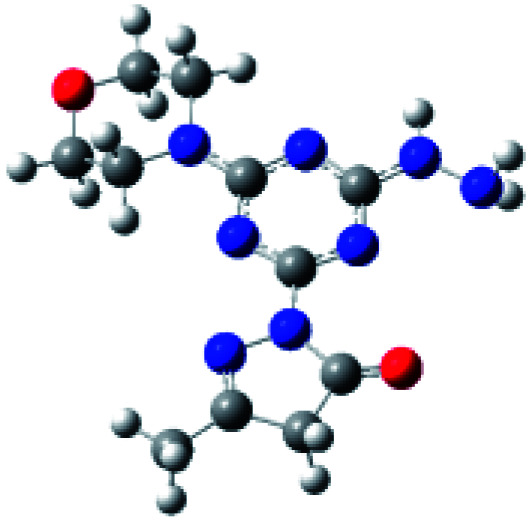	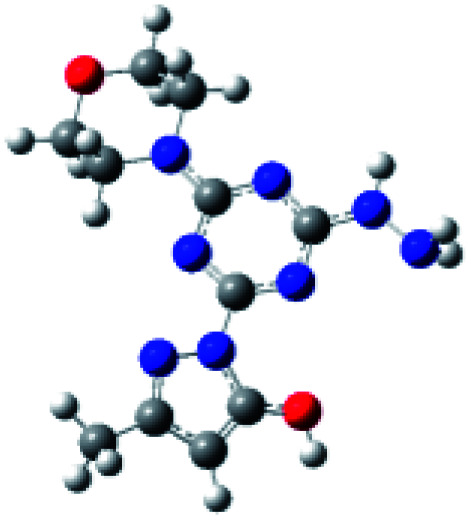
Relative energy (kcal mol^−1^)	0.00	+1.27	+7.594
C–O (Å)	1.23	1.21	1.34

The results in Table 7 show that enamine (entry 1) and imine (entry 2) structures are predicted to be most stable. This is consistent with the conversion between imine and enamine species observed in D_2_O. The computed enamine and imine C–O bond lengths are slightly longer than the experimental value for molecule 1, 1.27 Å (based on XRD of 6–8). The experimental values are mid-way between typical C=O and C–O bonds and are consistent with decreased C=O bonding due to ligand–metal interactions. The third alcohol structure is predicted to be relatively unstable, but is most consistent with the deprotonated ligand seen in 6. Overall, the simulations suggest that all three structures could be experimentally relevant, and that coordination to metal could stabilize entry 3.

### Antioxidant activity

**Fig. 6:**
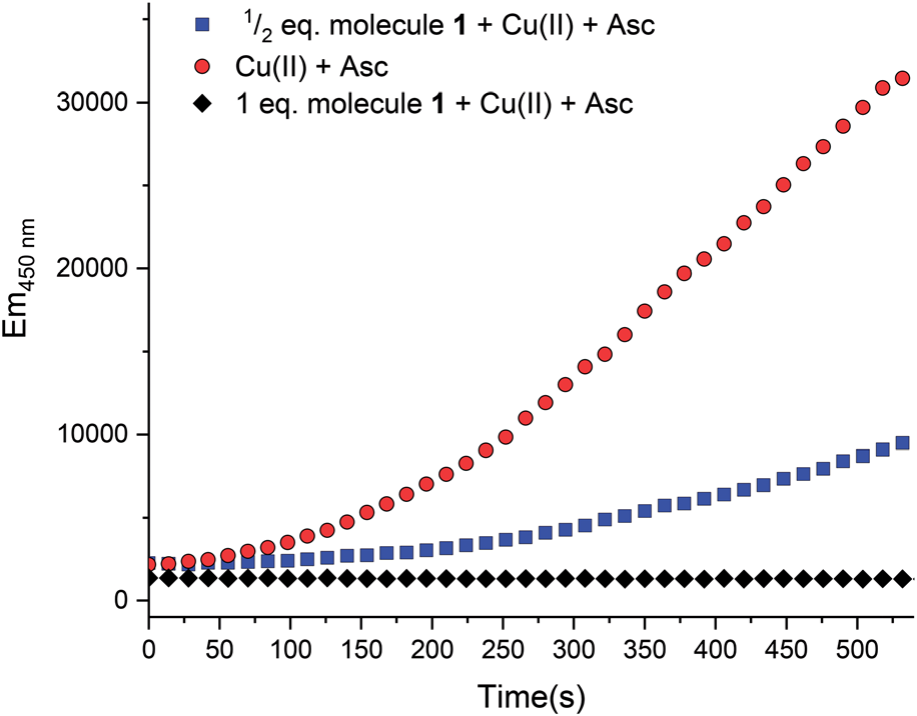
Fluorescence intensity of 7-hydroxy-CCA after incubation of CCA [500 μM] and ascorbate [300 μM] with Cu(ii) [10 μM] (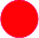). Compound 1 [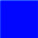, 5 μM; 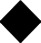, 10 μM] and Cu(ii) were combined prior to addition of ascorbate. No fluorescence was observed with CCA co-incubated with samples of: (1) ascorbate [300 μM] only or (2) Cu(ii) [40 μM]. All solutions except Cu(NO_3_)_2_ (Milli-Q water only) were dissolved and diluted in KH_2_PO_4_/NaCl [15 mM] buffer containing desferryl [2 μM]. Final volume = 3 mL (final concentrations listed; raw data available in ESI†).

In aerobic conditions and in the presence of ascorbate, copper can serve as a catalyst to produce hydroxyl radical ions.^20^ When unregulated metal ions facilitate this process *in vivo*, the increased levels of oxidative stress have been implicated in a range of diseases.^16,18,21^ Therefore, there is an increasing interest in finding ways to mitigate such detrimental pathways.^21,30,31^ The ability of molecule 1 to bind biologically relevant metal ions prompted further exploration of the function to halt the redox cycling of copper ions. The conversion of oxygen into hydroxyl radicals can be simulated *in situ*. As shown in Fig. 6 (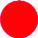), radical production can be quantified with coumarin carboxylic acid (CCA). This species stoichiometrically converts to the fluorescent hydroxy-CCA species in the presence of the hydroxyl radical generated by copper in the presence of ascorbate and oxygen. The process is halted by the addition of molecule 1, presumably by chelation of the copper. As shown in Fig. 6 [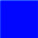], 1/2 equivalent of molecule 1 (*vs.* copper(ii)) results in a marked decrease in fluorescence. A full equivalent of molecule 1 (Fig. 6, [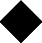]), completely inhibits the conversion of CCA to the fluorescent species. The copper(ii): molecule 1 interaction shifts the Cu^II/I^ potential outside the accessible range of reduction by ascorbate.^20,32^ This result is consistent with the cyclic voltammetry data showing very negative Cu^II/I^ redox potentials and the characterization of molecule 1 to bind copper(ii) ions. Altogether this assay shows that the metal binding reactivity of molecule 1 has potential as a therapeutic for metal ion misregulation.

### Cellular toxicity

**Fig. 7:**
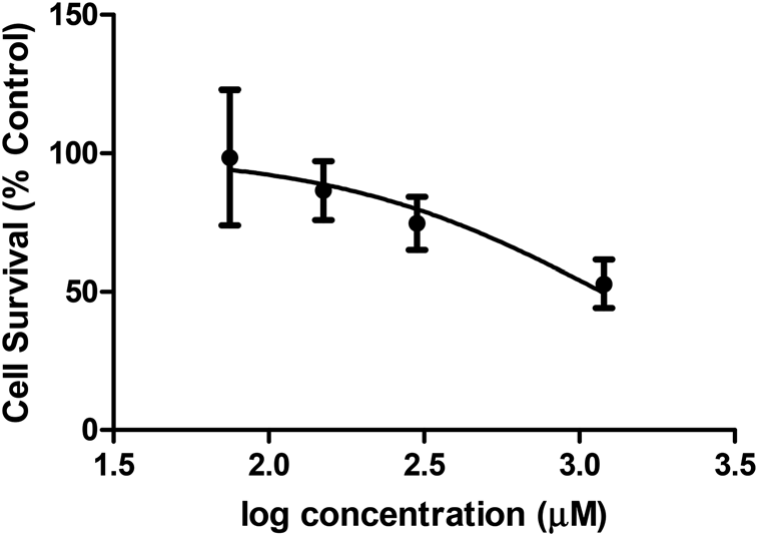
Cytotoxicity of molecule 1 in the HEK-293 cell line (*n* = 8) using the MTT assay.

A preliminary assessment of toxicity was carried out as there are an ever growing number of diseases known to arise from metal ion mis-regulation and molecule 1 shows the capacity to bind biologically relevant transition metal ions.^18,21,33–41^ The MTT assay [MTT = 3-(4,5-dimethyl-2-thiazolyl)-2,5-diphenyl-2*H*-tetrazolium bromide] was employed to evaluate the toxicity of molecule 1 in HEK-293 cells. Fig. 7 shows the results of the MTT assay, which reflect incubating cells for 16 h with molecule 1 and measuring cell survival with MTT. Concentrations above 75 μM of molecule 1 resulted in no observed toxic effects compared to cells with no exposure to molecule 1. A concentration dependent cell death response was observed at increased concentrations of molecule 1, with an EC_50_ = 1.183 ± 0.002 mM. Therapeutic windows are typically expected to be an order of 10^2^–10^3^ lower than EC_50_ toxicities. Therefore, future potential therapeutic applications of molecule 1 would focus on activities achievable within the μM range. Such studies are further encouraged by the capacity for molecule 1 to shift the redox couple of copper(ii) to more negative potential values, thus potentially avoiding redox chemistry known to produce toxic reactive oxygen species *in vivo*. Future studies would focus on the capacity of molecule 1 to prevent oxidative stress in biological conditions through the molecule's ability to bind to redox active transition metal ions.

## Conclusions

In this work, we have successfully prepared a triazine bearing a pyrazolone moiety and explored metalation with first row transition metals, copper(ii), nickel(ii), and zinc(ii). The solid-state structure of 6 shows that 1 serves as a tridentate ligand to a square pyramidal copper(ii) center and the remainder of the coordination sphere is completed by counter ions or solvent molecules. Unlike the copper complex, the coordination spheres of 7 and 8 are complete with two molecules of 1 in an octahedral geometry. Previous studies have shown that incorporating a pyrazole moiety into a triazine based ligand stabilizes the formation of a 5,5,5 ring system in which metal coordination is achieved *via* a nitrogen atom on the pyrazole. Herein, the ring size has been tuned by functionalizing the pyrazole with a keto group; the asymmetric molecule 1 engenders the formation of a very stable 6,5,5 ring system in which the newly added keto group binds the metal ion. Cyclic voltammetry studies show that the ligand and complexes 7 and 8 are redox inert, but studies of 6 show an irreversible Cu^II/I^ event. Cell toxicity data suggests that future work with these types of molecules could include investigating their potential as therapeutics for metal ion mis-regulation as the production of oxidative stress and disease. This represents the first report of this new type of ligand and its coordination chemistry. The results can be used to further explore novel triazine based ligands and their potential applications.

## Experimental section

### General procedure

All reagents and solvents were obtained from commercial sources and used as received, unless noted otherwise. Elemental analyses on were performed by Canadian Microanalytical Services Ltd. NMR spectra were recorded on a Bruker Avance III HD 400 MHz spectrometer. ^1^H NMR chemical shifts were referenced to CDCl_3_ (7.26 ppm), D_2_O (4.80 ppm), and DMSO-*d*_6_ (2.55 ppm). ^13^C NMR chemical shifts were referenced to CDCl_3_ (77.23 ppm), and DMSO-*d*_6_ (39.52 ppm). The following abbreviations were used to describe multiplets: s (singlet), t (triplet), m (multiplet), and br (broad). The following format was used to report peaks: chemical shift in ppm [multiplicity, coupling constant(s) in Hz, integral, and assignment]. Infrared (IR) spectra were recorded on a Jasco FT/IR-4600 and Bruker Alpha FT/IR in the 4000–400 cm^−1^ range. High-resolution mass spectra (HR-MS) were recorded on an Agilent Technologies 6224 TOF LC/MS system. UV-vis spectra were recorded in a 3 mL quartz cuvette with a path length of 1.0 cm on a 8453 Agilent spectrophotometer between 190 and 1100 nm. The crystalline materials used for XRD analysis were also used for elemental analysis and spectroscopic characterizations.

### 4-(4,6-Dichloro-1,3,5-triazin-2-yl)morpholine (2)

The procedure follows a known synthesis.^16^ To a stirred solution of cyanuric chloride (10.0 g, 54 mmol) in acetone (100 mL), a solution of morpholine (3.4 mL, 39 mmol) and triethylamine (5.3 mL, 39 mmol) in acetone (100 mL) was added dropwise at 0 °C for 1 h, then quenched with H_2_O, stirred for 5 minutes, filtered, washed with a cold 1 : 1 mixture of H_2_O/MeOH and dried under vacuum to yield 2 as a white powder (8.34 g, 91%). ^1^H NMR (CDCl_3_, 400 MHz): *δ* 3.83 (t, *J* = 4.8 Hz, 4H), 3.69 (t, *J* = 4.8 Hz, 4H). ^13^C NMR (CDCl_3_, 125 MHz): *δ* 170.3, 164.0, 66.3, 44.4. HR-MS (ESI) *m*/*z* calcd for C_7_H_9_N_4_Cl_2_ [M + H]^+^ 235.0153; found 235.0161.

### 4-(4-Chloro-6-BOC-hydrazine-1,3,5-triazin-2-yl)morpholine (3)

To a stirred solution of 2 (6 g, 25.4 mmol) in THF (100 mL) *tert*-butyl carbazate (5 g, 38 mmol) and *N*,*N*-diisopropylethylamine (8.8 mL, 51 mmol) was added. The reaction was stirred for 12 h at room temperature. The solvent was removed by rotary evaporation under reduced pressure and extracted with H_2_O (100 mL) and DCM (100 mL × 2). The organic layer was collected. Following concentration, the crude product was purified by flash column chromatography (hexanes: EtOAc 2 : 1) to afford compound 3 (7.20 g, 87%) as white solid. ^1^H NMR (CDCl_3_, 400 MHz): *δ* 3.85–3.74 (m, 4H), 3.70–3.63 (m, 4H), 1.44 (s, 9H). ^13^C NMR (CDCl_3_, 125 MHz): *δ* 169.3, 167.2, 164.4, 155.4, 81.6, 66.6, 44.0, 28.2. HR-MS (ESI) *m*/*z* calcd for C_12_H_20_N_4_O_3_Cl [M + H]^+^ 331.1285; found 331.1293.

### 4-(4-Hydrazine-6-BOC-hydrazine-1,3,5-triazin-2-yl)morpholine (4)

To a stirred solution of 3 (3 g, 9.2 mmol) in 1,4-dioxane (15 mL) was added hydrazine (1.4 mL, 46 mmol). The reaction was stirred in a CEM 300 W microwave under dynamic mode at 90 °C for 30 min. The solvent was removed by rotary evaporation under reduced pressure and filtered with H_2_O. Azeotropy with toluene (50 mL × 2) under reduced pressure by rotary evaporation afforded compound 4 (2.75 g, 93%) as white solid. ^1^H NMR (DMSO-d_6_, 400 MHz): *δ* 4.57 (br, 2H), 3.75–3.63 (m, 4H), 3.61–3.54 (m, 4H), 1.39 (s, 9H). ^13^C NMR (DMSO-d_6_, 125 MHz): *δ* 168.2, 167.8, 164.9, 156.4, 79.1, 66.5, 43.6, 28.6. HR-MS (ESI) *m*/*z* calcd for C_12_H_23_N_8_O_3_ [M + H]^+^ 327.1893; found 327.1971.

### 4-(4-Methylpyrazolone-6-BOC-hydrazine-1,3,5-triazin-2-yl)morpholine (5)

In a sealed tube, 4 (250 mg, 0.77 mmol) was dissolved in 95/5 THF/pyridine (10 mL) followed by the addition of Lawesson's reagent (310 mg, 0.77 mmol) and *N*-methylacetoacetamide (0.11 mL, 0.70 mmol). The reaction was stirred at 90 °C for 4 h. The solvent was removed by rotary evaporation under reduced pressure and extracted with H_2_O (50 mL) and DCM (50 mL × 2). The organic layer was collected and upon concentration, the crude product was purified by flash column chromatography (DCM: MeOH 9 : 1) to afford compound 5 (245 mg, 82%) as white solid. ^1^H NMR (DMSO-d_6_, 400 MHz): *δ* 5.54 (s, 1H), 3.80–3.59 (m, 8H), 2.24 (s, 3H). ^13^C NMR (DMSO-d_6_, 125 MHz): *δ* 165.8, 163.4, 162.0, 157.7, 156.1, 153.7, 88.7, 81.5, 66.4, 43.7, 28.2, 14.8. HR-MS (ESI) *m*/*z* calcd for C_16_H_25_N_8_O_4_ [M + H]^+^ 393.1999; found 393.2760.

### 4-(4-Methylpyrazolone-6-hydrazine HCl-1,3,5-triazin-2-yl)morpholine (1)

To a stirred solution of 5 (1 g, 2.5 mmol) in MeOH (10 mL) excess conc. HCl (5 mL) was added. The reaction was stirred for 12 h at room temperature. The solvent was removed under reduced pressure and triturated with DCM. Following tituration with DCM compound 1 was isolated in quantitative yields as white solid. Selected IR bands (cm^−1^): 1638 (C=O), 1562, 1574, 1431, 1380, 1301, 1269, 794. ^1^H NMR (DMSO-d_6_, 400 MHz): *δ* 5.54 (s, 1H), 3.80–3.56 (m, 8H), 2.24 (s, 3H). ^13^C NMR (DMSO-d_6_, 125 MHz): *δ* 164.2, 152.5, 90.6, 66.4, 44.4. ^1^H NMR (D_2_O, 400 MHz): *δ* 3.85–3.45 (m, 10H), 2.16 (s, 3H). ^13^C NMR (D_2_O, 125 MHz): *δ* 164.3, 163.3, 163.0, 162.1, 154.2, 90.6, 66.1, 44.2, 43.9, 11.6. HR-MS (ESI) *m*/*z* calcd for C_11_H_17_N_8_O_2_ [M + H]^+^ 293.1474; found 293.1957.

### [Cu(1)(Cl)_2_][Cu(1)(Cl)(H_2_O)] (6)

To a stirred solution of 1 (50 mg, 0.15 mmol) in EtOH (5 mL) was added CuCl_2_ (20.5 mg, 0.15 mmol) dissolved in EtOH (3 mL). The reaction was stirred for 12 h at room temperature, filtered, and washed with EtOH (20 mL) to afford compound 6 (31 mg, 48%) as a green powder. Single crystals suitable for XRD analysis were grown by the slow evaporation of H_2_O. Selected IR bands (cm^−1^): 3211, 3129, 1655 (C=O), 1638 (C=O), 1613, 1583, 1550, 1496, 1448, 1422, 1348, 1306, 1289, 1174, 1014, 784, 763, 620. HR-MS (ESI) *m*/*z* calcd for C_11_H_16_ClCuN_8_O_2_ [M + H]^+^ 390.0381; found 390.0937. HR-MS (ESI) *m*/*z* calcd for C_11_H_15_CuN_8_O_2_ [M + H]^+^ 354.0614; found 354.1092. Anal. calcd for C_22_H_31_Cl_3_Cu_2_N_16_O_5_·4H_2_O: C, 29.13; H, 4.56; N, 24.71. Found C, 29.20; H, 4.01; N, 24.34.

### [Ni(1)_2_][Cl]_2_ (7)

To a stirred solution of 1 (50 mg, 0.15 mmol) in EtOH (5 mL) was added NiCl_2_ (H_2_O)_6_ (18 mg, 0.075 mmol) dissolved in EtOH (3 mL). The reaction was stirred for 12 h at room temperature, filtered, and washed with EtOH (20 mL) to afford compound 7 (28 mg, 45%) as purple powder. Single crystals suitable for XRD analysis were grown by the slow evaporation of H_2_O. Selected IR bands (cm^−1^): 3130, 1636 (C=O), 1576, 1507, 1425, 1284, 1179, 1113, 1061, 1016, 856, 793, 762, 578. HR-MS (ESI) *m*/*z* calcd for C_22_H_31_N_16_NiO_4_ [M + H]^+^ 641.2068; found 641.3206. Anal. calcd. for C_22_H_32_Cl_2_NiN_16_O_4_·7HCl·5H_2_O: C, 24.94; H, 4.66; N, 21.15. Found C, 24.80; H, 4.73; N, 20.98.

### [Zn(1)_2_]Cl (8)

To a stirred solution of 1(50 mg, 0.15 mmol) in EtOH (5 mL) was added ZnCl_2_ (10 mg, 0.075 mmol) dissolved in EtOH (3 mL). The reaction was stirred for 12 h at room temperature, filtered, and washed with a 3 : 1 mixture of acetone: EtOH (20 mL) to afford compound 8 as a white powder (37 mg, 57%). Single crystals suitable for XRD analysis were grown by the slow evaporation of H_2_O. *δ* 9.95 (s, 1H), 5.31 (s, 1H), 3.80–3.59 (m, 8H), 2.27 (s, 3H). Selected IR bands (cm^−1^): 3180, 3132, 1645 (C=O), 1612, 1581, 1556, 1513, 1440, 1430, 1406, 1345, 1300, 1287, 1179, 1098, 1061, 1012, 791, 625.^1^H NMR (DMSO-d_6_, 400 MHz): *δ* 5.32 (s, 1H), 3.80–3.60 (m, 8H), 2.27 (s, 3H). HR-MS (ESI) *m*/*z* calcd for C_22_H_31_N_16_ZnO_4_ [M + H]^+^ 647.2006; found 647.3107, calcd for C_11_H_16_ClN_8_ZnO_2_ [M + H]^+^ 391.0376; found 391.0919. Anal. calcd for C_26_H_31_Cl ZnN_16_O_4_·4HCl·5EtOH: C, 36.24; H, 6.18; N, 21.13. Found C, 36.40; H, 5.79; N, 21.43.

### X-ray data collection and structure solutions

The single crystals of 6, 7 and 8 were grown from aqueous solution by slow evaporation at room temperature. The single crystal of each compound, from available crystals, was mounted on the goniometer using Paratone-N oil (cryoprotectant) on the tip of MiTeGen MicroLoops LD™, 45 mm away from the detector and was cooled to a temperature of 100(1) K under the flow of liquid nitrogen using Oxford Cryosystem.^42^ X-ray intensity data were collected on a Bruker D8 Quest diffractometer equipped with a Photon 100 CMOS detector and generator operating at 50 kV and 30 A. For each crystal, an exposure time of 20 seconds was used with a scan angle of 0.5° per frame. The indexing of Bragg intensities was carried out with APEX3 package.^43^ Data reduction and absorption corrections were performed with the SAINT^44^ and SADABS^45^ software packages, respectively. Structures were solved by the direct method using the SHELXL-97 (ref. 46) software and refined using SHELXL in the WinGX package.^47^ The atomic displacement parameters of hydrogen atoms were refined isotropically and non-hydrogen atoms were anisotropically refined using the full-matrix least-squares method on F squared. Hydrogen atoms were located from the difference-Fourier analysis. The H atoms attached to carbon were fixed using a riding model, whereas those attached to heteroatoms were freely refined. The asymmetric units of 6, 7 and 8 contains multiple disorders. During the refinement of all disorders, the sum of the occupancies of the two disordered parts was fixed to unity. The O8 atom associated with disorder water molecule in 6, flipped disordered over two sites, and the occupancy ratio refined to 0.679 : 0.320. In order to avoid correlation of the thermal parameters, the atomic displacement parameters (ADPs) of both parts were constrained to be equal. Due to limited quantity of diffraction data, the positions of two hydrogen atoms bond to O10 were not ascertained. The disorder of the morpholine ring in 7, was observed as residual electron density oriented in approximately a mirror to the major occupancy components. All disordered atoms in 7 including one Cl^−^ counter ion and one water molecule were refined using aforementioned ADP constraint except O6A, O6B, C19A, C19B, C21A and C21B which were refined isotropically. The occupancies of the two components were refined, yielding an approximately 0.538 : 0.462 ratio. The similar procedure was adopted to model the disordered water molecules in 8. The water molecule associated with O5 in 8 is working as a bridge between Cl^−^ counter ion and N16 of the hydrazine ring through hydrogen bonding. Owning to the importance of this long range interaction in crystal packing, O5–H5B distance and H5A-O5-H5B angle were restrained to 0.982 Å and 104.414°, respectively. The disordered water molecules were refined using equal ADPs constraints and the occupancy ratio of the disordered parts was refined to 0.496 : 0.503. The details of data collection, and statistics of structure refinement are summarized Table S1.†

For each modelled structure, larger than desired residual electron density peaks remain after refinement of the data, which is typical for this class of compounds.^48,49^ The origin of these densities can be attributed to various factors,^49^ but in the present structures, it can be attributed to the additional disorders especially in 8. Initially, two occupancies were refined freely to identify possible site pairings, but the modelling of multiple occupancies of these disorders was not completely possible with available data. These disorders and residual densities are associated with outer-sphere, non-coordinating chloride counter ions and water molecules only, which ensures the structural accuracy of main coordination moiety.

### Cyclic voltammetry

A Basi C3 cell stand with a glassy carbon working electrode, Ag/AgCl reference electrode, and platinum wire auxiliary electrode were used to conduct the electrochemical analysis of all compounds. A standard three electrode cell under a blanket of N_2_ at room temperature was used to obtain all voltammograms. All experiments were carried out in aqueous solution with 0.1 M KCl as the supporting electrolyte and were referenced to Ag/AgCl.

### Computations

DFT calculations were performed using a hybrid functional (the three-parameter exchange functional of Becke (B3)^50^ and the correlation functional of Lee, Yang, and Parr (LYP)37)^51^ (B3LYP) as implemented in Gaussian 09.^52^ For each calculation, all atoms were optimized *via* the use of the 6-311g(d,p) basis set with polarization. A frequency calculation was performed alongside each geometry optimization to ensure the stability of the ground state as ascertained by the absence of imaginary frequencies. Coordinates for each structure were derived from XRD data in complexes 6–8. Optimized structure figures were generated by use of the GaussView program.^53^

### Coumarin carboxylic acid assay

#### Ascorbate Studies

All solutions were prepared in KH_2_PO_4_/NaCl [1.5 mM] buffer containing desferryl [1 μM].^20,32^ except Cu(NO_3_)_2_, which was dissolved and diluted in Milli-Q water. Final sample volume = 3 mL. Each experiment was performed in triplicate. Hydroxyl radical production was followed measuring the conversion of CCA into 7-hydroxy-CCA (*λ*_ex_ = 395 nm, *λ*_em_ = 450 nm) nm. General order of addition: CCA [500μM], molecule 1 (1/2 eq. = 20 μM; 1 eq. = 40 μM), or copper [40 μM], then ascorbate [300μM].

### Cell culture and cytotoxicity assays

Human Embryonic Kidney cells (293HEK) cells (ATCC) were grown in Dulbecco's Modified Eagle's Medium-high glucose (DMEM) supplemented in 10% fetal bovine serum (Sigma) and penicillin (10μ mL^−1^)/streptomycin (0.1 mg mL^−1^) (Sigma), 2 mM glutamine (Sigma) and MEM non-essential amino acids (1×) (Sigma), at 37C, 5% CO2, 95% air.

Cytotoxicity studies were carried out using the MTT assay. Briefly, cells were plated at a density of 5000 cells per well in a 96 well tray. Following an overnight incubation, the cells were treated with the indicated concentrations of drug and further incubated for 16 hours under normal growing conditions. Following this, the medium and drug were removed and 100 μL per well of MTT (Thiazolyl Blue Tetrazolium Bromide, Sigma) was added at a concentration of 1 mg mL^−1^ in serum-free DMEM. Cells were incubated in this solution for 4 hours under normal growing conditions. Next, the MTT solution was removed and the precipitate generated was solubilized in 100 μL of 100% DMSO for 5 min, RT, whilst shaking. The absorbance was measured at 540 nm in an Omega FLUOstar microplate reader (BMG Labtech). Results are presented as the average of 8 replicates per concentration of drug. GraphPad Prism was used to calculate EC_50_ values.

## Author contributions

The manuscript was written through contributions of all authors. AY performed the synthesis and coordinated data collection. AM performed the crystallographic analysis. SB performed the titrations for metalation. GA performed cellular assays. KG and MB performed CCA assays. The work was supervised by EES and KNG. All authors have given approval to the final version of the manuscript.

## Conflicts of interest

There are no conflicts of interest to declare.

## Supplementary Material

RA-008-C7RA09459K-s001

RA-008-C7RA09459K-s002

RA-008-C7RA09459K-s003

RA-008-C7RA09459K-s004

RA-008-C7RA09459K-s005

## References

[cit1] Tappe H. , Helmling W., Mischke P., Rebsamen K., Reiher U., Russ W., Schläfer L. and Vermehren P., in Ullmann's Encyclopedia of Industrial Chemistry, Wiley-VCH Verlag GmbH & Co. KGaA, 2000, 10.1002/14356007.a22_651

[cit2] Kim J. Y. H., Lee J. W., Lee W. S., Ha H.-H., Vendrell M., Bork J. T., Lee Y., Chang Y.-T. (2012). ACS Comb. Sci..

[cit3] Lim J., Simanek E. E. (2005). Mol. Pharm..

[cit4] Sun D., Melman G., LeTourneau N. J., Hays A. M., Melman A. (2010). Bioorg. Med. Chem. Lett..

[cit5] Duan R.-r., Ou Z.-b., Wang W., Chen S., Zhou X.-h. (2015). Spectrochim. Acta, Part A.

[cit6] Simanek E. E., Abdou H., Lalwani S., Lim J., Mintzer M., Venditto V. J., Vittur B. (2010). Proc. R. Soc. A.

[cit7] Enciso A. E., Abid Z. M., Simanek E. E. (2014). Polym. Chem..

[cit8] Lim J., Mintzer M. A., Perez L. M., Simanek E. E. (2010). Org. Lett..

[cit9] Umali A. P., Crampton H. L., Simanek E. E. (2007). J. Org. Chem..

[cit10] Lee C., Ji K., Simanek E. (2016). Molecules.

[cit11] Ji K., Lee C., Janesko B. G., Simanek E. E. (2015). Mol. Pharm..

[cit12] Hanninen O. O. P. and Atalay M., Physiology and Maintenance - Volume II: Enzymes: The Biological Catalysts of Life, Nutrition and Digestion, 2009

[cit13] Melman G., Vimal P., Melman A. (2009). Inorg. Chem..

[cit14] Ekeltchik I., Gun J., Lev O., Shelkov R., Melman A. (2006). Dalton Trans..

[cit15] Gun J., Ekeltchik I., Lev O., Shelkov R., Melman A. (2005). Chem. Commun..

[cit16] Sastre M., Ritchie C. W., Hajji N. (2015). JSM Alzheimer's Dis. Relat. Dement.

[cit17] Xu J., Begley P., Church S. J., Patassini S., McHarg S., Kureishy N., Hollywood K. A., Waldvogel H. J., Liu H., Zhang S., Lin W., Herholz K., Turner C., Synek B. J., Curtis M. A., Rivers-Auty J., Lawrence C. B., Kellett K. A. B., Hooper N. M., Vardy E. R. L. C., Wu D., Unwin R. D., Faull R. L. M., Dowsey A. W., Cooper G. J. S. (2016). Sci. Rep..

[cit18] Bush A. I. (2013). J Alzheimers Dis..

[cit19] Cardoso B. R., Hare D. J., Lind M., McLean C. A., Volitakis I., Laws S. M., Masters C. L., Bush A. I., Roberts B. R. (2017). ACS Chem. Neurosci..

[cit20] Guilloreau L., Combalbert S., Sournia-Saquet A., Mazarguil H., Faller P. (2007). ChemBioChem.

[cit21] Niedzielska E., Smaga I., Gawlik M., Moniczewski A., Stankowicz P., Pera J., Filip M. (2016). Mol. Neurobiol..

[cit22] Kim T. S., Pae C. U., Yoon S. J., Jang W. Y., Lee N. J., Kim J. J., Lee S. J., Lee C., Paik I. H., Lee C. U. (2006). Int. J. Geriatr. Psychopharmacol..

[cit23] Pinson J.-A., Zheng Z., Miller M. S., Chalmers D. K., Jennings I. G., Thompson P. E. (2013). ACS Med. Chem. Lett..

[cit24] Dodd D. S., Martinez R. L. (2004). Tetrahedron Lett..

[cit25] Addison A. W., Rao T. N., Reedijk J., Vanrijn J., Verschoor G. C. (1984). J. Chem. Soc., Dalton Trans..

[cit26] Abdi K., Hadadzadeh H., Weil M., Rudbari H. A. (2014). Inorg. Chim. Acta.

[cit27] Hadadzadeh H., Maghami M., Simpson J., Khalaji A. D., Abdi K. (2012). J. Chem. Crystallogr..

[cit28] Wang Z. N., Wang X., Yue Wei S., Xiao Wang J., Ying Bai F., Heng Xing Y., Xian Sun L. (2015). New J. Chem..

[cit29] Harding P., Harding D. J., Phonsri W., Saithong S., Phetmung H. (2009). Inorg. Chim. Acta.

[cit30] Tramutola A., Lanzillotta C., Perluigi M., Butterfield D. A. (2016). Brain Res. Bull..

[cit31] Yan M. H., Wang X., Zhu X. (2013). Free Radical Biol. Med..

[cit32] Hureau C., Faller P. (2009). Biochimie.

[cit33] Roberts B. R., Ryan T. M., Bush A. I., Masters C. L., Duce J. A. (2012). J. Neurochem..

[cit34] McColl G., James S. A., Mayo S., Howard D. L., Ryan C. G., Kirkham R., Moorhead G. F., Paterson D., de Jonge M. D., Bush A. I. (2012). PLoS One.

[cit35] Sensi S. L., Paoletti P., Koh J.-Y., Aizenman E., Bush A. I., Hershfinkel M. (2011). J. Neurosci..

[cit36] Mok S. S., Bush A. I. (2010). Oxid. Stress Dis..

[cit37] Zhao Y., Zhao B. (2013). Oxid. Med. Cell. Longevity.

[cit38] Dasuri K., Zhang L., Keller J. N. (2013). Free Radical Biol. Med..

[cit39] Kim Y.-S., Joh T. H. (2012). Biomol. Ther..

[cit40] Bush A. I. (2011). Front. Neurodegener..

[cit41] Perez L. R., Franz K. J. (2010). Dalton Trans..

[cit42] Cosier J., Glazer A. M. (1986). J. Appl. Crystallogr..

[cit43] APEX3 APEX3 , Version Ver. 2016.9-0, Bruker-AXS, Madison, Wisconsin, USA, 2016

[cit44] Software packages SMART and SAINT, Siemens Analytical X-ray Instrument Inc., Madison, WI, 1996

[cit45] SADABS: Area-Detector Absorption Correction, Siemens Industrial Automation, Inc., Madison, WI, 1996

[cit46] Sheldrick G. , SHELX-97, Program for the refinement of crystal structure, University of Göttingen, Germany, 1997

[cit47] Farrugia L. (2012). J. Appl. Crystallogr..

[cit48] Bridgman E. C., Doherty M. M., Ellis K. A., Homer E. A., Lashbrook T. N., Mraz M. E., Pernesky G. C., Vreeke E. M., Oshin K. D., Oliver A. G. (2016). Acta Crystallogr., Sect. E: Crystallogr. Commun..

[cit49] Travis J. R., Zeller M., Zaleski C. M. (2015). Acta Crystallogr., Sect. E: Crystallogr. Commun..

[cit50] Becke A. D. (1993). J. Chem. Phys..

[cit51] Lee C. T., Yang W. T., Parr R. G. (1988). Phys. Rev. B.

[cit52] Frisch M. J. , Trucks G. W., Schlegel H. B., Scuseria G. E., Robb M. A., Cheeseman J. R., Scalmani G., Barone V., Mennucci B., Petersson G. A., Nakatsuji H., Caricato M., Li X., Hratchian H. P., Izmaylov A. F., Bloino J., Zheng G., Sonnenberg J. L., Hada M., Ehara M., Toyota K., Fukuda R., Hasegawa J., Ishida M., Nakajima T., Honda Y., Kitao O., Nakai H., Vreven T., Montgomery Jr J. A., Peralta J. E., Ogliaro F., Bearpark M. J., Heyd J., Brothers E. N., Kudin K. N., Staroverov V. N., Kobayashi R., Normand J., Raghavachari K., Rendell A. P., Burant J. C., Iyengar S. S., Tomasi J., Cossi M., Rega N., Millam N. J., Klene M., Knox J. E., Cross J. B., Bakken V., Adamo C., Jaramillo J., Gomperts R., Stratmann R. E., Yazyev O., Austin A. J., Cammi R., Pomelli C., Ochterski J. W., Martin R. L., Morokuma K., Zakrzewski V. G., Voth G. A., Salvador P., Dannenberg J. J., Dapprich S., Daniels A. D., Farkas Ö., Foresman J. B., Ortiz J. V., Cioslowski J. and Fox D. J., Gaussian, Inc., 2009

[cit53] Dennington R. , Keith T. A. and Millam J. M., GaussView, Version 6, Semichem Inc., Shawnee Mission, KS, 2016

