# Supplementary material for: A new triazine bearing a pyrazolone group capable of copper, nickel, and zinc chelation
Source: RSC Adv. 2018 Jan 16;8(6):3024–35. doi: 10.1039/c7ra09459k (PMC5831559; doi:10.1039/c7ra09459k)
Supplement: RA-008-C7RA09459K-s004 [file RA-008-C7RA09459K-s004.pdf]

Electronic Supplementary Material (ESI) for RSC Advances.
This journal is © The Royal Society of Chemistry 2018

SUPPORTING INFORMATION

A new triazine bearing a pyralozone group capable of copper, nickel, and zinc chelation

Akop Yepremyan[a], Arshad Mehmood[a], Samantha Brewer[a], Madalyn M. Barnett[a], Benjamin G. Janesko[a], Giridhar Akkaraju[b], Eric E. Simanek[a]*, and Kayla N. Green[a]*

Department of Chemistry and Biochemistry, Texas Christian University, Fort Worth, Texas 76129

Table of Contents

Table S1. Crystal data collection and structure refinement statistics 3

Figure S1. ^{1}H NMR spectrum of molecule **2** in $CDCl_3$ 4

Figure S2. ^{13}C NMR spectrum of molecule **2** in $CDCl_3$ 5

Figure S3. HR-MS spectrum of molecule **2** 6

Figure S4. ^{1}H NMR spectrum of molecule **3** in $CDCl_3$. 7

Figure S5. ^{13}C NMR spectrum of molecule **3** in $CDCl_3$ 8

Figure S6. HR-MS spectrum of molecule **3** 9

Figure S7. ^{1}H NMR spectrum of molecule **4** in $DMSO_{d-6}$. 10

Figure S8. ^{13}C NMR spectrum of molecule **4** in $DMSO_{d-6}$ 11

Figure S9. HR-MS spectrum of molecule **4** 12

Figure S10. ^{1}H, spectrum of molecule **5** in $CDCl_3$. 13

Figure S11. ^{13}C NMR spectrum of molecule **5** in $CDCl_3$ 14

Figure S12. HR-MS spectrum of molecule **5** 15

Figure S13. ^{1}H NMR spectrum of molecule **1** in $DMSO_{d-6}$. 16

Figure S14. ^{1}H NMR spectrum of molecule **1** in D_2O. 17

Figure S15. ^{13}C NMR spectrum of molecule **1** in $DMSO_{d-6}$ 18

Figure S16. ^{13}C NMR spectrum of molecule **1** in D_2O 19

Figure S17. HSQC spectrum of molecule **1** in D_2O........ 20

Figure S18. ^{1}H NMR spectrum of molecule **1** in D_2O after 18 h........ 21

Figure S19. ^{13}C NMR spectrum of molecule **1** in D_2O after 10 h........ 22

Figure S20. HR-MS spectrum of molecule **1**..23

Figure S21. IR spectrum of molecule **1** in D_2O..24

Figure S22. HR-MS spectrum of molecule **6**..25

Figure S23. Molecular structure of molecule **6**..26

Figure S24. IR spectrum of molecule **6**..27

Figure S25. HR-MS spectrum of molecule **7**..28

Figure S26. Molecular structure of molecule **7**..29

Figure S27. IR spectrum of molecule **7**..30

Figure S28. ^{1}H NMR spectrum of molecule **8** in $DMSO_{d-6}$...31

Figure S29. HR-MS spectrum of molecule **8**..32

Figure S30. Molecular structure of molecule **8**..33

Figure S31. IR spectrum of molecule **8**..34

Figure S32. Cyclic voltammogram of complex **6**..35

Figure S33. Fluorescence measured for CCA [500 µM] and ascorbate [300 µM] with Cu(II) [10 µM]..35

Figure S34. Fluorescence measured for CCA [500 µM] and ascorbate [300 µM] with Cu(II) [10 µM] and molecule **1** [10 µM]..36

Figure S35. Fluorescence measured for CCA [500 µM] and ascorbate [300 µM] with Cu(II) [10 µM] and molecule **1** [10 µM]..36

Figure S34. Fluorescence measured for CCA [500 µM] and ascorbate [300 µM] with Cu(II) [10 µM] and molecule **1** [10 µM]..36

Table S2. Geometric parameters *i.e.* bond lengths and angles (Å, º) for **6**..37

Table S3. Geometric parameters *i.e.* bond lengths and angles (Å, º) for **7**..41

Table S4. Geometric parameters *i.e.* bond lengths and angles (Å, º) for **8**..45

τ-value calculation*

The τ-value was calculated according to the formula defined by Addison [τ = (β - α)/60], where α ° = N(4)-M-Cl(1) and β ° = N(1)-M-N(3) are the angles that define the basal plane. Please note that for a perfect square-pyramidal species τ = 0.00, and for a perfect trigonal bi-pyramidal species, τ = 1.00.

For Cu1 complex

N4-Cu1-Cl1 = 170.31°

N6-Cu1-O2 = 166.42°

τ = (170.31° – 166.42°)/60 = 0.06°

For Cu2 complex

(a) N11-Cu2-Cl2 = 161.34°

(b) N14-Cu2-O5 = 166.34°

τ = (166.34° – 161.34°)/60 = 0.08°

*Based on: Addison, A. W.; Rao, N. T. *J. Chem. Soc. Dalton Trans.* **1984**, 1349-1356.

Table S1. Crystal data collection and structure refinement statistics for **6, 7** and **8.**

	6	7	8
Chemical formula	$C_{22}H_{39}Cl_3Cu_2N_{16}O_9$	$C_{22}H_{32}Cl_2N_{16}NiO_7$	$C_{22}H_{33}ClN_{16}O_{13}Zn$
Mr	905.14	762.23	830.48
Crystal System, Space group	Triclinic, P-1	Triclinic, P-1	Monoclinic, $P2_1/c$
Temperature (K)	100	100	100
a, b, c (Å)	11.3979(6), 12.0551(7), 13.6836(7)	12.261(3), 12.784(4), 13.598(4)	12.4036(8), 19.8086(13), 14.3748(9)
α β γ(°)	97.586 (2), 103.997 (2), 106.156 (2)	74.133(8), 70.006(8), 64.470(7)	90, 91.559(2), 90
D_{calc} (g cm^{-3})	1.756	1.416	1.562
F(000)	928	788	1712
Volume (Å^3)	1711.75 (16)	1787.3(9)	3530.6(4)
Z	2	2	4
Radiation type	Mo Kα(λ= 0.71073 Å)	Mo Kα(λ= 0.71073 Å)	Mo Kα(λ= 0.71073 Å)
Crystal shape & Color	Needle, blue	Needle, colorless	Block, colorless
Crystal size (mm)	0.23 × 0.07 × 0.05	0.29 × 0.16 × 0.08	0.33 × 0.12 × 0.11
μ (mm^{-1})	1.55	0.75	0.86
T_{min}, T_{max}	0.876, 0.933	0.868, 0.944	0.881, 0.914
θ (°)	3.0–27.18	3.1–26.9	3.0–26.4
# measured, independent	86344, 7595	68001, 8066	123887, 7840
Completeness (%)	99.7	100.0	100.0
R_{int} (%)	0.060	0.141	0.107
GOF on F^2	1.046	1.099	1.193
R_1 [I > 2sigma(I)]/ R_1 (all)	0.039/0.056	0.122/ 0.197	0.086/ 0.125
wR_2 [I > 2sigma(I)]/ wR_2 (all)	0.082/0.089	0.3076/0.358	0.1627 /0.176
$θ_{max}$/ $θ_{min}$ (e/Å^3)	2.20/ −1.00	2.18/ −1.20	1.22/ −1.06

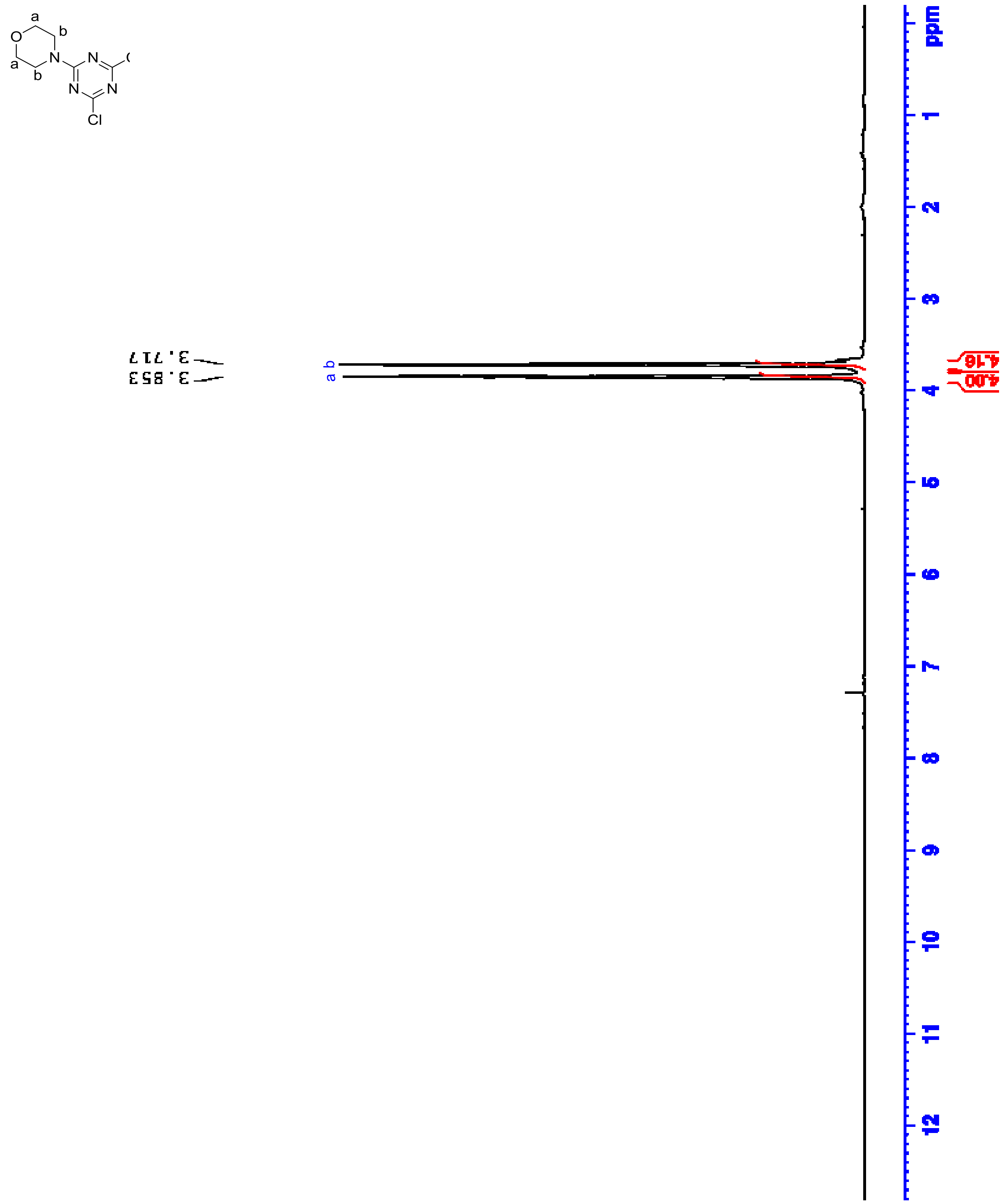

Figure S1. ^{1}H NMR spectrum of molecule **2** in $CDCl_3$.

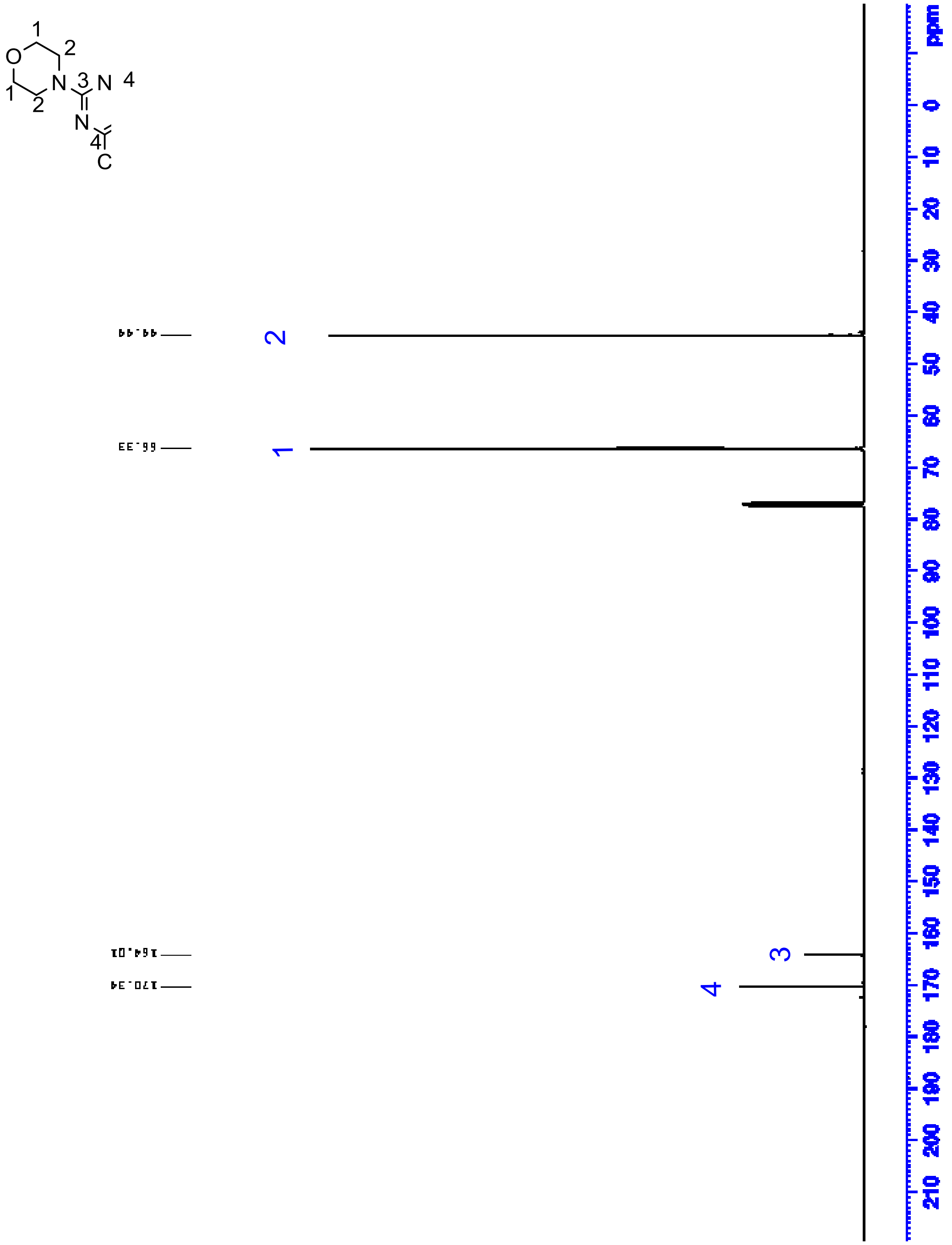

Figure S2. ^{13}C NMR spectrum of molecule **2** in $CDCl_3$.

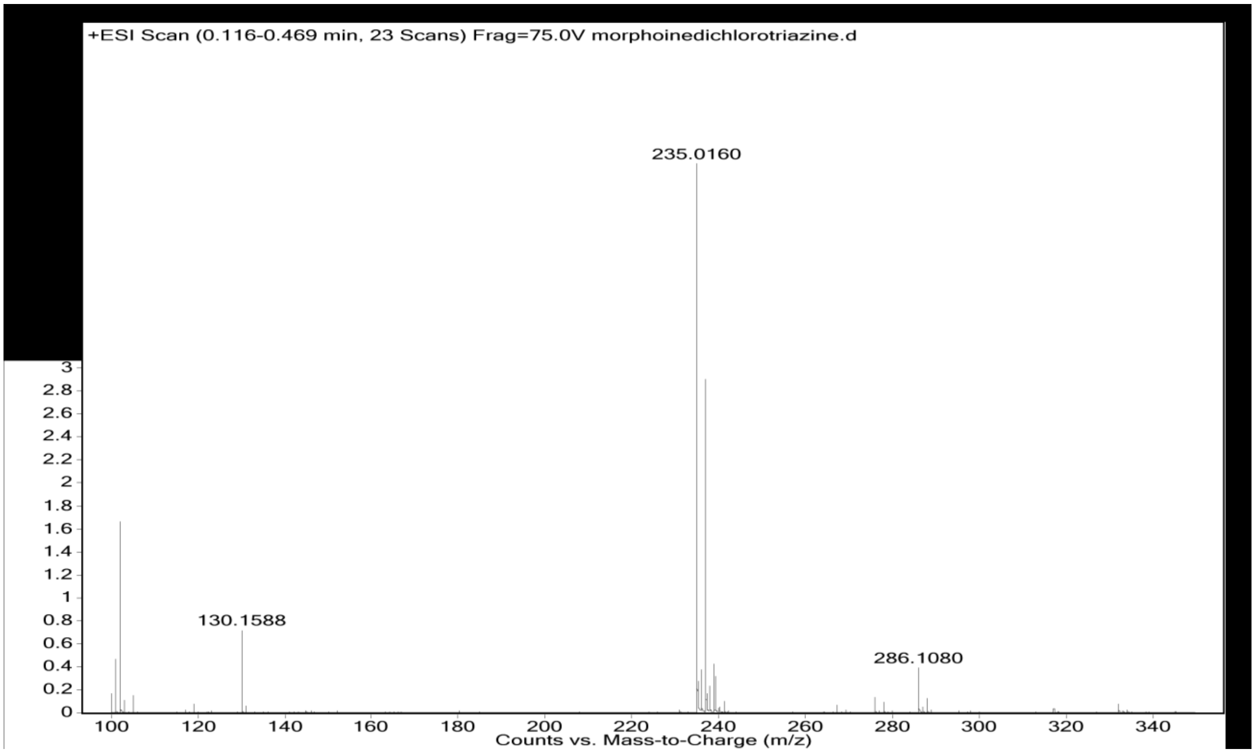

Figure S3. HR-MS spectrum of molecule **2**.

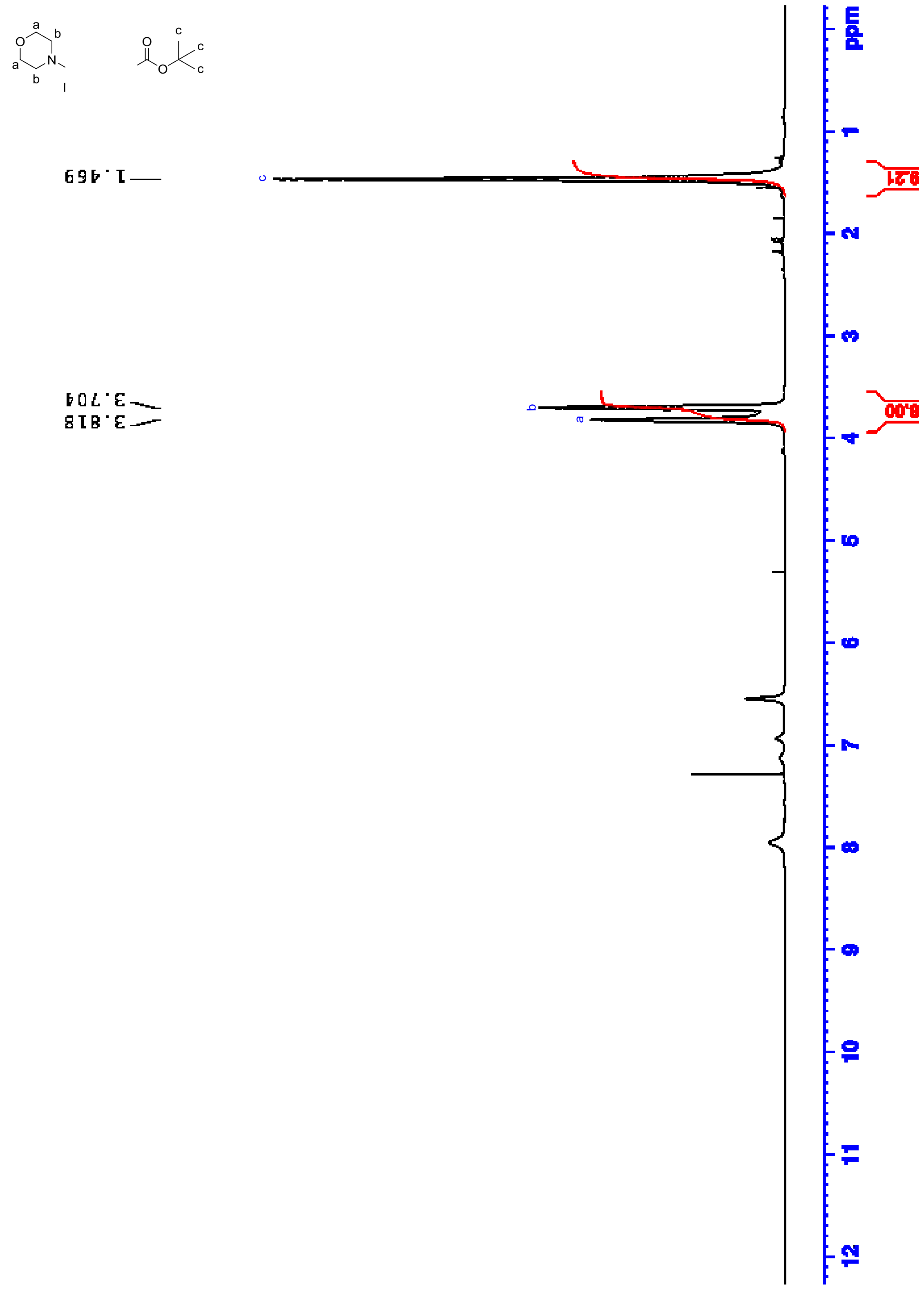

Figure S4. ^{1}H NMR spectrum of molecule **3** in $CDCl_3$.

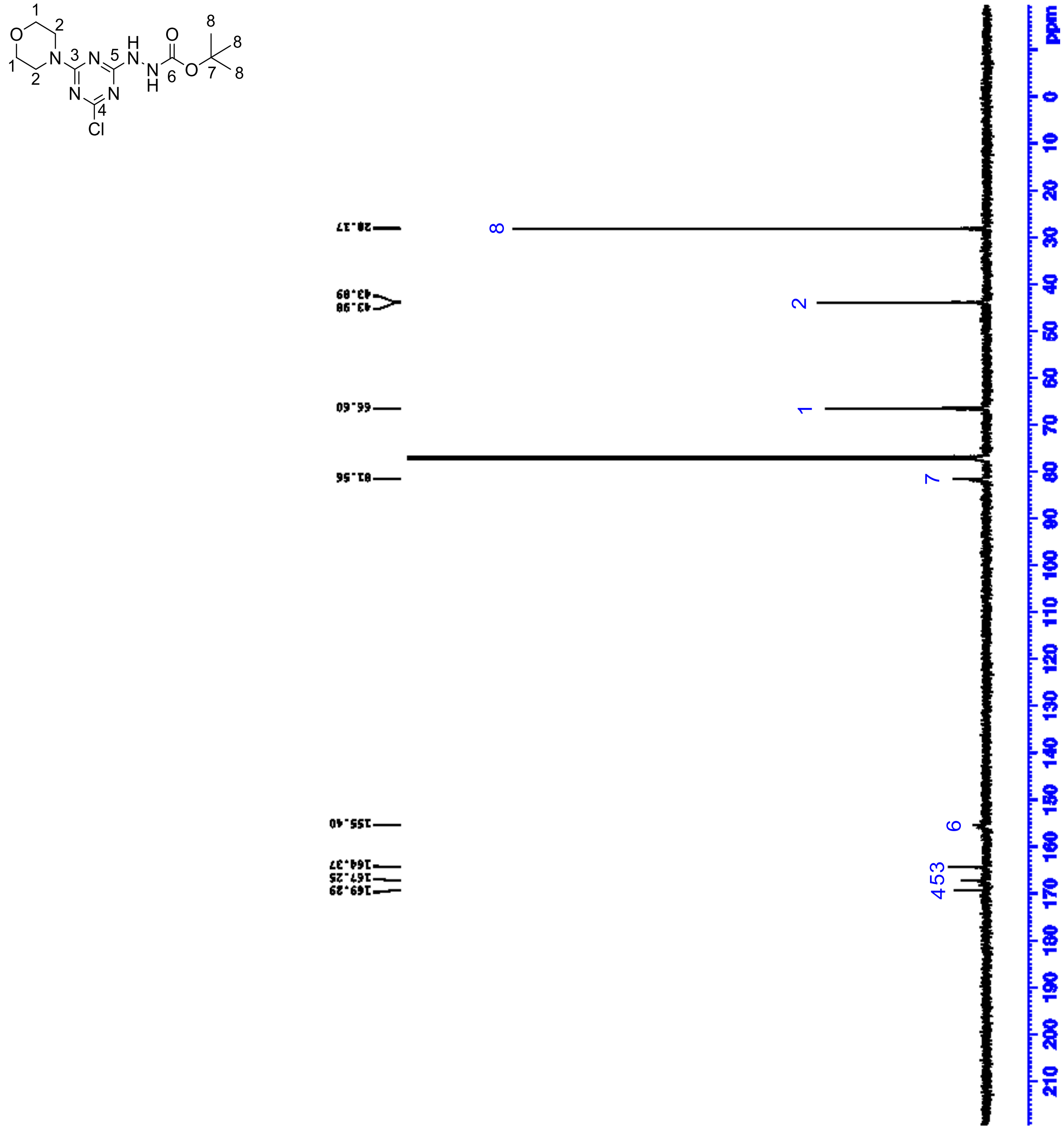

Figure S5. ^{13}C NMR spectrum of molecule **3** in $CDCl_3$.

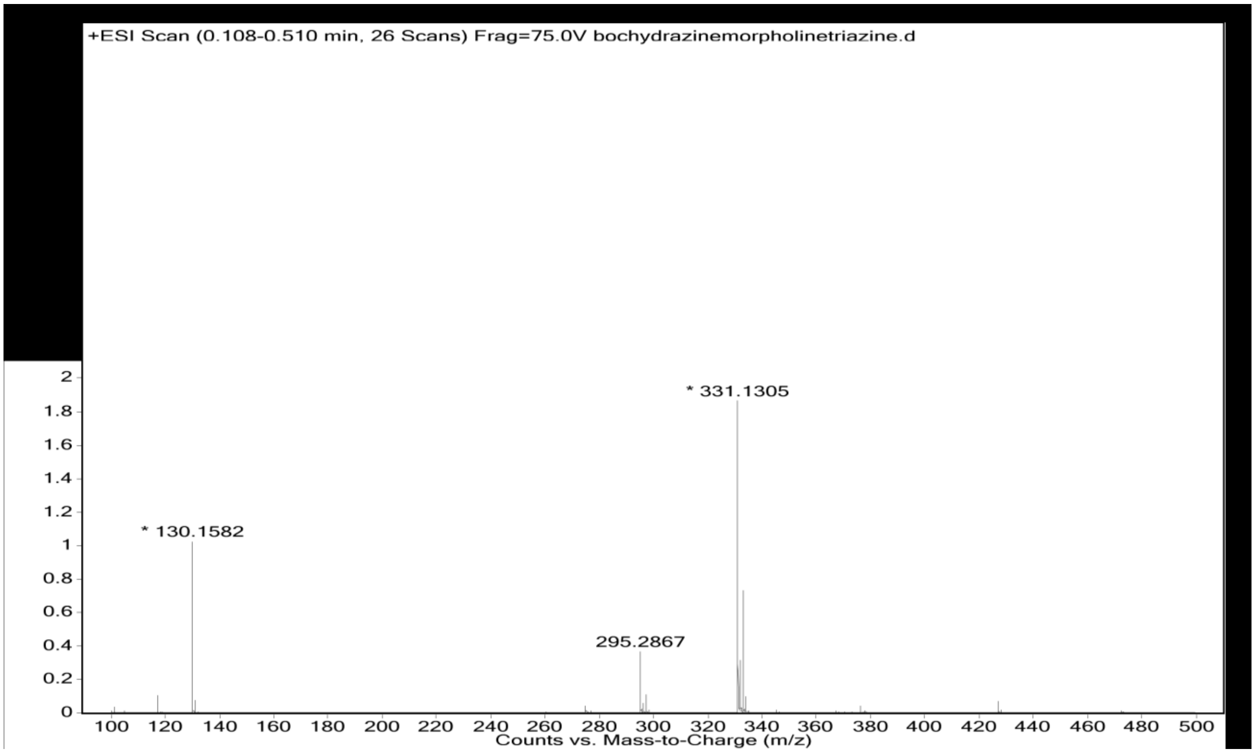

Figure S6. HR-MS spectrum of molecule **3**.

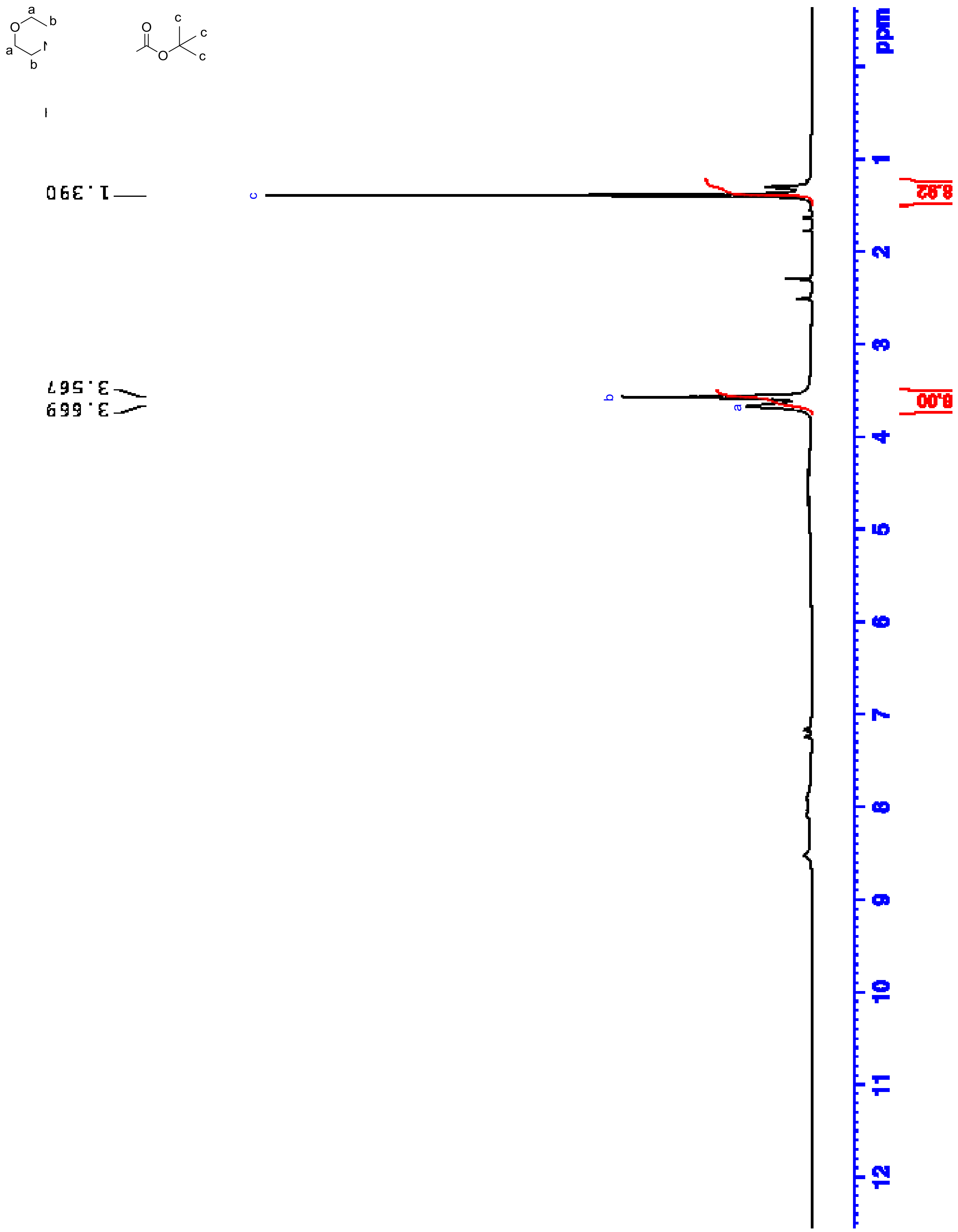

Figure S7. ^{1}H NMR spectrum of molecule **4** in $DMSO_{d-6}$.

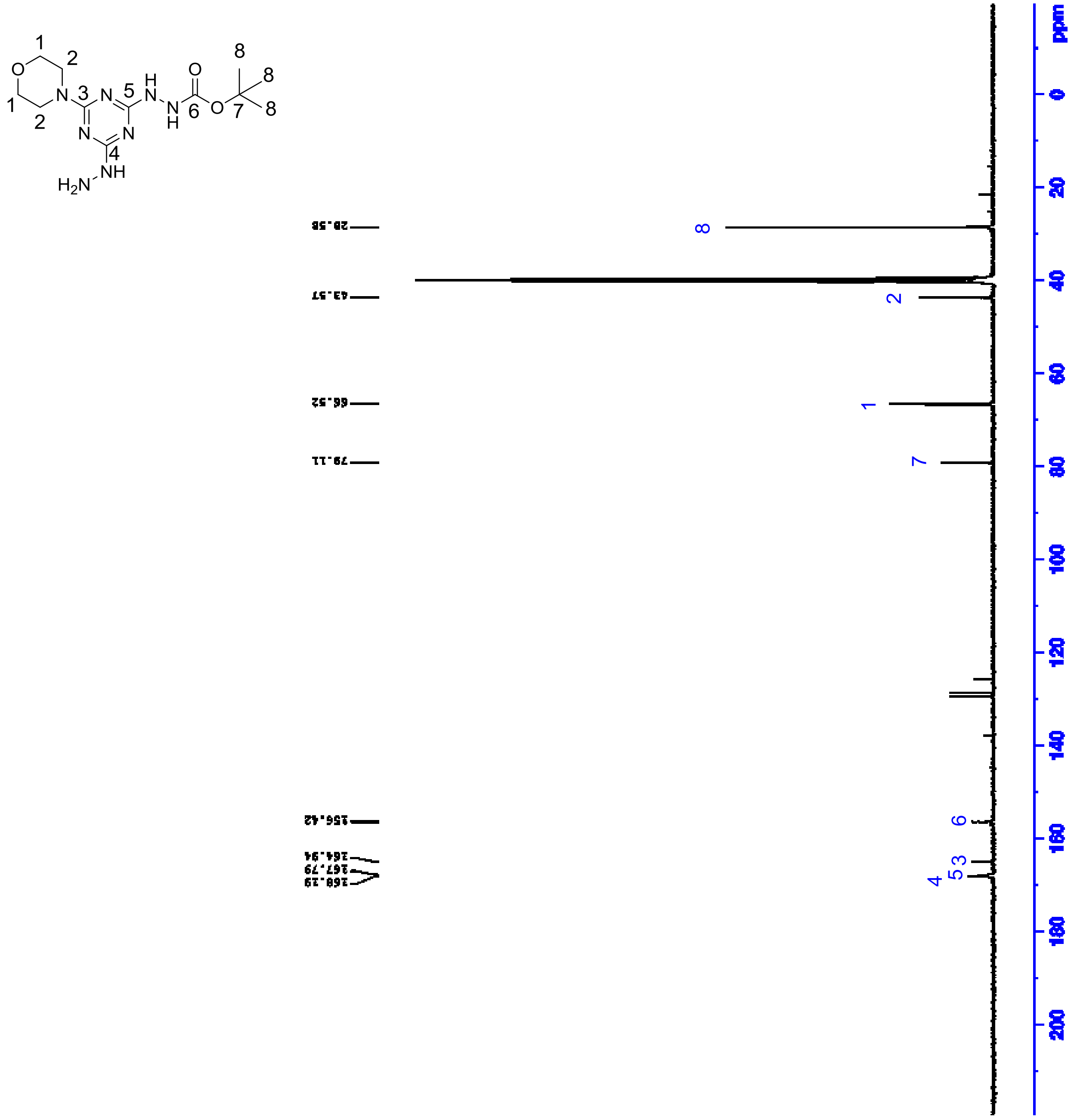

Figure S8. ^{13}C NMR spectrum of molecule **4** in $DMSO_{d-6}$.

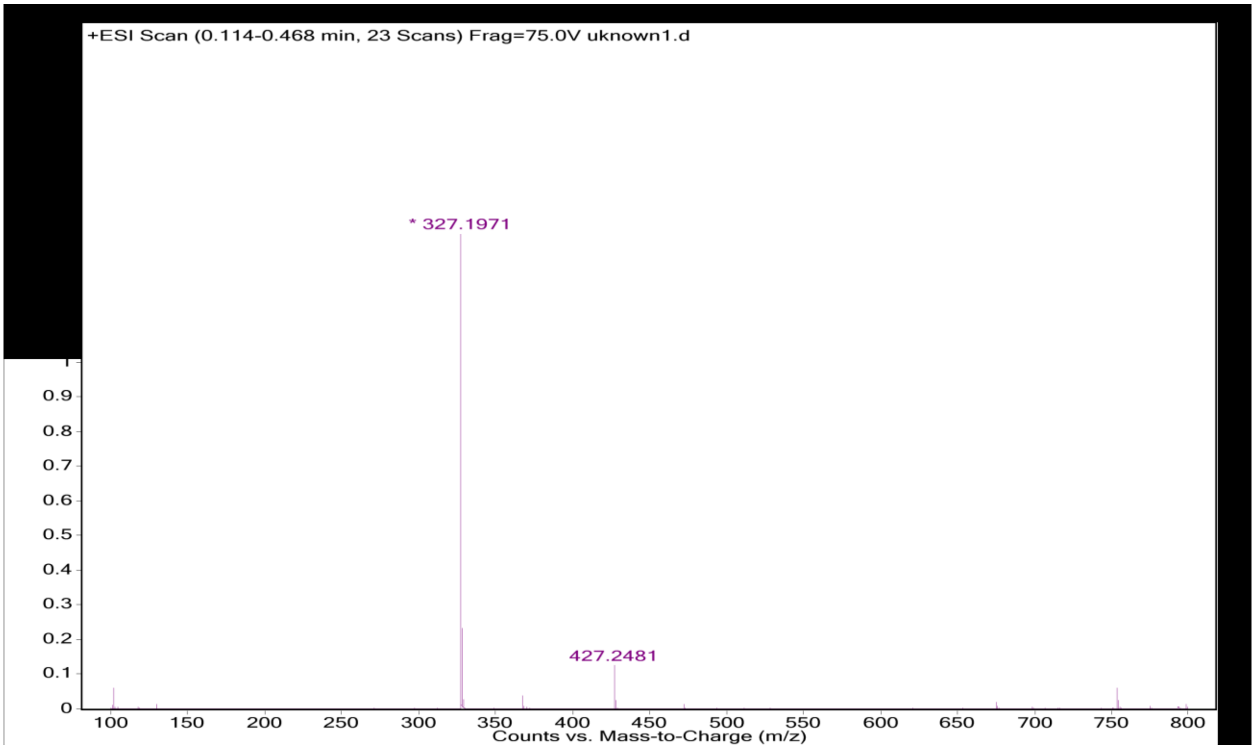

Figure S9. HR-MS spectrum of molecule **4**.

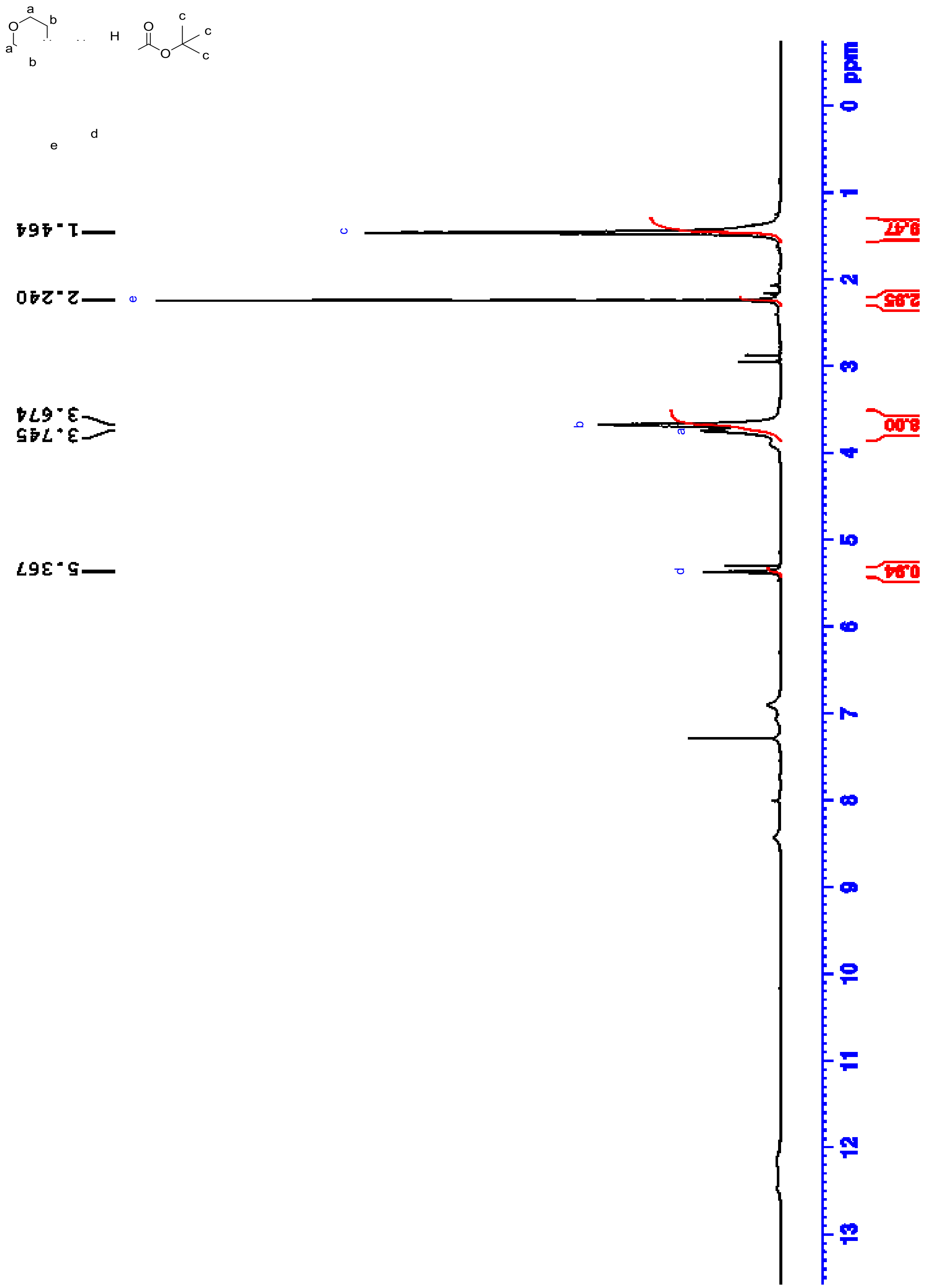

Figure S10. ^{1}H NMR spectrum of molecule **5** in $CDCl_3$.

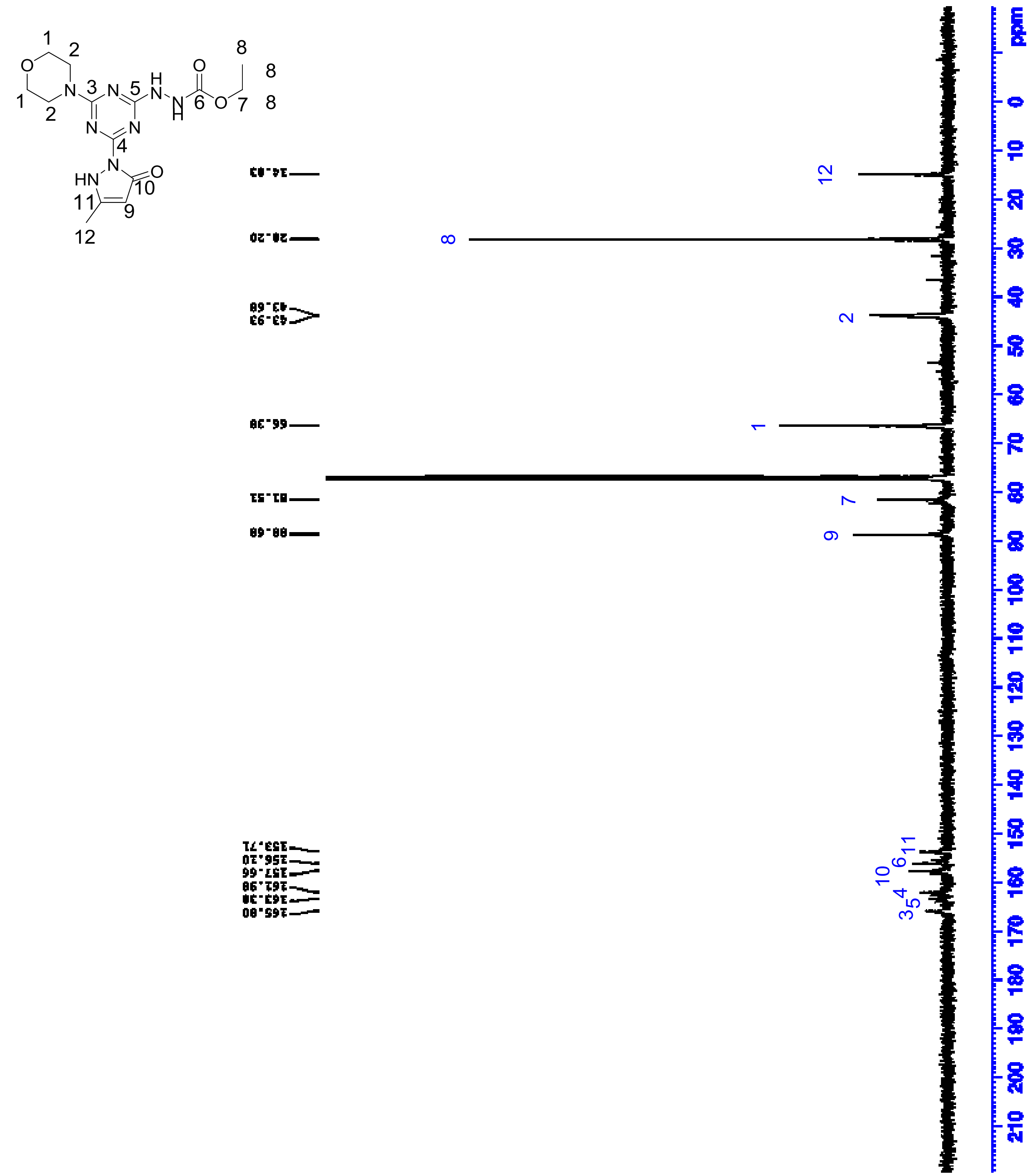

Figure S11. ^{13}C NMR spectrum of molecule **5** in $CDCl_3$.

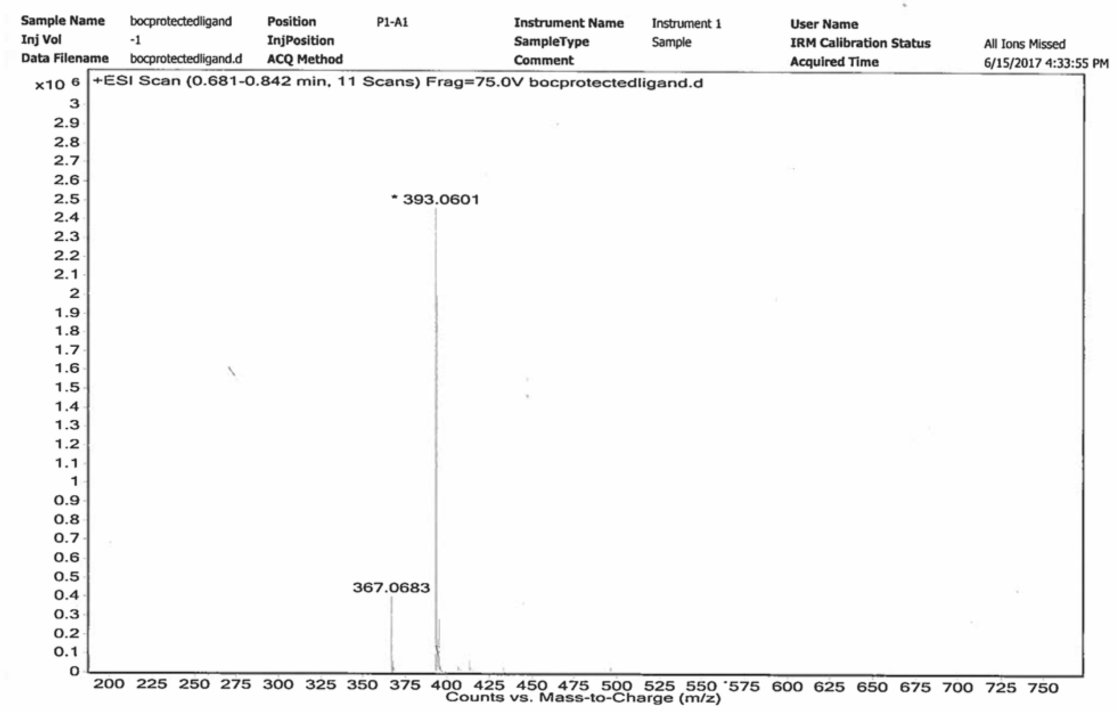

Figure S12. HR-MS spectrum of molecule **5**.

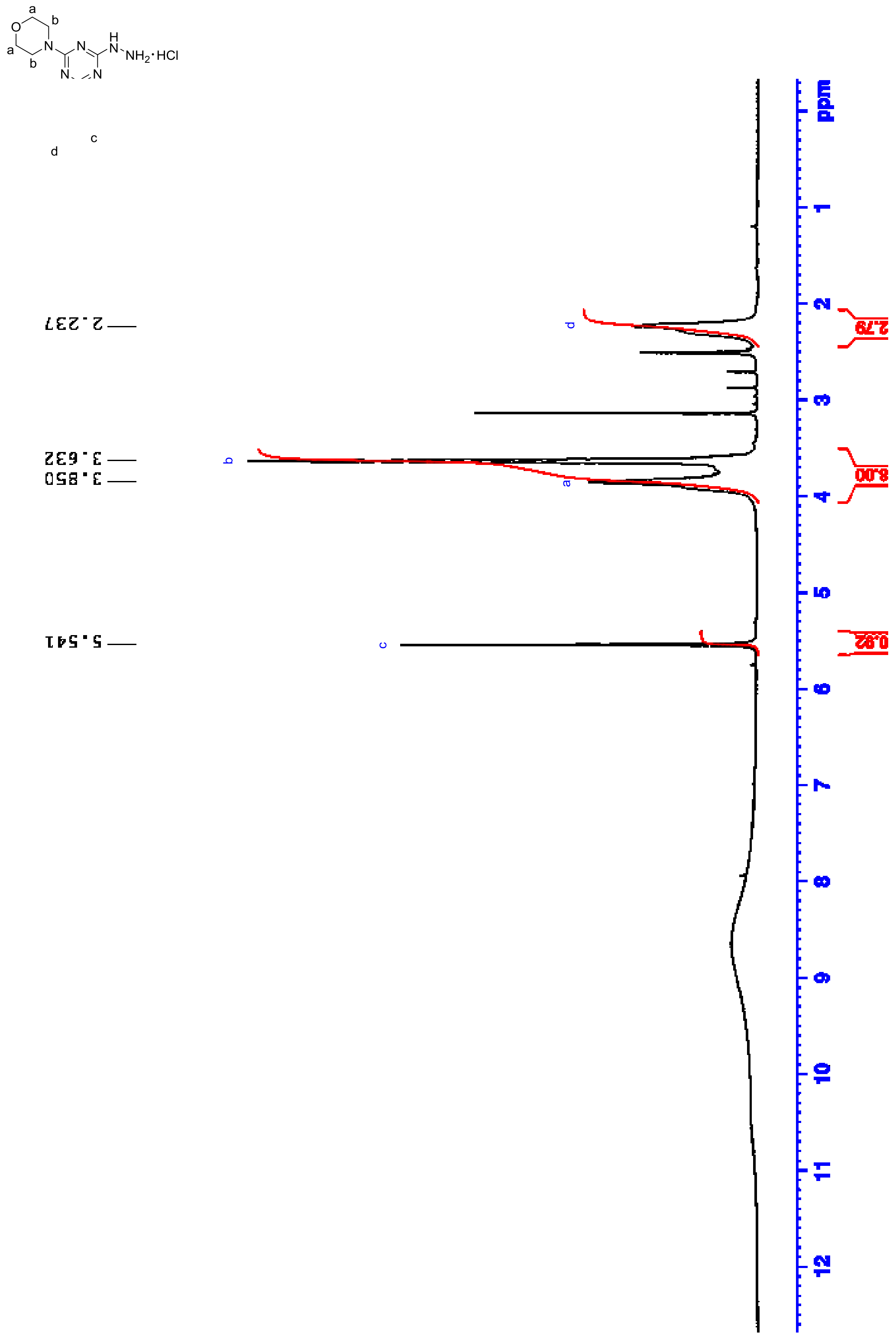

Figure S13. ^{1}H NMR spectrum of molecule **1** in $DMSO_{d\text{-}6}$.

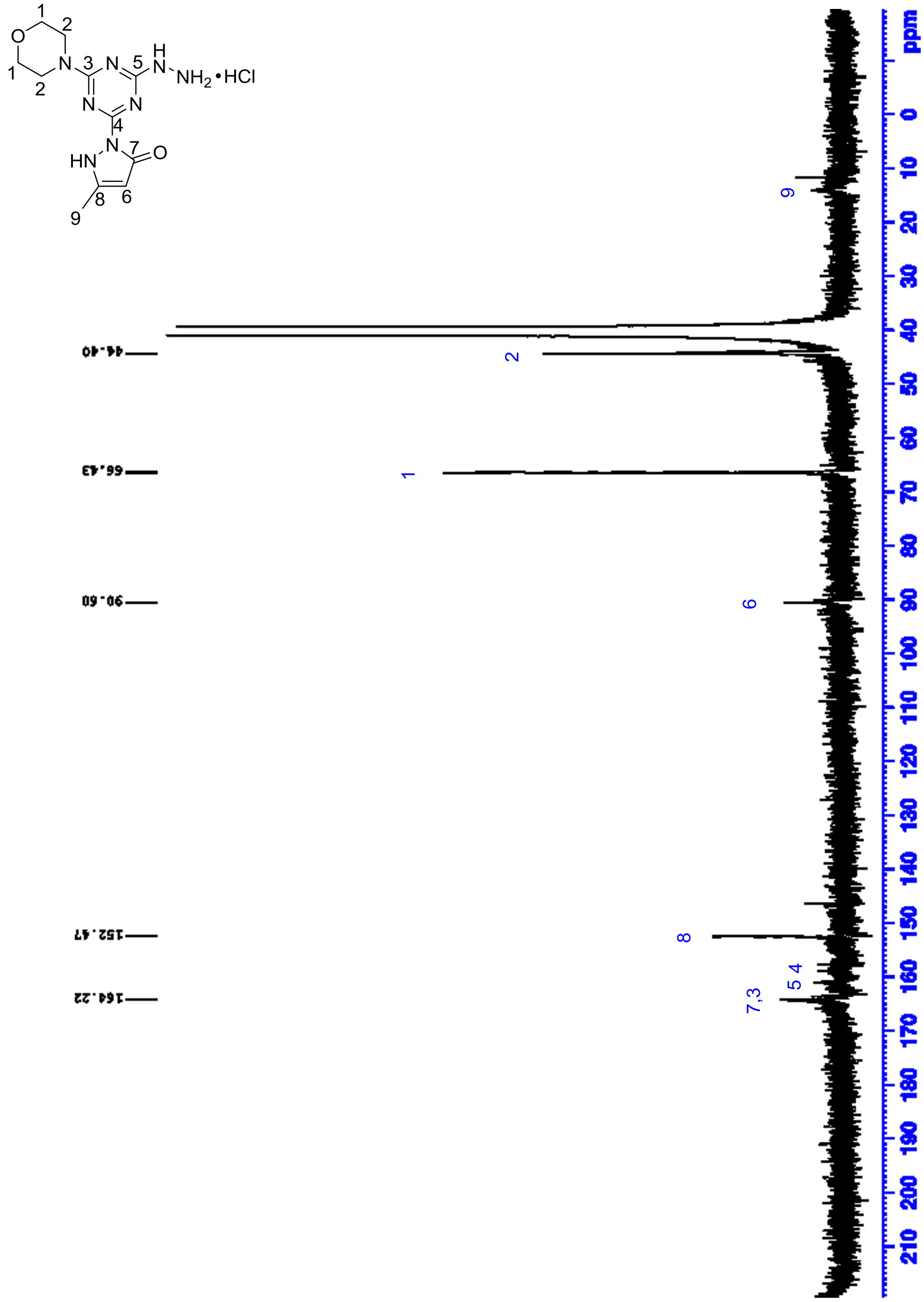

Figure S14. ^{13}C NMR spectrum of molecule **1** in $DMSO_{d-6}$.

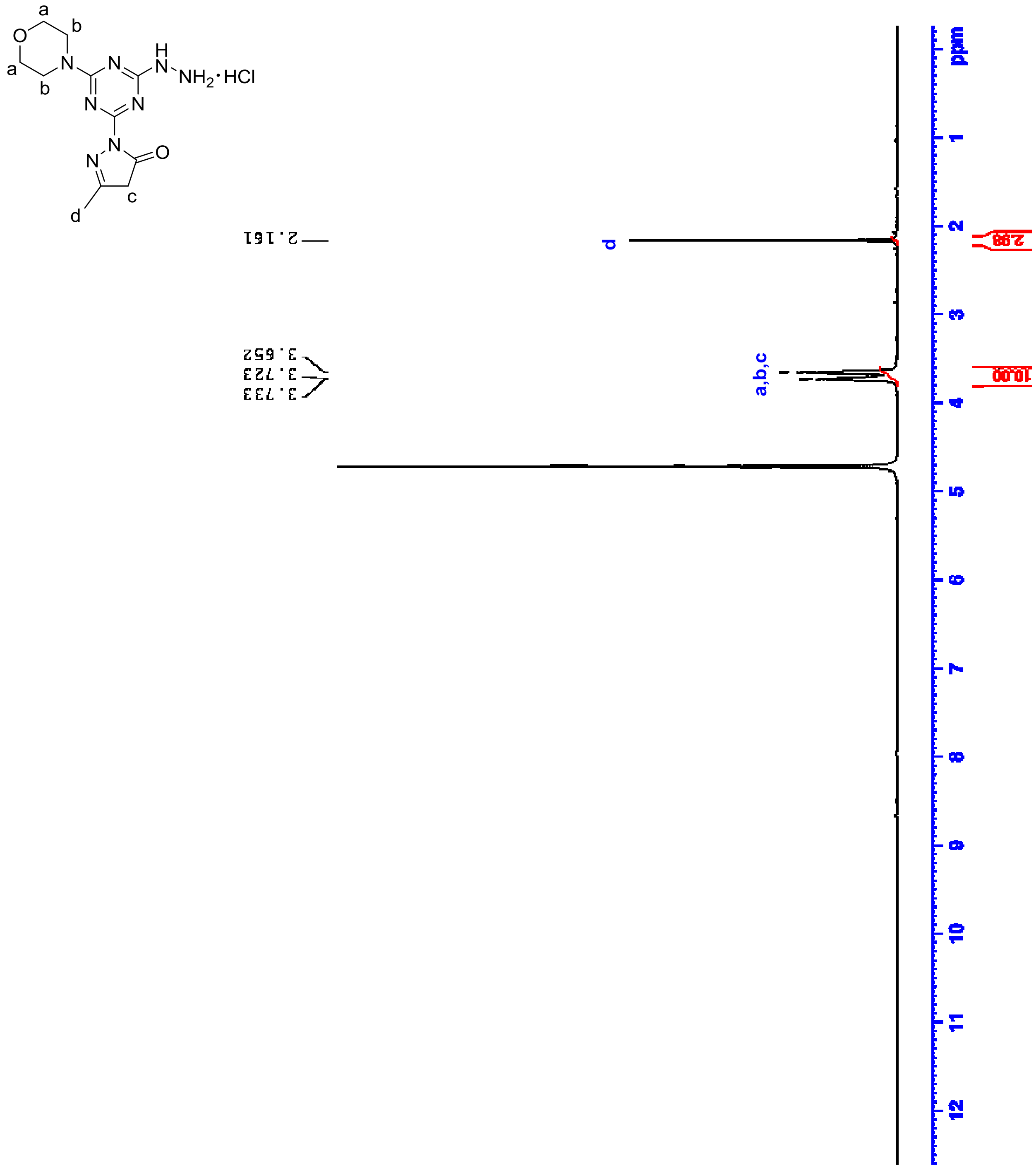

Figure S15. ^1H NMR spectrum of molecule **1** in D_2O.

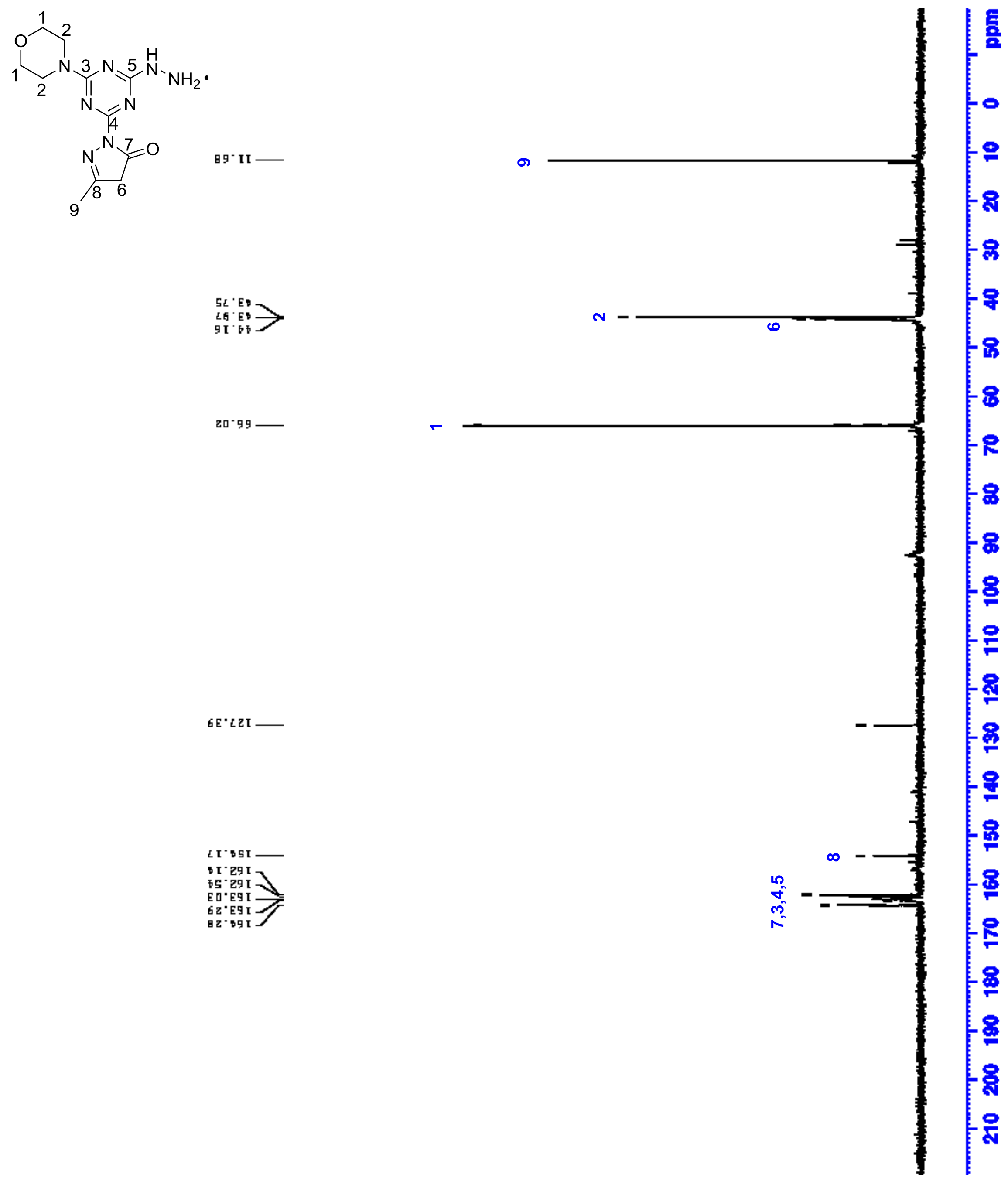

Figure S16. ^{13}C NMR spectrum of molecule **1** in D_2O.

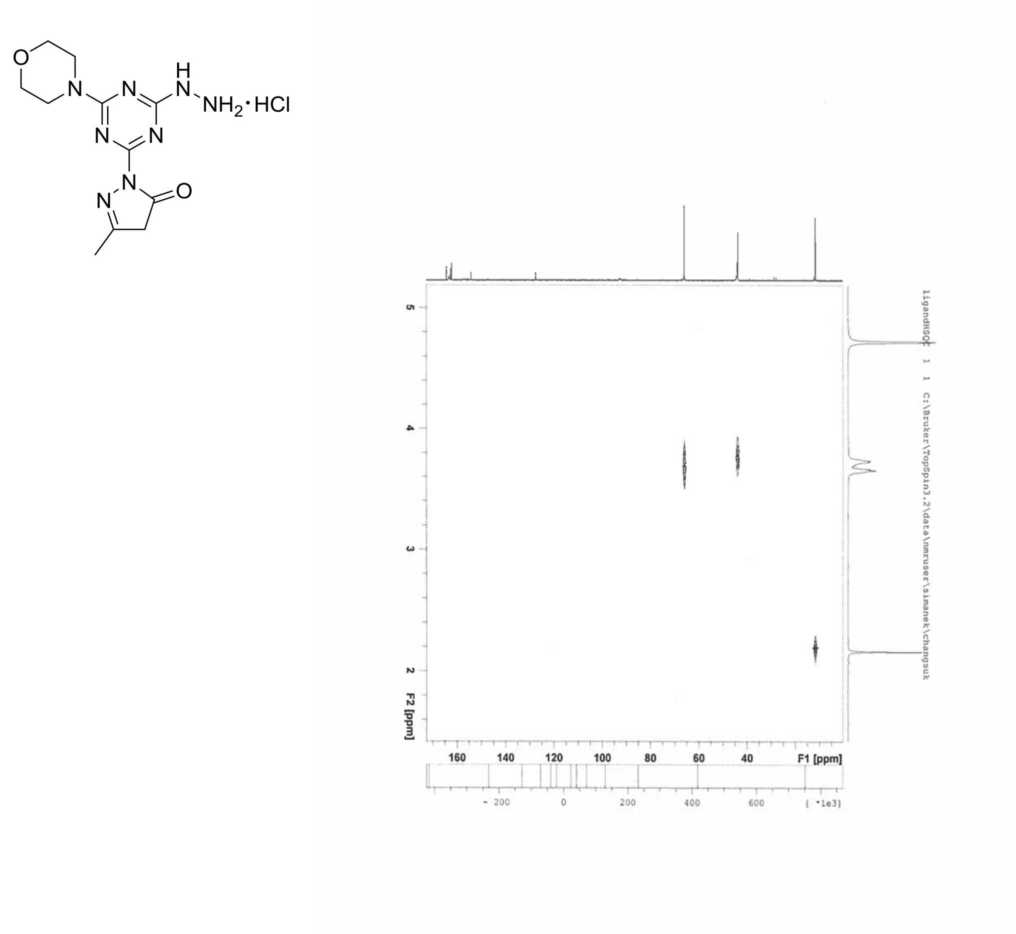

Figure S17. HSQC spectrum of molecule **1** in D_2O.

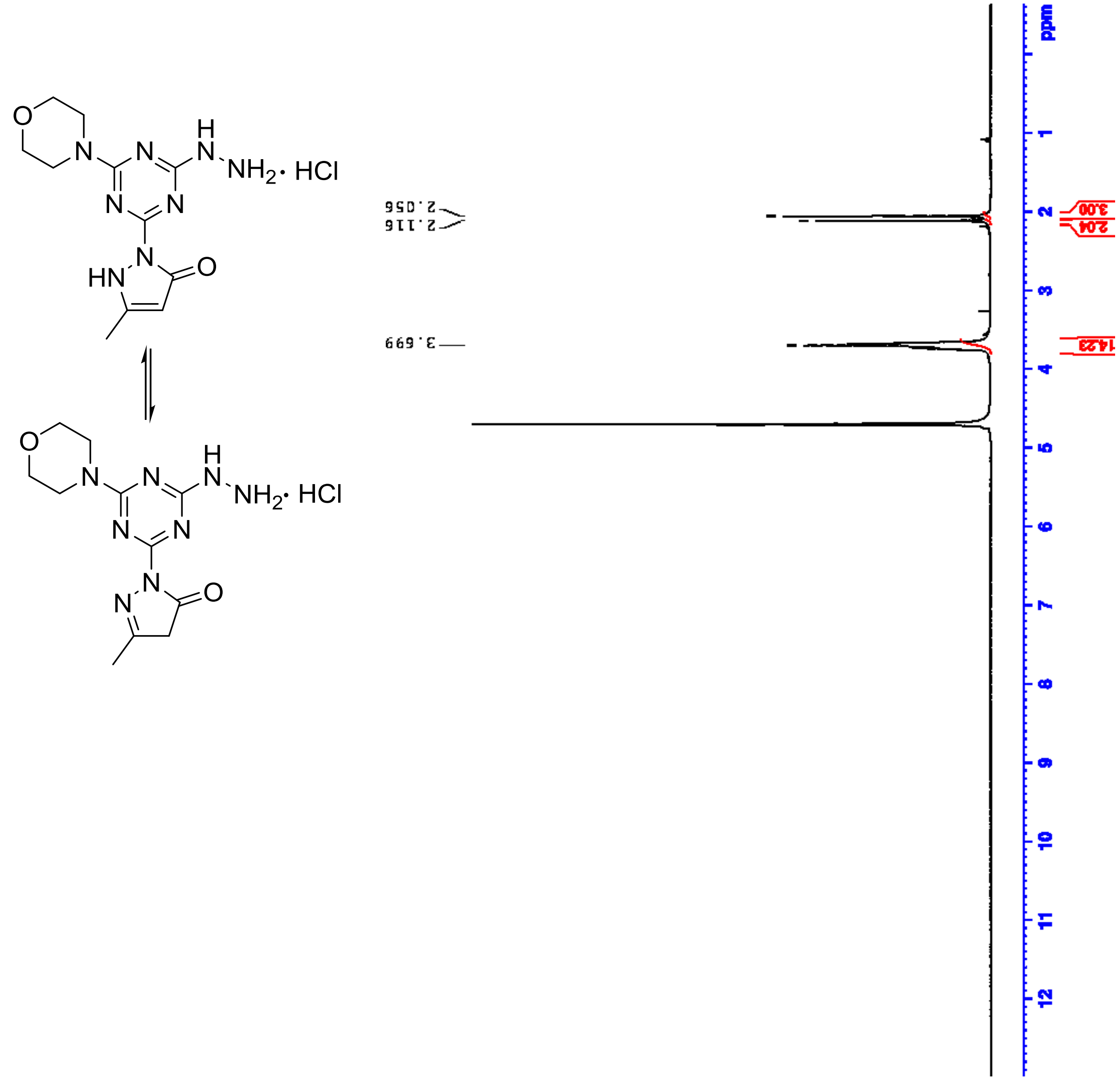

Figure S18. ^{1}H NMR spectrum of molecule **1** in D_2O after 18 h.

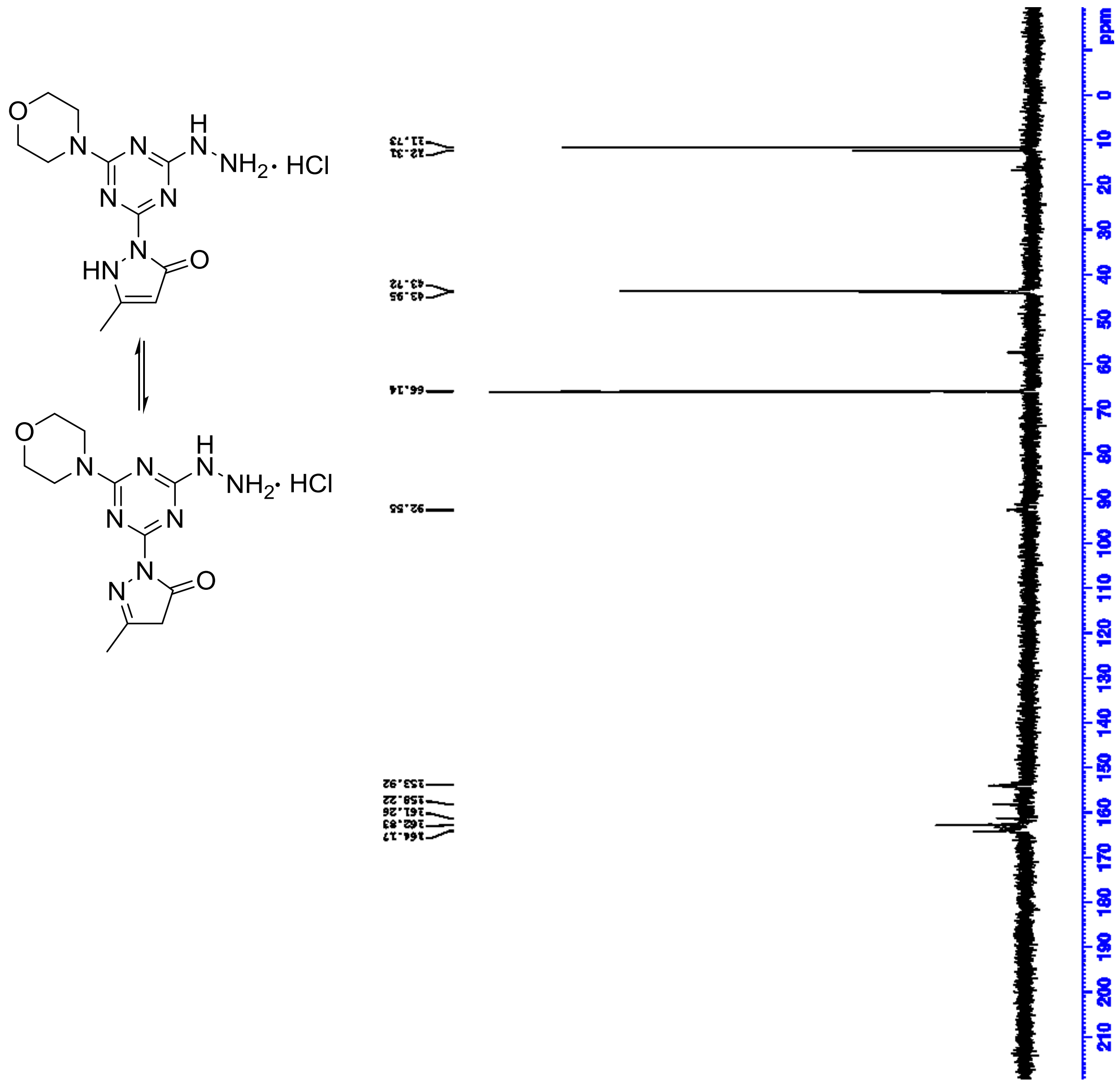

Figure S19. ^{13}C NMR spectrum of molecule **1** in D_2O after 10 h.

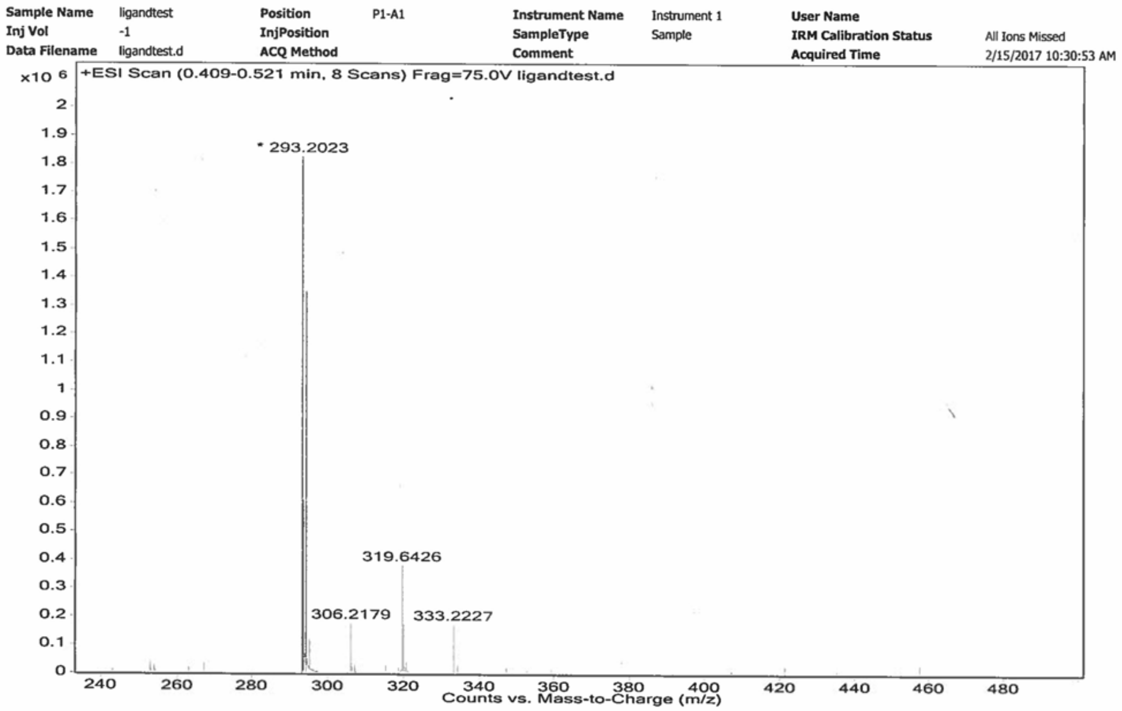

Figure S20. HR-MS spectrum of molecule **1**

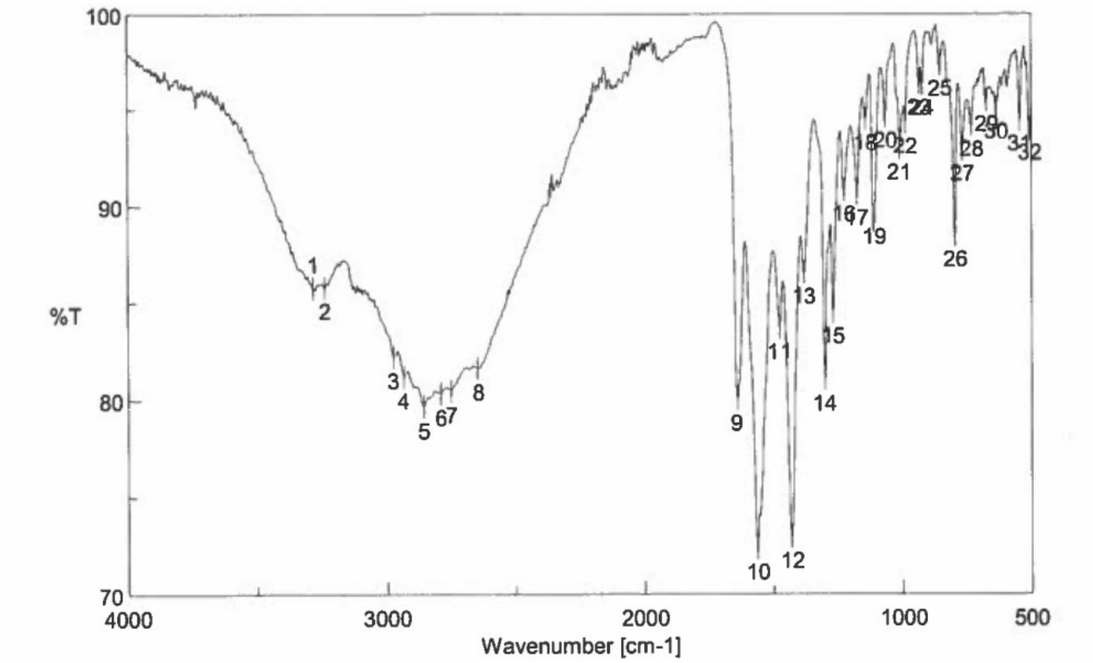

[Result of Peak Picking]

No.	Position	Intensity	No.	Position	Intensity
1	3281.29	85.7551	2	3236.93	85.7873
3	2974.66	82.2093	4	2934.16	81.1852
5	2857.99	79.6543	6	2790.49	80.3198
7	2749.99	80.4628	8	2646.82	81.6229
9	1638.23	80.0368	10	1562.06	72.3471
11	1474.31	83.7091	12	1430.92	72.9415
13	1379.82	86.5525	14	1300.75	81.0483
15	1268.93	84.5072	16	1223.61	90.8131
17	1174.44	90.6186	18	1141.65	94.4904
19	1106.94	89.648	20	1062.59	94.6217
21	1007.62	92.9836	22	985.447	94.3221
23	930.485	96.3326	24	918.914	96.2957
25	850.454	97.2907	26	793.564	88.4688
27	764.637	92.8997	28	728.961	94.1719
29	670.142	95.453	30	632.537	95.0699
31	543.828	94.4363	32	504.294	93.9651

Figure S21. IR spectrum of molecule **1**

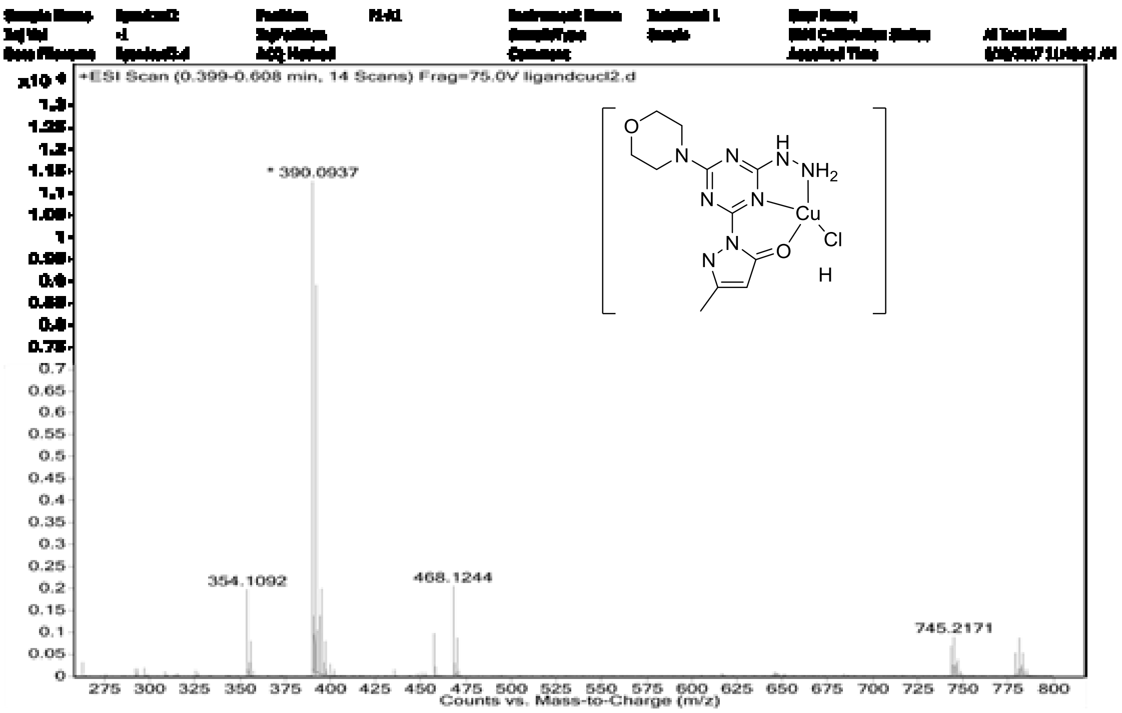

Figure S22. HR-MS spectrum of molecule **6**.

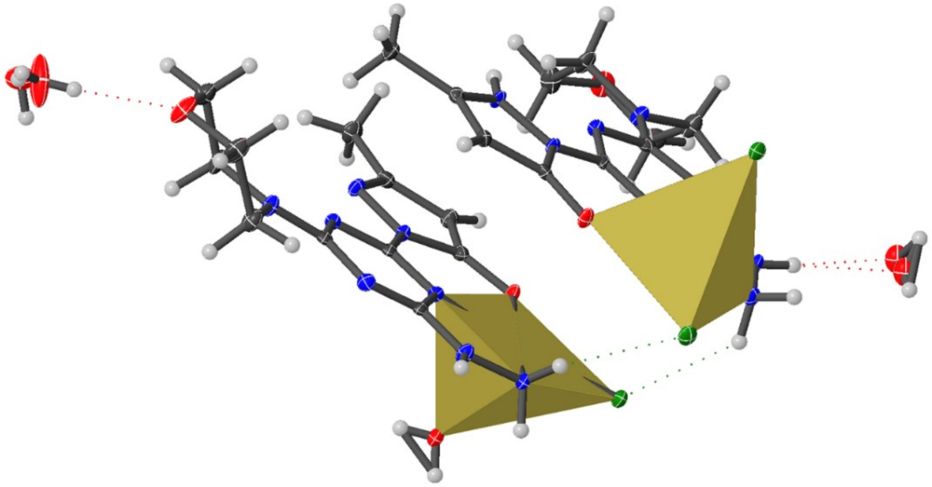

Figure S23. Molecular structure of molecule **6** in the solid state showing polyhedral at metal centers. One water molecule is not shown for clarity. Important intermolecular interactions are also highlighted.

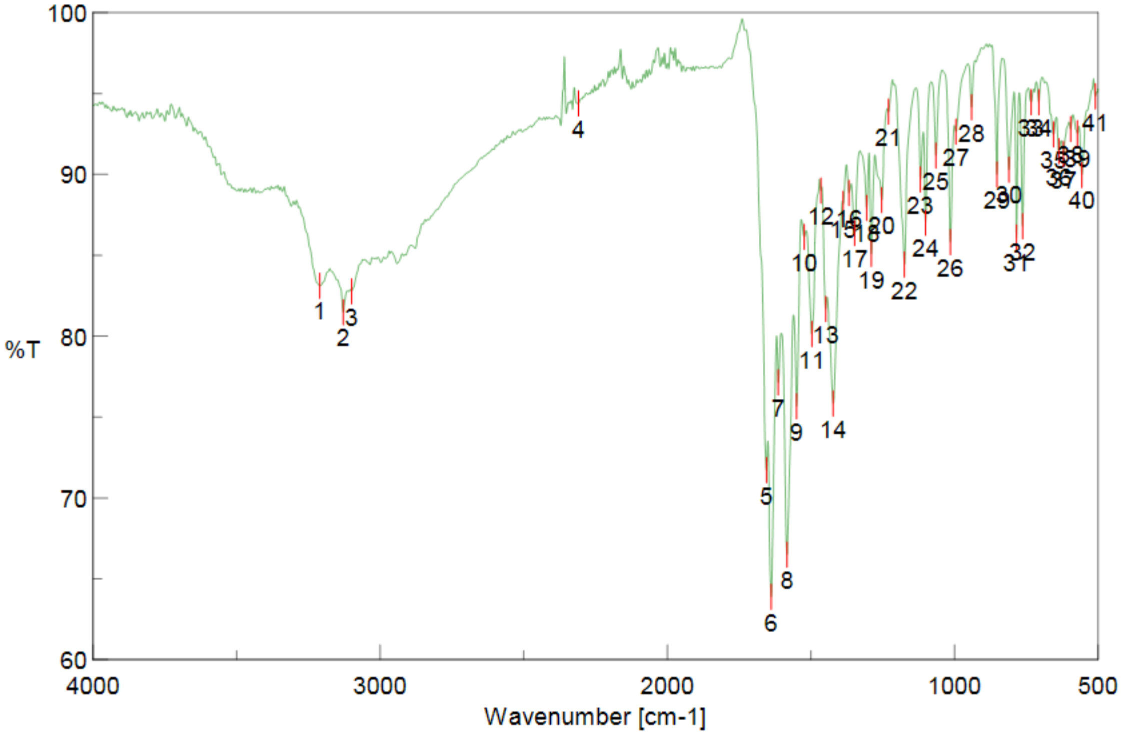

[Result of Peak Picking]

No.	Position	Intensity	No.	Position	Intensity
1	3210.9	83.1011	2	3128.94	81.4593
3	3100.01	82.7528	4	2309.34	94.3672
5	1654.62	71.7014	6	1638.23	63.8692
7	1613.16	77.137	8	1583.27	66.4837
9	1549.52	75.6659	10	1523.49	86.1289
11	1496.49	80.1218	12	1464.67	88.9813
13	1448.28	81.6685	14	1422.24	75.8206
15	1387.53	88.1591	16	1367.28	88.8438
17	1348	86.3666	18	1305.57	87.9027
19	1289.18	85.0627	20	1253.5	88.4072
21	1229.4	93.8612	22	1174.44	84.4109
23	1118.51	89.6799	24	1100.19	86.9966
25	1064.51	91.1438	26	1014.37	85.7977
27	995.089	92.6249	28	940.128	94.1459
29	852.382	89.9838	30	809.956	90.2858
31	783.922	86.0952	32	762.709	86.7845
33	732.817	94.4544	34	706.783	94.4431
35	654.715	92.454	36	636.394	91.427
37	620.002	91.2421	38	595.896	92.7882
39	571.79	92.5301	40	556.363	89.9547
41	509.115	94.816			

Figure S24. IR spectrum of molecule **6**.

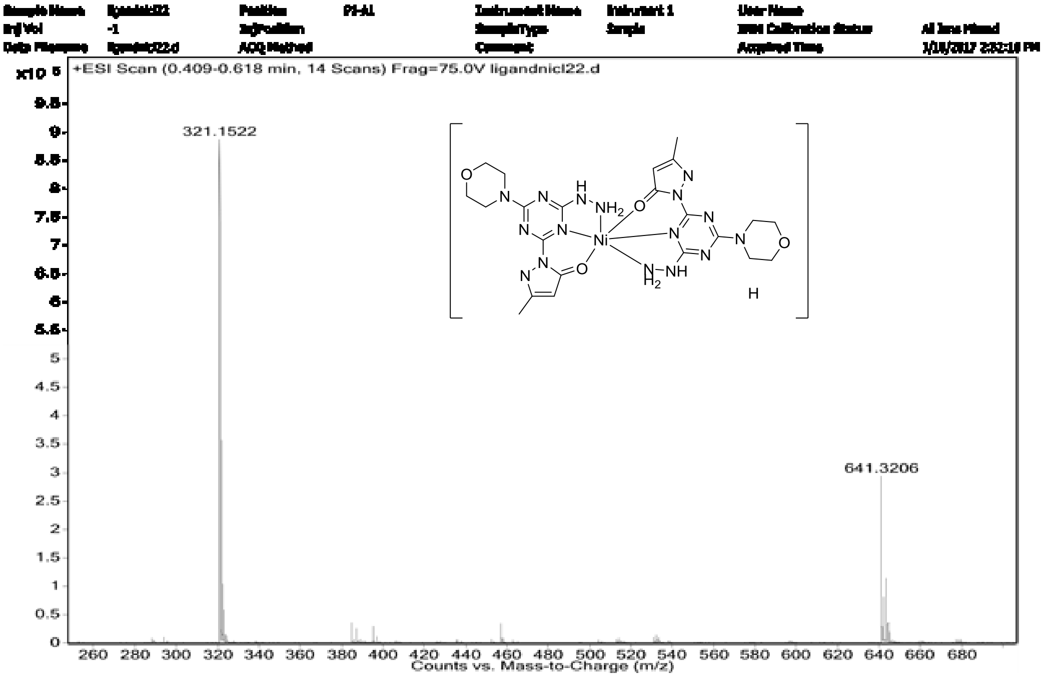

Figure S25. HR-MS spectrum of molecule **7**.

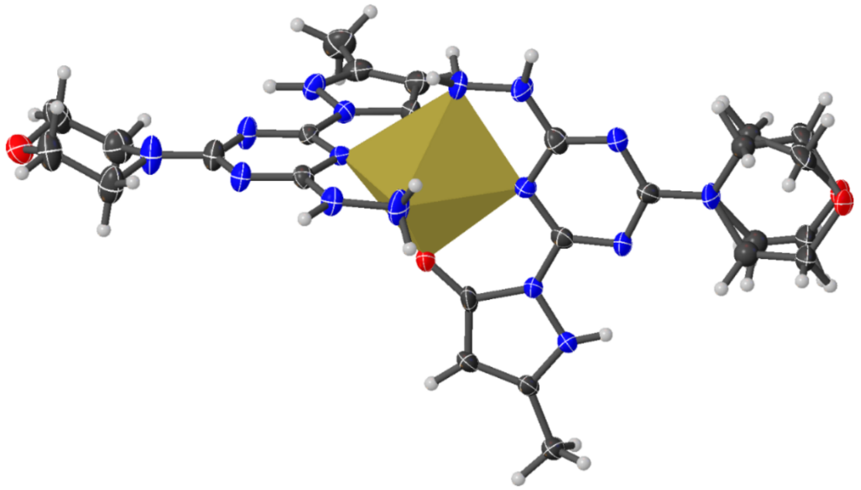

Figure S26. Molecular structure of **7** in the solid state showing distorted polyhedral at metal center. Solvent molecules and Cl^- counter ions are not shown for clarity.

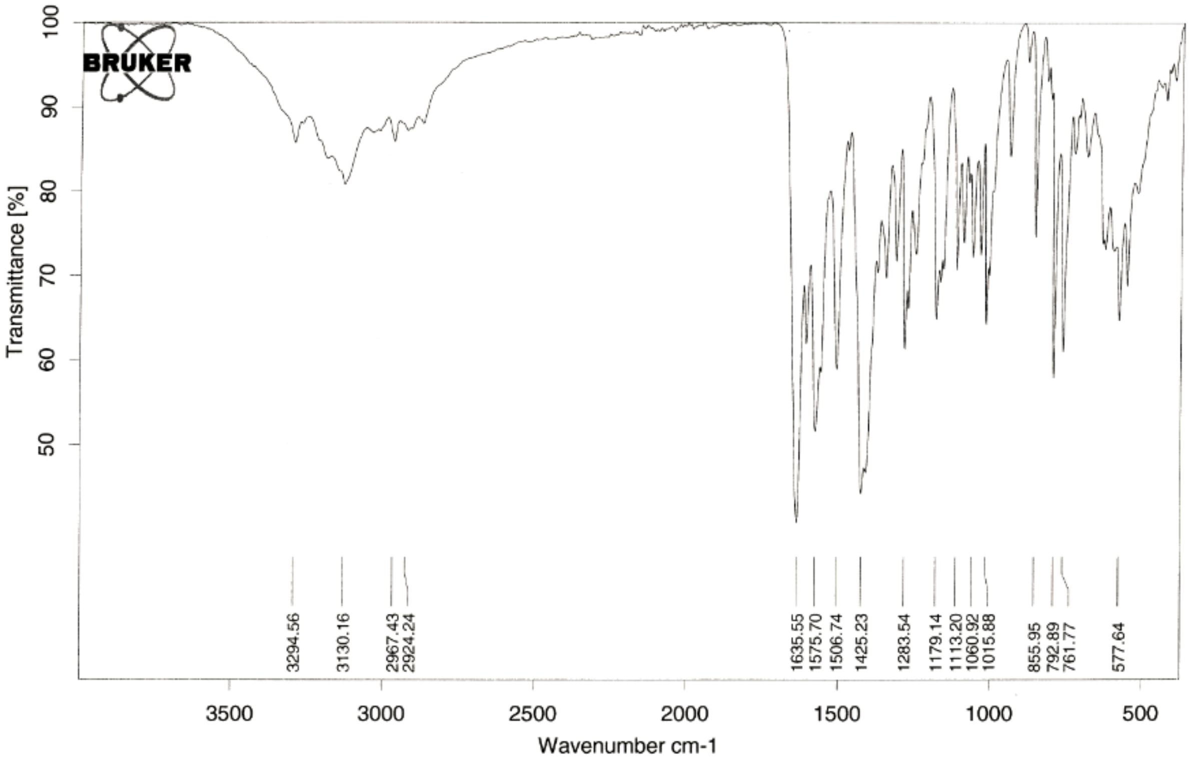

Figure S27. IR spectrum of molecule **7**.

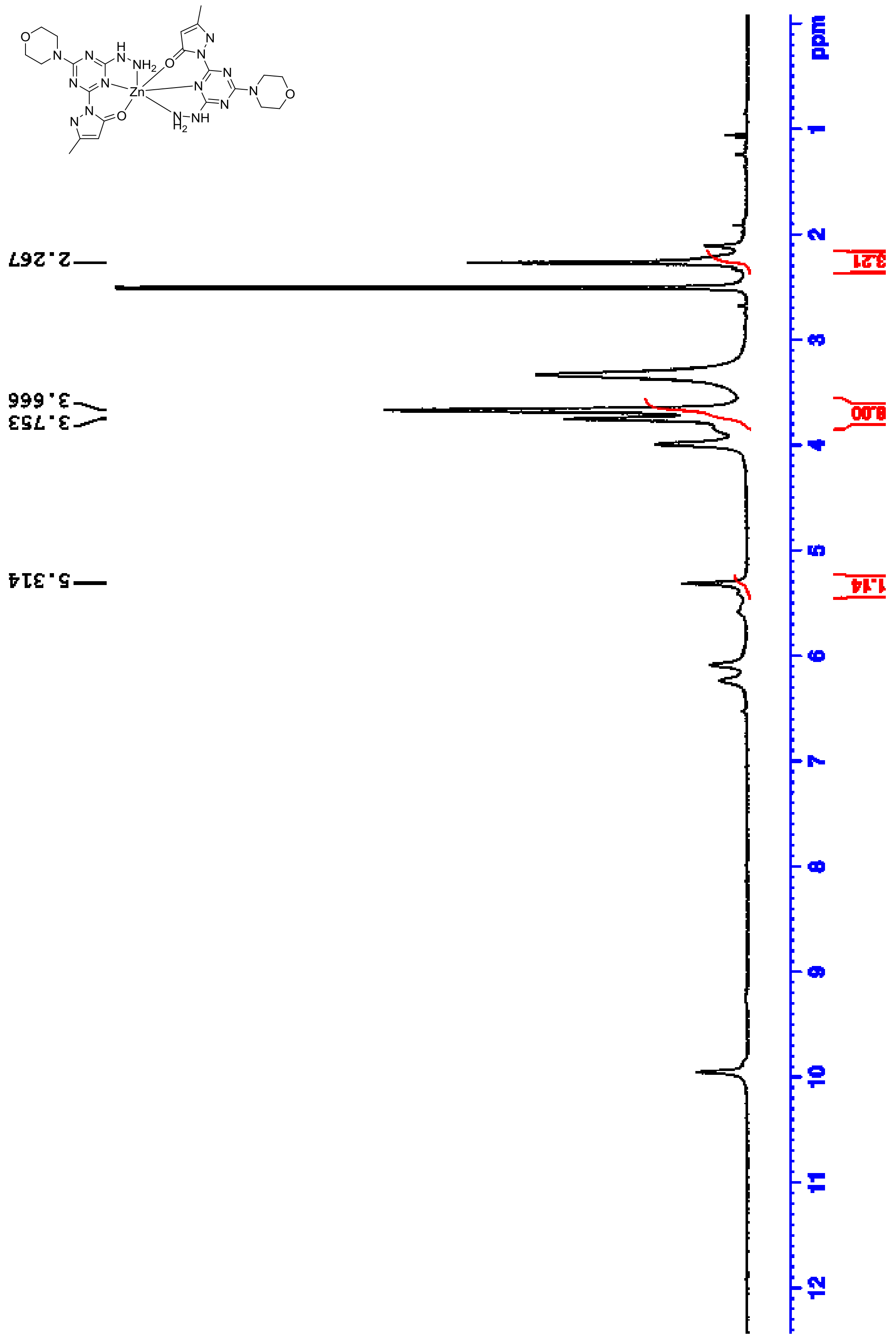

Figure S28. ^{1}H NMR spectrum of molecule **8** in $DMSO_{d-6}$.

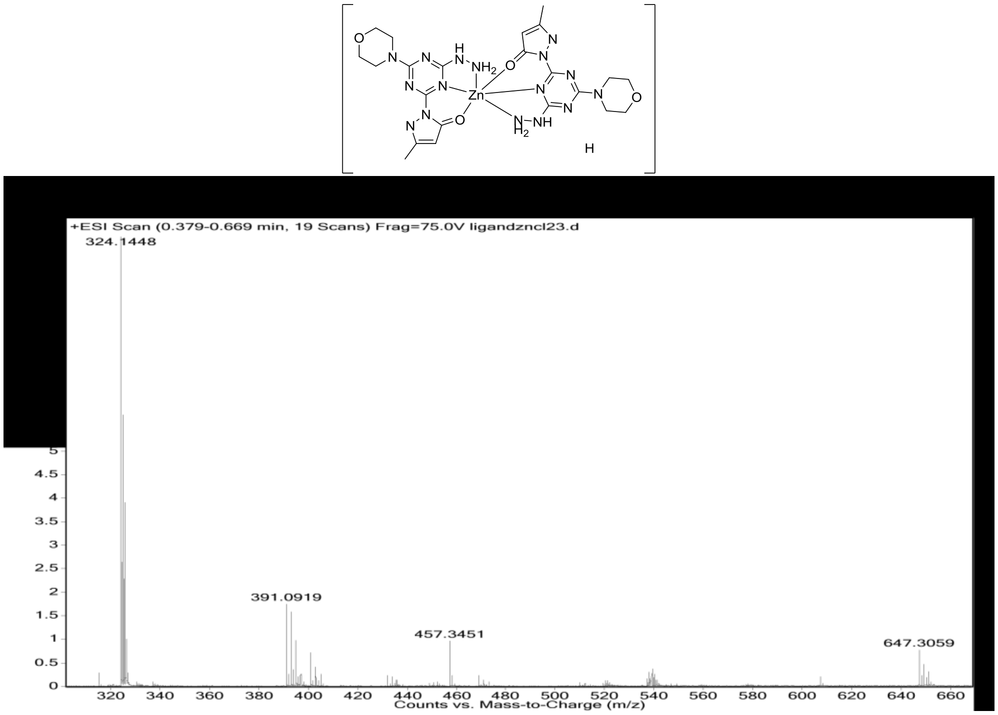

Figure S29. HR-MS spectrum of molecule **8**.

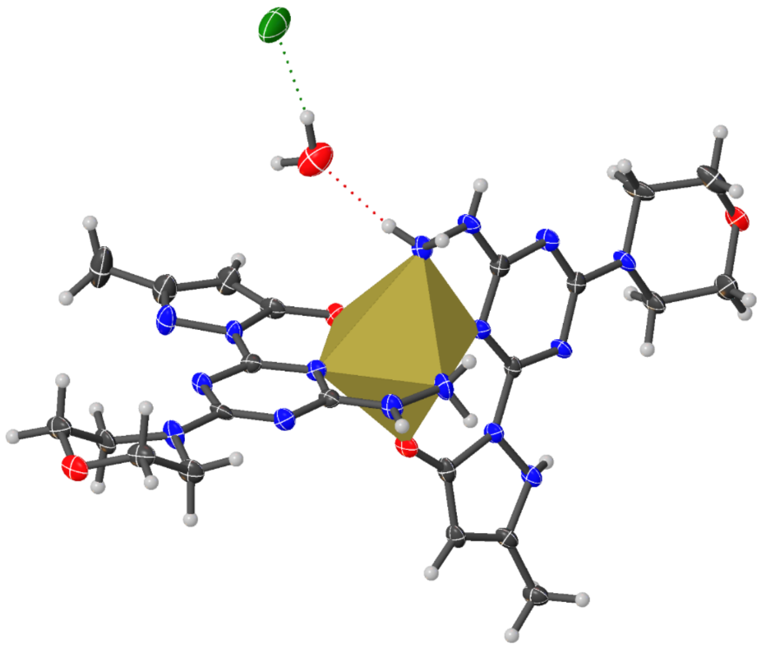

Figure S30. Molecular structure of **8** in the solid state showing distorted polyhedral at metal center. Eight solvent molecules are not shown for clarity. Bridged H-bonding interaction due to water molecule between main structural motif and counter Cl- ion is also highlighted.

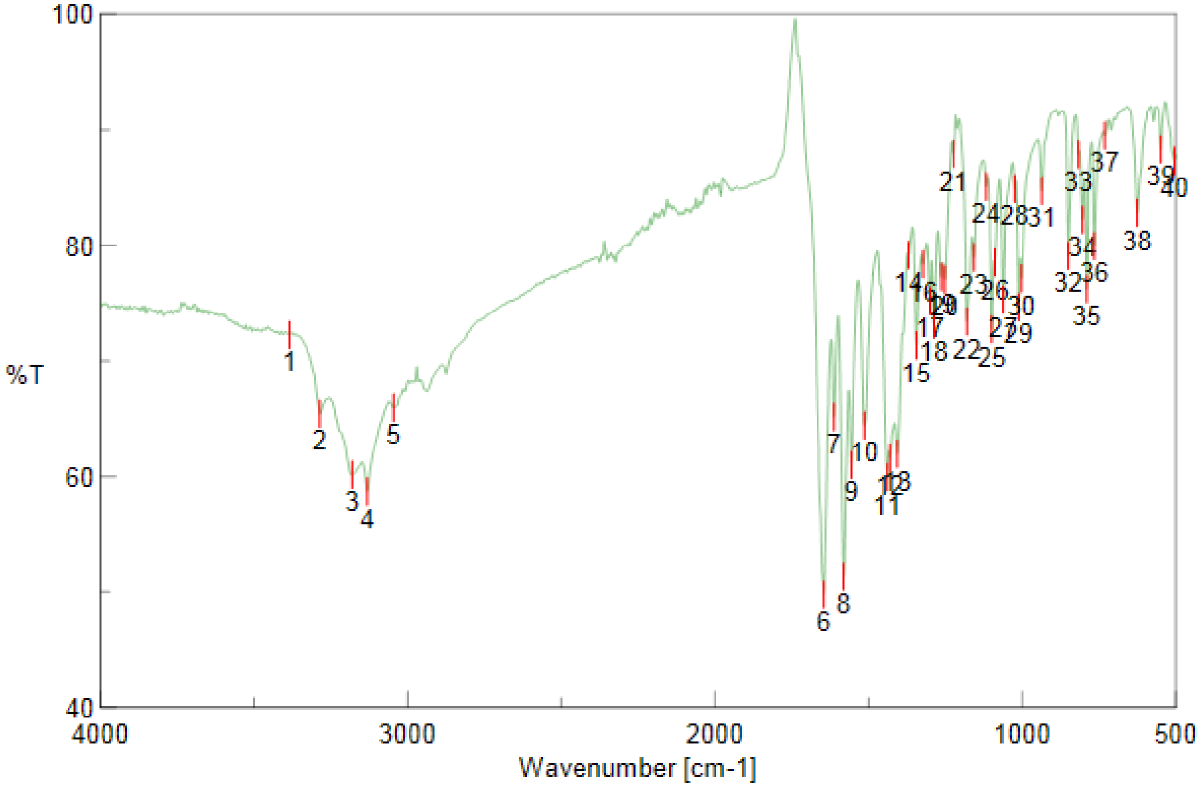

[Result of Peak Picking]

No.	Position	Intensity	No.	Position	Intensity
1	3384.46	72.2285	2	3285.14	65.4052
3	3180.04	60.1189	4	3131.83	58.6738
5	3046.01	65.9216	6	1644.98	49.785
7	1612.2	65.1177	8	1581.34	51.2941
9	1556.27	60.977	10	1512.88	64.3788
11	1439.6	59.8973	12	1429.96	61.6066
13	1405.85	61.9315	14	1370.18	79.1254
15	1345.11	71.3164	16	1321.96	78.3454
17	1299.79	75.183	18	1287.25	73.1171
19	1264.11	77.2374	20	1252.54	77.0248
21	1223.61	87.8935	22	1179.26	73.3932
23	1157.08	78.947	24	1117.55	85.049
25	1098.26	72.7288	26	1088.62	78.5126
27	1061.62	75.2963	28	1024.02	84.8797
29	1012.45	74.6221	30	1001.84	77.1253
31	936.271	84.6932	32	849.49	79.0745
33	816.706	87.837	34	804.171	82.1936
35	790.671	76.2043	36	764.637	79.9191
37	730.889	89.476	38	624.823	82.7911
39	548.649	88.296	40	504.294	87.3111

Figure S31. IR spectrum of molecule **8**.

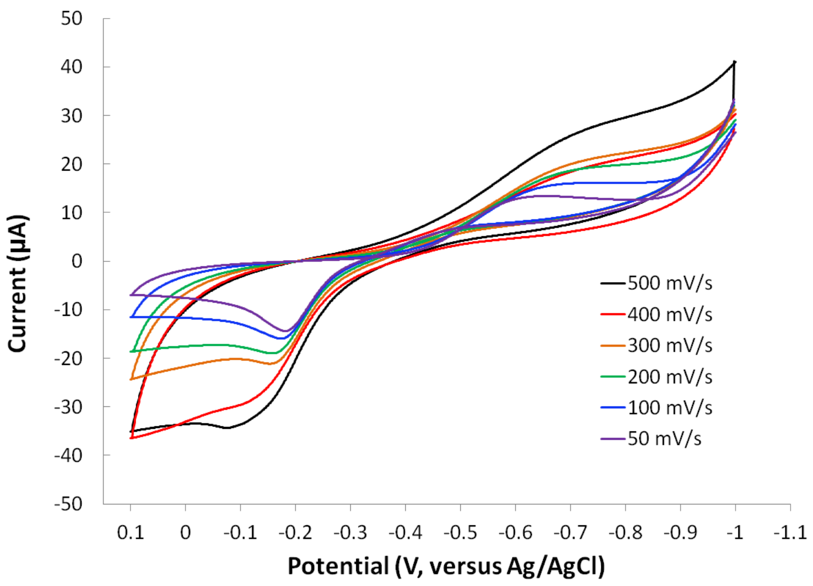

Figure S32. Cyclic voltammogram of complex **6** (3.3 mM) obtained in a 0.1 M KCl aqueous solution using a glassy carbon working electrode, platinum auxiliary electrode, and Ag/AgCl reference electrode at multiple scan rates.

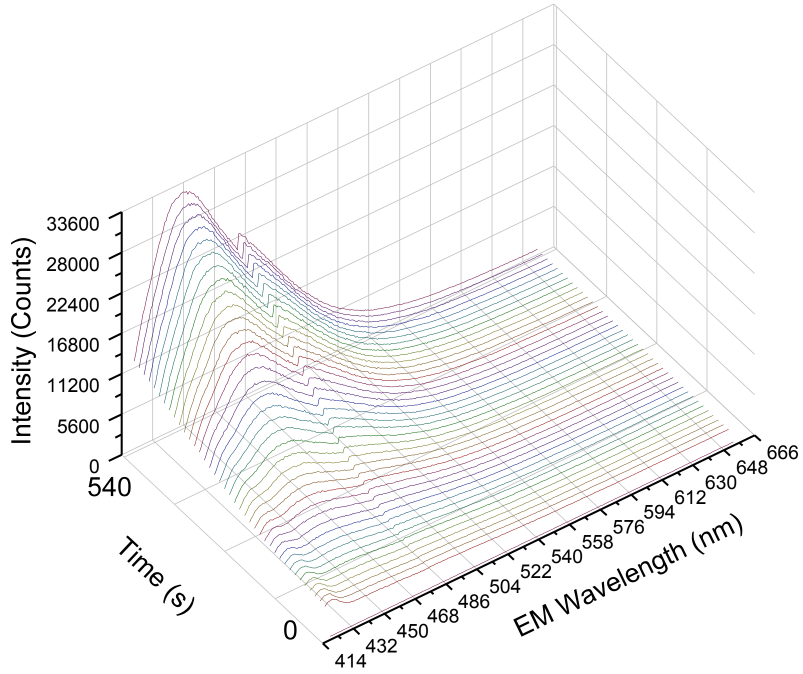

Figure S33. Fluorescence measured for CCA [500 μM] and ascorbate [300 μM] with Cu(II) [10 μM].

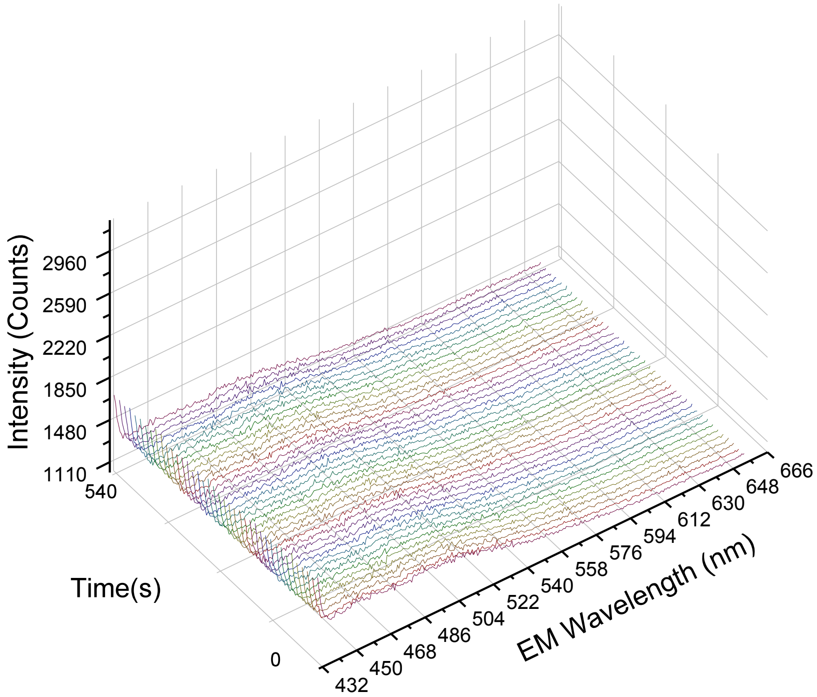

Figure S34. Fluorescence measured for CCA [500 µM] and ascorbate [300 µM] with Cu(II) [10 µM] and molecule **1** [10 µM] .

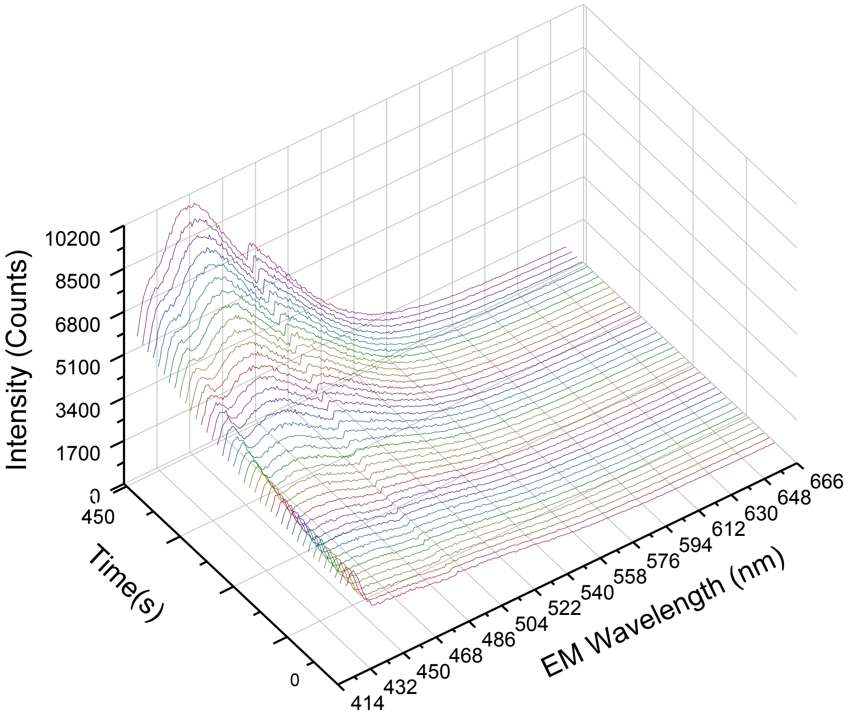

Figure S35. Fluorescence measured for CCA [500 µM] and ascorbate [300 µM] with Cu(II) [10 µM] and molecule **1** [5 µM].

Table S2. Geometric parameters *i.e.* bond lengths and angles (Å, º) for **6**.

Bond lengths			
O8A—H8AA	0.87 (5)	Cu2—Cl2	2.2778 (7)
O8A—H8AB	1.12 (5)	Cu2—Cl3	2.5706 (8)
O8B—H8AA	0.70 (5)	Cu2—O5	1.9418 (19)
O8B—H8AB	0.57 (5)	Cu2—N11	1.962 (2)
Cu1—Cl1	2.2767 (7)	Cu2—N14	2.013 (2)
Cu1—O2	1.910 (2)	O4—C12	1.424 (4)
Cu1—O3	2.306 (2)	O4—C13	1.429 (4)
Cu1—N4	1.953 (2)	O5—C19	1.272 (3)
Cu1—N6	2.018 (2)	N9—C14	1.468 (3)
O1—C1	1.430 (4)	N9—C15	1.466 (4)
O1—C3	1.429 (4)	N9—C16	1.328 (4)
O2—C8	1.294 (3)	N10—C16	1.355 (3)
O3—H3A	0.69 (4)	N10—C17	1.327 (4)
O3—H3B	0.71 (5)	N11—C17	1.359 (4)
N1—C2	1.459 (4)	N11—C18	1.337 (3)
N1—C4	1.465 (4)	N12—C16	1.369 (4)
N1—C5	1.341 (4)	N12—C18	1.301 (4)
N2—C5	1.348 (4)	N13—H13	0.83 (3)
N2—C7	1.324 (4)	N13—N14	1.418 (3)
N3—C5	1.361 (4)	N13—C17	1.341 (3)
N3—C6	1.315 (4)	N14—H14A	0.86 (4)
N4—C6	1.345 (3)	N14—H14B	0.88 (4)
N4—C7	1.354 (4)	N15—N16	1.380 (3)
N5—H5	0.79 (3)	N15—C18	1.393 (4)
N5—N6	1.421 (3)	N15—C19	1.399 (3)
N5—C7	1.341 (4)	N16—C21	1.338 (4)
N6—H6A	0.85 (4)	N16—H16	0.82 (4)
N6—H6B	0.92 (4)	C12—H12A	0.9900
N7—N8	1.398 (3)	C12—H12B	0.9900
N7—C6	1.376 (4)	C12—C14	1.511 (4)
N7—C8	1.408 (4)	C13—H13A	0.9900
N8—C10	1.319 (4)	C13—H13B	0.9900
C1—H1A	0.9900	C13—C15	1.518 (4)

C1—H1B	0.9900	C14—H14C	0.9900
C1—C2	1.521 (4)	C14—H14D	0.9900
C2—H2A	0.9900	C15—H15A	0.9900
C2—H2B	0.9900	C15—H15B	0.9900
C3—H3C	0.9900	C19—C20	1.404 (4)
C3—H3D	0.9900	C20—H20	0.9500
C3—C4	1.512 (4)	C20—C21	1.378 (4)
C4—H4A	0.9900	C21—C22	1.488 (4)
C4—H4B	0.9900	C22—H22A	0.9800
C8—C9	1.374 (4)	C22—H22B	0.9800
C9—H9	0.9500	C22—H22C	0.9800
C9—C10	1.407 (4)	O6—H6C	0.78 (4)
C10—C11	1.489 (4)	O6—H6D	0.68 (5)
C11—H11A	0.9800	O7—H7A	0.76 (5)
C11—H11B	0.9800	O7—H7B	0.72 (5)
C11—H11C	0.9800	O10—O10^{i}	1.646 (16)
Bond angles			
H8AA—O8A—H8AB	60 (4)	Cl2—Cu2—Cl3	103.37 (3)
H8AA—O8B—H8AB	106 (7)	O5—Cu2—Cl2	92.52 (6)
Cl1—Cu1—O3	100.94 (7)	O5—Cu2—Cl3	99.27 (6)
O2—Cu1—Cl1	90.43 (6)	O5—Cu2—N11	90.89 (9)
O2—Cu1—O3	100.56 (9)	O5—Cu2—N14	166.34 (9)
O2—Cu1—N4	91.94 (9)	N11—Cu2—Cl2	161.34 (7)
O2—Cu1—N6	166.42 (10)	N11—Cu2—Cl3	94.16 (7)
N4—Cu1—Cl1	170.32 (7)	N11—Cu2—N14	80.60 (10)
N4—Cu1—O3	87.85 (9)	N14—Cu2—Cl2	92.30 (7)
N4—Cu1—N6	81.45 (10)	N14—Cu2—Cl3	92.03 (8)
N6—Cu1—Cl1	94.23 (7)	C12—O4—C13	110.3 (2)
N6—Cu1—O3	91.09 (9)	C19—O5—Cu2	126.25 (18)
C3—O1—C1	110.9 (2)	C15—N9—C14	113.9 (2)
C8—O2—Cu1	125.16 (18)	C16—N9—C14	122.5 (2)
Cu1—O3—H3A	115 (3)	C16—N9—C15	123.1 (2)
Cu1—O3—H3B	136 (4)	C17—N10—C16	114.6 (2)
H3A—O3—H3B	103 (5)	C17—N11—Cu2	115.50 (17)
C2—N1—C4	113.6 (2)	C18—N11—Cu2	130.81 (19)

C5—N1—C2	122.6 (2)	C18—N11—C17	113.0 (2)
C5—N1—C4	123.1 (2)	C18—N12—C16	113.9 (2)
C7—N2—C5	114.0 (2)	N14—N13—H13	119 (2)
C6—N3—C5	114.4 (2)	C17—N13—H13	122 (2)
C6—N4—Cu1	129.78 (19)	C17—N13—N14	117.3 (2)
C6—N4—C7	114.4 (2)	Cu2—N14—H14A	116 (2)
C7—N4—Cu1	115.40 (18)	Cu2—N14—H14B	106 (3)
N6—N5—H5	116 (2)	N13—N14—Cu2	110.25 (17)
C7—N5—H5	126 (2)	N13—N14—H14A	109 (2)
C7—N5—N6	118.1 (2)	N13—N14—H14B	107 (3)
Cu1—N6—H6A	113 (2)	H14A—N14—H14B	108 (3)
Cu1—N6—H6B	109 (2)	N16—N15—C18	120.2 (2)
N5—N6—Cu1	109.22 (17)	N16—N15—C19	108.5 (2)
N5—N6—H6A	105 (2)	C18—N15—C19	131.3 (2)
N5—N6—H6B	109 (2)	N15—N16—H16	124 (3)
H6A—N6—H6B	111 (3)	C21—N16—N15	108.6 (2)
N8—N7—C8	110.9 (2)	C21—N16—H16	127 (3)
C6—N7—N8	119.1 (2)	O4—C12—H12A	109.4
C6—N7—C8	129.9 (2)	O4—C12—H12B	109.4
C10—N8—N7	104.2 (2)	O4—C12—C14	111.2 (2)
O1—C1—H1A	109.2	H12A—C12—H12B	108.0
O1—C1—H1B	109.2	C14—C12—H12A	109.4
O1—C1—C2	112.1 (2)	C14—C12—H12B	109.4
H1A—C1—H1B	107.9	O4—C13—H13A	109.3
C2—C1—H1A	109.2	O4—C13—H13B	109.3
C2—C1—H1B	109.2	O4—C13—C15	111.6 (2)
N1—C2—C1	109.0 (2)	H13A—C13—H13B	108.0
N1—C2—H2A	109.9	C15—C13—H13A	109.3
N1—C2—H2B	109.9	C15—C13—H13B	109.3
C1—C2—H2A	109.9	N9—C14—C12	109.4 (2)
C1—C2—H2B	109.9	N9—C14—H14C	109.8
H2A—C2—H2B	108.3	N9—C14—H14D	109.8
O1—C3—H3C	109.5	C12—C14—H14C	109.8
O1—C3—H3D	109.5	C12—C14—H14D	109.8
O1—C3—C4	110.7 (2)	H14C—C14—H14D	108.2

H3C—C3—H3D	108.1	N9—C15—C13	109.3 (2)
C4—C3—H3C	109.5	N9—C15—H15A	109.8
C4—C3—H3D	109.5	N9—C15—H15B	109.8
N1—C4—C3	109.4 (2)	C13—C15—H15A	109.8
N1—C4—H4A	109.8	C13—C15—H15B	109.8
N1—C4—H4B	109.8	H15A—C15—H15B	108.3
C3—C4—H4A	109.8	N9—C16—N10	118.9 (2)
C3—C4—H4B	109.8	N9—C16—N12	116.7 (2)
H4A—C4—H4B	108.2	N10—C16—N12	124.4 (2)
N1—C5—N2	118.5 (2)	N10—C17—N11	125.8 (2)
N1—C5—N3	116.0 (2)	N10—C17—N13	119.0 (2)
N2—C5—N3	125.5 (3)	N13—C17—N11	115.2 (2)
N3—C6—N4	125.7 (3)	N11—C18—N15	115.5 (2)
N3—C6—N7	117.6 (2)	N12—C18—N11	128.2 (3)
N4—C6—N7	116.6 (2)	N12—C18—N15	116.3 (2)
N2—C7—N4	125.8 (2)	O5—C19—N15	123.8 (2)
N2—C7—N5	118.7 (3)	O5—C19—C20	130.6 (3)
N5—C7—N4	115.4 (2)	N15—C19—C20	105.6 (2)
O2—C8—N7	124.7 (2)	C19—C20—H20	126.0
O2—C8—C9	129.7 (3)	C21—C20—C19	108.1 (2)
C9—C8—N7	105.6 (2)	C21—C20—H20	126.0
C8—C9—H9	126.9	N16—C21—C20	109.2 (2)
C8—C9—C10	106.2 (3)	N16—C21—C22	120.4 (2)
C10—C9—H9	126.9	C20—C21—C22	130.3 (3)
N8—C10—C9	113.1 (2)	C21—C22—H22A	109.5
N8—C10—C11	119.9 (3)	C21—C22—H22B	109.5
C9—C10—C11	127.0 (3)	C21—C22—H22C	109.5
C10—C11—H11A	109.5	H22A—C22—H22B	109.5
C10—C11—H11B	109.5	H22A—C22—H22C	109.5
C10—C11—H11C	109.5	H22B—C22—H22C	109.5
H11A—C11—H11B	109.5	H6C—O6—H6D	109 (5)
H11A—C11—H11C	109.5	H7A—O7—H7B	101 (5)
H11B—C11—H11C	109.5		

Symmetry code: (i) -*x*, -*y*, -*z*+2.

Table S3. Geometric parameters *i.e.* bond lengths and angles (Å, º) for **7**.

Bond lengths			
O6A—O6A^{i}	1.16 (19)	N8—H8B	0.9100
C19A—C20A	1.42 (6)	N9—H9A	0.9100
C19A—N14	1.49 (6)	N9—H9B	0.9100
C20A—O4A	1.44 (3)	N9—N10	1.413 (9)
C21A—C22A	1.46 (2)	N10—C17	1.348 (11)
C21A—N14	1.478 (16)	N10—H10	0.89 (9)
C22A—O4A	1.68 (3)	N11—C16	1.344 (10)
O6B—O6B^{i}	1.7 (2)	N11—C17	1.347 (10)
C19B—C20B	1.05 (6)	N12—C17	1.323 (10)
C19B—N14	1.49 (8)	N12—C18	1.348 (10)
C20B—O4B	1.45 (3)	N13—C16	1.307 (9)
C21B—C22B	1.59 (3)	N13—C18	1.362 (10)
C21B—N14	1.539 (19)	N14—C18	1.340 (10)
C22B—O4B	1.66 (4)	N15—N16	1.376 (9)
Ni—O2	2.053 (5)	N15—C12	1.416 (9)
Ni—O3	2.034 (6)	N15—C16	1.398 (10)
Ni—N4	2.018 (6)	N16—C14	1.337 (10)
Ni—N8	2.096 (7)	N16—H16	0.79 (8)
Ni—N9	2.092 (7)	C1—H1A	0.9900
Ni—N11	2.000 (6)	C1—H1B	0.9900
O1—C1	1.423 (11)	C1—C3	1.473 (13)
O1—C2	1.405 (11)	C2—H2A	0.9900
O2—C11	1.262 (9)	C2—H2B	0.9900
O3—C12	1.247 (9)	C2—C4	1.440 (14)
N1—C3	1.495 (11)	C3—H3A	0.9900
N1—C4	1.467 (11)	C3—H3B	0.9900
N1—C5	1.332 (10)	C4—H4A	0.9900
N2—C5	1.333 (10)	C4—H4B	0.9900
N2—C6	1.326 (9)	C8—H8C	0.9800
N3—C5	1.372 (10)	C8—H8D	0.9800
N3—C7	1.313 (9)	C8—H8E	0.9800
N4—C6	1.356 (9)	C8—C9	1.489 (12)
N4—C7	1.324 (9)	C9—C10	1.372 (12)

N5—N6	1.374 (9)	C10—H10A	0.9500
N5—C9	1.333 (11)	C10—C11	1.400 (11)
N5—H5	0.94 (9)	C12—C13	1.406 (12)
N6—C7	1.401 (10)	C13—H13	0.9500
N6—C11	1.417 (9)	C13—C14	1.378 (11)
N7—N8	1.417 (9)	C14—C15	1.482 (11)
N7—C6	1.321 (10)	C15—H15A	0.9800
N7—H7	0.93 (8)	C15—H15B	0.9800
N8—H8A	0.9100	C15—H15C	0.9800
Bond angles			
C20A—C19A—N14	114 (4)	C14—N16—N15	108.7 (6)
C19A—C20A—O4A	123 (2)	C14—N16—H16	136 (6)
C22A—C21A—N14	106.2 (13)	O1—C1—H1A	109.4
C21A—C22A—O4A	102.0 (16)	O1—C1—H1B	109.4
C20A—O4A—C22A	111.8 (14)	O1—C1—C3	111.3 (8)
C20B—C19B—N14	131 (6)	H1A—C1—H1B	108.0
C19B—C20B—O4B	117 (5)	C3—C1—H1A	109.4
N14—C21B—C22B	103.2 (14)	C3—C1—H1B	109.4
C21B—C22B—O4B	89.6 (16)	O1—C2—H2A	109.1
C20B—O4B—C22B	118.0 (19)	O1—C2—H2B	109.1
O2—Ni—N8	169.4 (2)	O1—C2—C4	112.5 (8)
O2—Ni—N9	90.1 (2)	H2A—C2—H2B	107.8
O3—Ni—O2	91.3 (2)	C4—C2—H2A	109.1
O3—Ni—N8	88.9 (3)	C4—C2—H2B	109.1
O3—Ni—N9	170.1 (2)	N1—C3—H3A	109.8
N4—Ni—O2	89.4 (2)	N1—C3—H3B	109.8
N4—Ni—O3	91.0 (2)	C1—C3—N1	109.3 (8)
N4—Ni—N8	80.0 (2)	C1—C3—H3A	109.8
N4—Ni—N9	98.9 (2)	C1—C3—H3B	109.8
N9—Ni—N8	91.5 (3)	H3A—C3—H3B	108.3
N11—Ni—O2	93.4 (2)	N1—C4—H4A	109.5
N11—Ni—O3	90.0 (2)	N1—C4—H4B	109.5
N11—Ni—N4	177.0 (3)	C2—C4—N1	110.9 (8)
N11—Ni—N8	97.1 (3)	C2—C4—H4A	109.5
N11—Ni—N9	80.1 (2)	C2—C4—H4B	109.4

C2—O1—C1	111.1 (7)	H4A—C4—H4B	108.0
C11—O2—Ni	126.4 (5)	N1—C5—N2	118.1 (7)
C12—O3—Ni	125.8 (5)	N1—C5—N3	117.4 (7)
C4—N1—C3	113.4 (7)	N2—C5—N3	124.6 (7)
C5—N1—C3	121.6 (7)	N2—C6—N4	124.5 (7)
C5—N1—C4	121.7 (7)	N7—C6—N2	117.7 (7)
C6—N2—C5	115.7 (7)	N7—C6—N4	117.7 (6)
C7—N3—C5	113.5 (6)	N3—C7—N4	127.4 (7)
C6—N4—Ni	114.2 (5)	N3—C7—N6	115.6 (6)
C7—N4—Ni	131.1 (5)	N4—C7—N6	116.9 (6)
C7—N4—C6	114.3 (6)	H8C—C8—H8D	109.5
N6—N5—H5	121 (5)	H8C—C8—H8E	109.5
C9—N5—N6	108.6 (6)	H8D—C8—H8E	109.5
C9—N5—H5	131 (5)	C9—C8—H8C	109.5
N5—N6—C7	119.7 (6)	C9—C8—H8D	109.5
N5—N6—C11	108.1 (6)	C9—C8—H8E	109.5
C7—N6—C11	132.2 (6)	N5—C9—C8	121.4 (8)
N8—N7—H7	124 (5)	N5—C9—C10	110.1 (7)
C6—N7—N8	119.2 (6)	C10—C9—C8	128.5 (8)
C6—N7—H7	117 (5)	C9—C10—H10A	126.1
Ni—N8—H8A	110.0	C9—C10—C11	107.8 (7)
Ni—N8—H8B	110.0	C11—C10—H10A	126.1
N7—N8—Ni	108.7 (5)	O2—C11—N6	123.7 (7)
N7—N8—H8A	110.0	O2—C11—C10	130.8 (7)
N7—N8—H8B	110.0	C10—C11—N6	105.5 (6)
H8A—N8—H8B	108.3	O3—C12—N15	124.8 (7)
Ni—N9—H9A	110.1	O3—C12—C13	130.0 (7)
Ni—N9—H9B	110.1	C13—C12—N15	105.2 (6)
H9A—N9—H9B	108.4	C12—C13—H13	126.0
N10—N9—Ni	108.2 (5)	C14—C13—C12	108.1 (7)
N10—N9—H9A	110.1	C14—C13—H13	126.0
N10—N9—H9B	110.1	N16—C14—C13	109.6 (7)
N9—N10—H10	111 (6)	N16—C14—C15	120.1 (7)
C17—N10—N9	120.0 (7)	C13—C14—C15	130.3 (7)
C17—N10—H10	129 (6)	C14—C15—H15A	109.5

C16—N11—Ni	131.2 (5)	C14—C15—H15B	109.5
C16—N11—C17	112.7 (6)	C14—C15—H15C	109.5
C17—N11—Ni	116.0 (5)	H15A—C15—H15B	109.5
C17—N12—C18	113.9 (7)	H15A—C15—H15C	109.5
C16—N13—C18	113.8 (7)	H15B—C15—H15C	109.5
C21A—N14—C19A	119 (2)	N11—C16—N15	115.9 (6)
C19B—N14—C21B	106 (2)	N13—C16—N11	127.6 (7)
C18—N14—C19A	119 (2)	N13—C16—N15	116.4 (7)
C18—N14—C21A	121.4 (8)	N11—C17—N10	115.6 (7)
C18—N14—C19B	125 (3)	N12—C17—N10	117.6 (7)
C18—N14—C21B	120.0 (9)	N12—C17—N11	126.8 (8)
N16—N15—C12	108.3 (6)	N12—C18—N13	125.1 (7)
N16—N15—C16	119.1 (6)	N14—C18—N12	118.2 (7)
C16—N15—C12	131.7 (7)	N14—C18—N13	116.7 (7)
N15—N16—H16	110 (6)		

Symmetry code: (i) -*x*+1, -*y*, -*z*+2.

Table S4. Geometric parameters *i.e.* bond lengths and angles (Å, º) for **8**.

Bond lengths			
O13A—O9A	1.74 (3)	N14—C14	1.360 (7)
O13A—O10A	1.25 (3)	N14—H14	0.81 (7)
Zn—O2	2.066 (4)	N15—N16	1.422 (6)
Zn—O3	2.048 (4)	N15—C17	1.336 (7)
Zn—N3	2.082 (4)	N15—H15	0.81 (6)
Zn—N6	2.191 (5)	N16—H16A	0.93 (5)
Zn—N12	2.092 (4)	N16—H16B	0.81 (6)
Zn—N16	2.172 (5)	C1—H1A	0.9900
O1—C1	1.421 (7)	C1—H1B	0.9900
O1—C2	1.432 (7)	C1—C4	1.527 (7)
O2—C12	1.253 (6)	C2—H2A	0.9900
O3—C8	1.277 (6)	C2—H2B	0.9900
O4—C20	1.418 (7)	C2—C3	1.514 (7)
O4—C21	1.425 (7)	C3—H3A	0.9900
N1—C3	1.454 (7)	C3—H3B	0.9900
N1—C4	1.462 (7)	C4—H4A	0.9900
N1—C5	1.358 (6)	C4—H4B	0.9900
N2—C5	1.340 (7)	C8—C9	1.377 (8)
N2—C7	1.338 (6)	C9—H9	0.9500
N3—C6	1.343 (7)	C9—C10	1.402 (8)
N3—C7	1.348 (7)	C10—C11	1.488 (9)
N4—C5	1.347 (7)	C11—H11A	0.9800
N4—C6	1.322 (6)	C11—H11B	0.9800
N5—N6	1.421 (6)	C11—H11C	0.9800
N5—C7	1.334 (7)	C12—C13	1.403 (8)
N5—H5	0.89 (6)	C13—H13	0.9500
N6—H6A	0.85 (6)	C13—C14	1.371 (7)
N6—H6B	0.90 (7)	C14—C15	1.477 (7)
N7—N8	1.405 (6)	C15—H15A	0.9800
N7—C6	1.378 (7)	C15—H15B	0.9800
N7—C8	1.417 (6)	C15—H15C	0.9800
N8—C10	1.331 (7)	C19—H19A	0.9900
N9—C18	1.346 (6)	C19—H19B	0.9900

N9—C19	1.463 (7)	C19—C20	1.507 (8)
N9—C22	1.463 (7)	C20—H20A	0.9900
N10—C16	1.321 (6)	C20—H20B	0.9900
N10—C18	1.363 (7)	C21—H21A	0.9900
N11—C17	1.338 (6)	C21—H21B	0.9900
N11—C18	1.336 (7)	C21—C22	1.509 (8)
N12—C16	1.332 (6)	C22—H22A	0.9900
N12—C17	1.345 (7)	C22—H22B	0.9900
N13—N14	1.390 (6)	O5—H5A	0.99 (6)
N13—C12	1.406 (6)	O5—H5B	0.99 (2)
N13—C16	1.385 (7)		
Bond angles			
O10A—O13A—O9A	134.2 (17)	H3A—C3—H3B	108.3
O2—Zn—N3	92.59 (15)	N1—C4—C1	109.3 (5)
O2—Zn—N6	85.21 (19)	N1—C4—H4A	109.8
O2—Zn—N12	86.38 (15)	N1—C4—H4B	109.8
O2—Zn—N16	162.92 (16)	C1—C4—H4A	109.8
O3—Zn—O2	98.17 (15)	C1—C4—H4B	109.8
O3—Zn—N3	87.01 (15)	H4A—C4—H4B	108.3
O3—Zn—N6	165.00 (15)	N2—C5—N1	116.2 (5)
O3—Zn—N12	90.89 (15)	N2—C5—N4	126.5 (4)
O3—Zn—N16	89.43 (18)	N4—C5—N1	117.3 (5)
N3—Zn—N6	78.21 (17)	N3—C6—N7	117.6 (4)
N3—Zn—N12	177.51 (18)	N4—C6—N3	126.1 (5)
N3—Zn—N16	103.11 (17)	N4—C6—N7	116.3 (5)
N12—Zn—N6	103.94 (17)	N2—C7—N3	125.9 (5)
N12—Zn—N16	78.19 (16)	N5—C7—N2	115.0 (5)
N16—Zn—N6	91.4 (2)	N5—C7—N3	119.0 (4)
C1—O1—C2	110.3 (4)	O3—C8—N7	124.2 (5)
C12—O2—Zn	128.8 (3)	O3—C8—C9	130.7 (5)
C8—O3—Zn	128.2 (3)	C9—C8—N7	105.0 (5)
C20—O4—C21	110.6 (4)	C8—C9—H9	126.3
C3—N1—C4	115.3 (4)	C8—C9—C10	107.4 (5)
C5—N1—C3	120.8 (5)	C10—C9—H9	126.3
C5—N1—C4	121.5 (4)	N8—C10—C9	112.4 (5)

C7—N2—C5	113.4 (4)	N8—C10—C11	120.7 (5)
C6—N3—Zn	131.2 (3)	C9—C10—C11	126.8 (5)
C6—N3—C7	114.0 (4)	C10—C11—H11A	109.5
C7—N3—Zn	114.7 (3)	C10—C11—H11B	109.5
C6—N4—C5	113.9 (4)	C10—C11—H11C	109.5
N6—N5—H5	119 (4)	H11A—C11—H11B	109.5
C7—N5—N6	119.9 (5)	H11A—C11—H11C	109.5
C7—N5—H5	121 (4)	H11B—C11—H11C	109.5
Zn—N6—H6A	120 (4)	O2—C12—N13	123.9 (5)
Zn—N6—H6B	107 (4)	O2—C12—C13	129.9 (5)
N5—N6—Zn	108.0 (3)	C13—C12—N13	106.2 (4)
N5—N6—H6A	107 (4)	C12—C13—H13	125.9
N5—N6—H6B	112 (4)	C14—C13—C12	108.2 (4)
H6A—N6—H6B	103 (6)	C14—C13—H13	125.9
N8—N7—C8	110.7 (4)	N14—C14—C13	109.5 (5)
C6—N7—N8	118.1 (4)	N14—C14—C15	120.8 (4)
C6—N7—C8	131.1 (4)	C13—C14—C15	129.7 (5)
C10—N8—N7	104.4 (4)	C14—C15—H15A	109.5
C18—N9—C19	122.1 (4)	C14—C15—H15B	109.5
C18—N9—C22	122.0 (4)	C14—C15—H15C	109.5
C22—N9—C19	113.9 (4)	H15A—C15—H15B	109.5
C16—N10—C18	114.0 (4)	H15A—C15—H15C	109.5
C18—N11—C17	114.7 (4)	H15B—C15—H15C	109.5
C16—N12—Zn	131.5 (3)	N10—C16—N12	126.9 (5)
C16—N12—C17	113.8 (4)	N10—C16—N13	116.2 (4)
C17—N12—Zn	114.5 (3)	N12—C16—N13	116.8 (4)
N14—N13—C12	108.1 (4)	N11—C17—N12	125.6 (5)
C16—N13—N14	118.9 (4)	N15—C17—N11	116.4 (5)
C16—N13—C12	132.3 (4)	N15—C17—N12	118.0 (4)
N13—N14—H14	117 (5)	N9—C18—N10	116.8 (4)
C14—N14—N13	107.9 (4)	N11—C18—N9	118.3 (5)
C14—N14—H14	125 (5)	N11—C18—N10	124.9 (4)
N16—N15—H15	119 (4)	N9—C19—H19A	109.8
C17—N15—N16	120.3 (5)	N9—C19—H19B	109.8
C17—N15—H15	121 (4)	N9—C19—C20	109.6 (5)

Zn—N16—H16A	118 (3)	H19A—C19—H19B	108.2
Zn—N16—H16B	108 (4)	C20—C19—H19A	109.8
N15—N16—Zn	107.5 (3)	C20—C19—H19B	109.8
N15—N16—H16A	104 (3)	O4—C20—C19	111.5 (5)
N15—N16—H16B	107 (4)	O4—C20—H20A	109.3
H16A—N16—H16B	113 (5)	O4—C20—H20B	109.3
O1—C1—H1A	109.3	C19—C20—H20A	109.3
O1—C1—H1B	109.3	C19—C20—H20B	109.3
O1—C1—C4	111.7 (5)	H20A—C20—H20B	108.0
H1A—C1—H1B	107.9	O4—C21—H21A	109.2
C4—C1—H1A	109.3	O4—C21—H21B	109.2
C4—C1—H1B	109.3	O4—C21—C22	111.8 (5)
O1—C2—H2A	109.3	H21A—C21—H21B	107.9
O1—C2—H2B	109.3	C22—C21—H21A	109.2
O1—C2—C3	111.6 (5)	C22—C21—H21B	109.2
H2A—C2—H2B	108.0	N9—C22—C21	110.3 (5)
C3—C2—H2A	109.3	N9—C22—H22A	109.6
C3—C2—H2B	109.3	N9—C22—H22B	109.6
N1—C3—C2	109.0 (5)	C21—C22—H22A	109.6
N1—C3—H3A	109.9	C21—C22—H22B	109.6
N1—C3—H3B	109.9	H22A—C22—H22B	108.1
C2—C3—H3A	109.9	H5A—O5—H5B	104 (6)
C2—C3—H3B	109.9		
